# Liquid exfoliation of solvent-stabilized few-layer black phosphorus for applications beyond electronics

**DOI:** 10.1038/ncomms9563

**Published:** 2015-10-15

**Authors:** Damien Hanlon, Claudia Backes, Evie Doherty, Clotilde S. Cucinotta, Nina C. Berner, Conor Boland, Kangho Lee, Andrew Harvey, Peter Lynch, Zahra Gholamvand, Saifeng Zhang, Kangpeng Wang, Glenn Moynihan, Anuj Pokle, Quentin M. Ramasse, Niall McEvoy, Werner J. Blau, Jun Wang, Gonzalo Abellan, Frank Hauke, Andreas Hirsch, Stefano Sanvito, David D. O'Regan, Georg S. Duesberg, Valeria Nicolosi, Jonathan N. Coleman

**Affiliations:** 1 School of Physics, Trinity College Dublin, Dublin 2, Ireland; 2 CRANN and AMBER Research Centres, Trinity College Dublin, Dublin 2, Ireland; 3 School of Chemistry, Trinity College Dublin, Dublin 2, Ireland; 4 Key Laboratory of Materials for High-Power Laser, Shanghai Institute of Optics and Fine Mechanics, Chinese Academy of Sciences, Shanghai 201800, China; 5 SuperSTEM Laboratory, STFC Daresbury Campus, Daresbury WA4 4AD, UK; 6 Chair of Organic Chemistry II and Institute of Advanced Materials and Processes (ZMP), Friedrich-Alexander-Universität Erlangen-Nürnberg (FAU), Henkestrasse 42, 91054 Erlangen, Germany

## Abstract

Few-layer black phosphorus (BP) is a new two-dimensional material which is of great interest for applications, mainly in electronics. However, its lack of environmental stability severely limits its synthesis and processing. Here we demonstrate that high-quality, few-layer BP nanosheets, with controllable size and observable photoluminescence, can be produced in large quantities by liquid phase exfoliation under ambient conditions in solvents such as *N*-cyclohexyl-2-pyrrolidone (CHP). Nanosheets are surprisingly stable in CHP, probably due to the solvation shell protecting the nanosheets from reacting with water or oxygen. Experiments, supported by simulations, show reactions to occur only at the nanosheet edge, with the rate and extent of the reaction dependent on the water/oxygen content. We demonstrate that liquid-exfoliated BP nanosheets are potentially useful in a range of applications from ultrafast saturable absorbers to gas sensors to fillers for composite reinforcement.

## Abstract (web summary)

While phosphorene is an exciting new 2D material, techniques to produce it in large quantities in a stable, processable form are lacking. Here, the authors achieve this using liquid phase exfoliation and demonstrate the resultant nanosheets to be useful in a number of applications.

## Graphical abstract

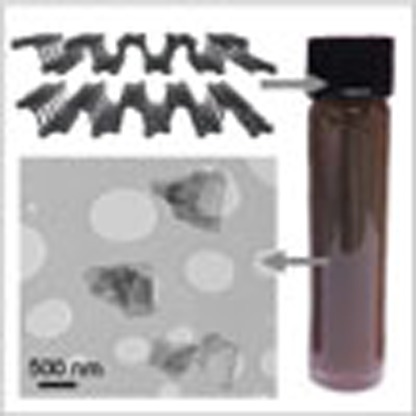

Over the last few years, the study of two-dimensional (2D) materials^1^,^2^,^3^,^4^,^5^,^6^ such as graphene, BN and MoS_2_ have become one of the most exciting areas of nano-science. However, in the past year a new 2D material has been generating considerable excitement in the research community^7^. Phosphorene consists of atomically thin, 2D nanosheets of black phosphorus (BP). In BP, the monolayers stack together via van der Waals interactions to form layered crystals in much the same way as graphene stacks together to form graphite. Recently, it was shown that BP can be exfoliated by mechanical cleavage to form mono- and few-layer phosphorene, which we refer to as FL-BP^8^,^9^,^10^,^11^,^12^. This new material has a direct bandgap in mono-, few-layer and bulk forms, which varies with nanosheet thickness from ∼1.5 eV for monolayer phosphorene to ∼0.3 eV for bulk BP^8^,^13^,^14^. This is in contrast to graphene^1^ which has no bandgap and materials such as MoS_2_, which display direct bandgaps only in the monolayer form^6^. As a result, BP is extremely attractive both for electronics and optoelectronics and has therefore been extensively studied in applications such as transistors^7^,^12^,^15^, photodetectors^15^,^16^ and solar cells^9^.

In addition, like other 2D materials, it is probable that BP has the potential to perform in a range of applications beyond (opto)electronics. Indeed, BP has already been fabricated into electrodes in lithium ion batteries^17^. Furthermore, theory predicts that FL-BP shows potential for use in gas sensors^18^ and thermoelectrics^19^. For most applications, it will be necessary to produce FL-BP in much larger quantities than can be achieved by mechanical exfoliation. One way to prepare nanosheets in large quantities is by liquid phase exfoliation (LPE)^20^,^21^. This technique involves the sonication^22^,^23^ or shearing^24^,^25^ of layered crystals in appropriate liquids and has previously been applied to graphene and boron nitride, as well as a range of other 2D materials^21^,^22^,^23^,^26^,^27^,^28^.

While phosphorene nanosheets have very recently been produced by liquid exfoliation^29^,^30^,^31^, this method remains problematic, largely because BP is known to be unstable^7^,^8^,^32^, degrading via reactions with water and oxygen. For this method to be useful, ways must be found to stabilize liquid-exfoliated FL-BP nanosheets against oxidation. It is known that BP can be protected from reacting with environmental species by encapsulation, suggesting a possible way forward^7^,^24^. We hypothesized that LPE of BP may be practical if the solvent is carefully chosen to minimize oxidation of the exfoliated nanosheets in the liquid phase due to the solvation shell acting as a barrier to prevent oxidative species reaching the nanosheet surface.

If this could be achieved it would yield numerous advantages. LPE is a powerful technique to produce nanosheets in large quantities. In addition, the nanosheets are produced directly in the liquid phase and are thus inherently processable and can be easily formed into composites, coatings or films^21^, facilitating their use in a range of applications. Furthermore, size-control can be achieved by centrifugation, enabling processing for both applications and fundamental studies. For example, studying the oxidation of BP nanosheets of varying sizes would give insight into whether the degradation mechanism is associated with nanosheet edges or the basal plane. However, most importantly, protection of the nanosheets by an appropriate solvent would allow large-scale production in ambient conditions, dramatically simplifying future development of this material.

In this work, we demonstrate that BP can be exfoliated to give large quantities of FL-BP nanosheets by sonication in solvents such as *N*-cyclohexyl-2-pyrrolidone (CHP), even in ambient conditions. We show that these nanosheets can be readily size-selected by controlled centrifugation. Such dispersions show direct gap photoluminescence (PL) with PL energy, width and relative quantum yield scaling strongly with layer number. The BP nanosheets are relatively stable in CHP, but degrade rapidly once water/O_2_ is added. The reaction occurs predominantly at nanosheet edges, presumably following a different reaction pathway than previously observed^32^. Finally we demonstrate that liquid-exfoliated BP nanosheets can be used in applications beyond electronics. We show that dispersions of FL-BP have impressive nonlinear optical properties allowing them to be used in optical switching applications. In addition, the nanosheets can be incorporated into polymer matrices resulting in reinforced composites and are highly potent gas sensors.

## Results

### Exfoliation and basic characterization

**Figure 1:**
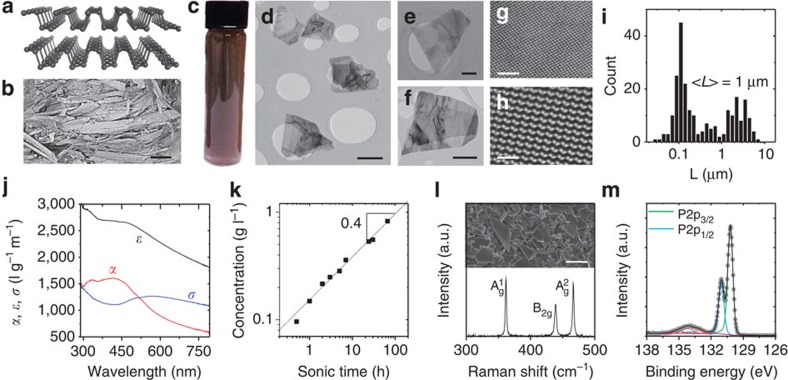
Basic characterization of exfoliated black phosphorous. (**a**) Structure of black phosphorus (BP). (**b**) SEM image of a layered BP crystal (scale bar, 100 μm). (**c**) Photograph of a dispersion of exfoliated FL-BP in CHP. (**d**–**f**) Representative low-resolution transmission electron microscopy (TEM) images of FL-BP exfoliated in *N*-cyclohexyl-2-pyrrolidone (CHP) (scale bars in **d**–**f**: 500, 100 and 500 nm). (**g**) Bright-field scanning transmission TEM (STEM) image and (**h**) Butterworth-filtered high-angle annular dark field (HAADF) STEM image of FL-BP (exfoliated in isopropanol) showing the intact lattice (scale bars in **g** and **h**, 2 and 1 nm). (**i**) Nanosheet length histogram of the exfoliated FL-BP obtained from TEM (sample size=239). (**j**) Extinction, absorbance, scattering coefficient spectra of FL-BP in CHP. (**k**) Concentration of FL-BP as a function of sonication time. The dashed line shows power law behaviour with exponent 0.4. (**l**) Raman spectrum (mean of 100 spectra, excitation 633 nm) of a filtered dispersion. Inset: scanning electron microscopic image of thin film (scale bar, 2 μm). (**m**) X-ray photoelectron spectroscopy P core-level region.

To produce large quantities of BP nanosheets (see Fig. 1a for structure), we use LPE^22^,^23^. This technique is often carried out in amide solvents such as CHP or *N*-methyl-2-pyrrolidone (NMP), although isopropanol (IPA) has proven useful in some cases^22^. Sonication of ground BP crystals (see Fig. 1b for scanning electron microscopic (SEM) image) in CHP yields a brown dispersion (Fig. 1c). In the simplest case, we remove unexfoliated material by centrifugation at 1,000 r.p.m. (106 *g*) for 180 min to yield a stable dispersion which we refer to as the standard sample (std-BP, see Supplementary Fig. 1 for process optimization).

The successful exfoliation of BP in CHP was confirmed by transmission electron microscopy (TEM) of nanosheets on holey carbon grids (TEM, Fig. 1d–f and Supplementary Fig. 2). These images show electron-transparent nanosheets with lateral dimensions *L*∼1 μm. As shown by scanning TEM (STEM) and high-angle annular dark field (HAADF) STEM imaging, the lattice appears intact over wide regions (Fig. 1g,h). This suggests that, as with other 2D materials, BP can be exfoliated in liquids without the introduction of defects^21^,^22^,^23^,^24^,^33^. N.B. we used FL-BP exfoliated in IPA for STEM and HAADF, due to difficulties in completely removing the CHP. However, the intact lattice was also observed from the std-BP in CHP (Supplementary Fig. 3). To assess the lateral dimensions of the std-BP, we performed statistical TEM analysis, finding a bimodal size distribution with modes associated with *L*∼100 nm and *L*∼3 μm (Fig. 1i). We note that the larger nanosheets are considerably bigger than is typically observed for other 2D materials (for example, *L*<1 μm)^22^,^26^,^33^,^34^.

To further characterize the std-BP dispersion, we measured the extinction coefficient, *ɛ*, as shown in Fig. 1j. This parameter is defined via the optical transmittance; *T*=10^−*ɛCl*^, where *l* is the cell length and *C* is the nanosheet concentration (measured by filtration and weighing). The extinction coefficient includes contributions from both absorbance (*α*) and scattering (*σ*)^33^,^35^, which can be isolated using an integrating sphere and are shown in Fig. 1j. Note that coefficients of extinction, absorbance and scattering are related by *ɛ*(*λ*)=*α*(*λ*)+*σ*(*λ*)^33^,^35^. Because of size-dependent scattering contributions, the extinction coefficient cannot be used to accurately measure nanosheet concentration of varying sizes^33^,^34^,^35^. Instead, we can use the measured absorbance coefficient (*α*(*λ*=465 nm)=15 l g^−1^ cm^−1^) to give the concentration of all subsequent FL-BP dispersions. For example, Fig. 1k shows the BP concentration measured this way to scale with the sonication time as *C*∝*t*^0.4^, similar to previous observations for graphene exfoliation^24^,^36^. Concentrations as high as ∼1 g l^−1^ can easily be realized.

To further confirm the structural integrity of the FL-BP, the dispersion was filtered onto alumina membranes and subjected to Raman and X-ray photoelectron spectroscopy (XPS). SEM confirmed the homogeneity of the film (Fig. 1l inset). The Raman spectrum (Fig. 1l) shows the characteristic A_g_^1^, B_2g_ and A_g_^2^ phonons of FL-BP^8^,^12^,^13^,^32^. The P2p XPS core-level spectra (Fig. 1m) show the expected contributions from P2p_1/2_ and P2p_3/2_ components. Contributions from P_*x*_O_*y*_ species, presumably as a result of partial degradation (discussed below) are minor (<15%).

**Figure 2:**
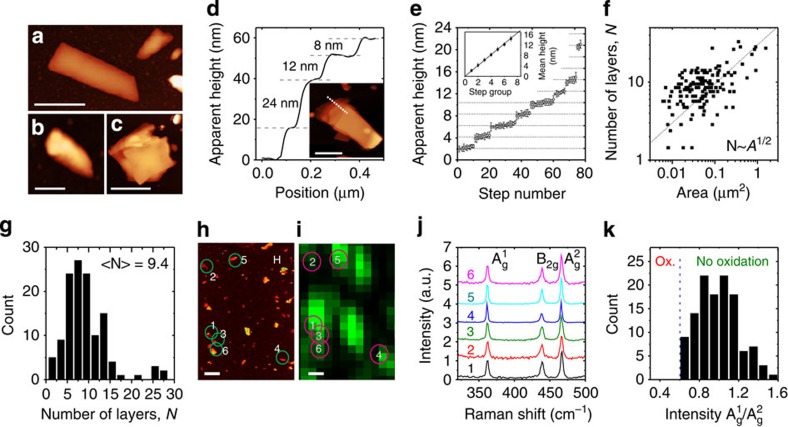
Characterization of individual nanosheets. (**a**–**c**) Representative atomic force microscopic (AFM) images (scale bars in **a**, **b** and **c:** are 500, 200 and 1000, nm). (**d**) Height profile of the nanosheet in the inset along the line showing clearly resolvable steps, each consisting of multiple monolayers (scale bar, 500 nm). (**e**) Heights of >70 steps of deposited FL-BP nanosheets in ascending order. The step height clustered in groups and is always found to be a multiple of ∼2 nm, which is the apparent height of one monolayer. The mean height for each group (the error is the sum of the mean step height error and the s.d. in step height within a given group) is plotted in ascending order in the inset with the slope giving a mean monolayer step height of 2.06±0.18 nm. (**f**) Plot of number of layers per nanosheet (obtained by dividing the apparent height by the step height) as a function of flake area determined from AFM. The dashed line indicates 
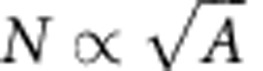
 behaviour. (**g**) Histogram of number of monolayers per nanosheet (sample size=126). The mean number of layers is determined as 9.4±1.3 nm (where the error is due to the uncertainty in the step height analysis and the s.e. of the distribution). (**h**,**i**) Large area AFM image (**h**, scale bar, 1 μm) and Raman A^1^_g_ intensity map (**i**, excitation wavelength 633 nm) of the same sample region. (**j**) Raman spectra (normalized to A_g_^2^) of the nanosheets indicated in **h** and **i** (the numbers labelling the spectra in **j** correspond to the nanosheets marked by numbers in **h** and **i**). (**k**) Histogram of the intensity ratio of the A_g_^1^/A_g_^2^ modes obtained from the analysis of 120 baseline-corrected spectra acquired over an area of 25 × 25 μm^2^ (sample size=120) The absence of spectra with an intensity ratio <0.6 strongly suggests that no basal plane oxidation has occurred^32^.

We can obtain information about the nanosheet thickness using statistical atomic force microscopy (AFM) analysis (images Fig. 2a–c and Supplementary Fig. 4). To overcome the problems associated with deposition of nanosheets onto substrates from high-boiling point solvents, the std-BP in CHP was transferred to IPA prior to drop casting the samples (see Methods). Apparent AFM heights from liquid-exfoliated nanomaterials are usually overestimated due to residual solvent^24^,^33^,^37^, as well as contributions from effects such as capillary forces and adhesion^38^,^39^. To overcome these problems and to convert the apparent measured AFM thickness to the number of layers, we have applied a similar approach to that reported for graphene and MoS_2_ (refs 26, 27). This involves AFM analysis of incompletely exfoliated nanosheets (Fig. 2d inset), measuring the height of the steps between terraces (Fig. 2d and Supplementary Fig. 5). By plotting the measured step heights in ascending order (Fig. 2e), groups of steps with similar apparent heights appear. It is clear that the apparent step height is always a multiple of ∼2 nm, a value which we associate with the apparent monolayer height^24^,^33^. By plotting the mean apparent height associated with each group in ascending order (Fig. 5e inset), we find the apparent monolayer thickness to be 2.06±0.18 nm even though the real thickness is ∼0.5–0.7 nm. We can use this information to convert the measured apparent AFM heights into number of layers, *N*. We then determine the mean number of layers of std-BP to be <*N*>=9.4±1.3 (see Supplementary Fig. 6 for the impact of uncertainty in apparent monolayer height). Shown in Fig. 2f is a plot of *N* versus nanosheet area for std-BP, demonstrating a rough correlation between flake thickness and area: 
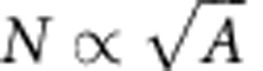
, as previously observed for exfoliated MoO_3_ and GaS nanosheets^26^,^40^. The nanosheets are reasonably thin: ∼70% of the observed nanosheets have *N*≤10 (Fig. 2g).

To gain further insights into the spectroscopic properties of our LPE FL-BP, we have selected a sample area with nanosheets of varying sizes and thicknesses by AFM (Fig. 2h) and relocated the same area under a Raman microscope (Supplementary Fig. 7). A spatial Raman map (A_g_^1^ intensity, excitation wavelength 633 nm) of the region is shown in Fig. 2i. Single spectra (normalized to the A_g_^1^ mode) extracted at the positions indicated in the Figure are displayed in Fig. 2j. These roughly correspond to the nanosheets circled in Fig. 2i. It has been shown that the intensity ratio of the A_g_^1^/A_g_^2^ phonon sensitively depends on sample degradation as a result of oxidation^32^. We therefore analysed the A_g_^1^/A_g_^2^ intensity ratio of 120 individual Raman spectra after baseline subtraction. The resultant intensity ratio histogram is plotted in Fig. 2k. None of the spectra showed A_g_^1^/A_g_^2^<0.6, confirming the basal planes to be unoxidized^32^. Analysis of data collected using 532 nm excitation gives very similar results (Supplementary Fig. 8). Furthermore, oxidation typically gives rise to a broad component under the B_2g_ and A_g_^2^ modes^32^ which is not observed in our samples, further confirming the structural integrity.

### Size selection

**Figure 3:**
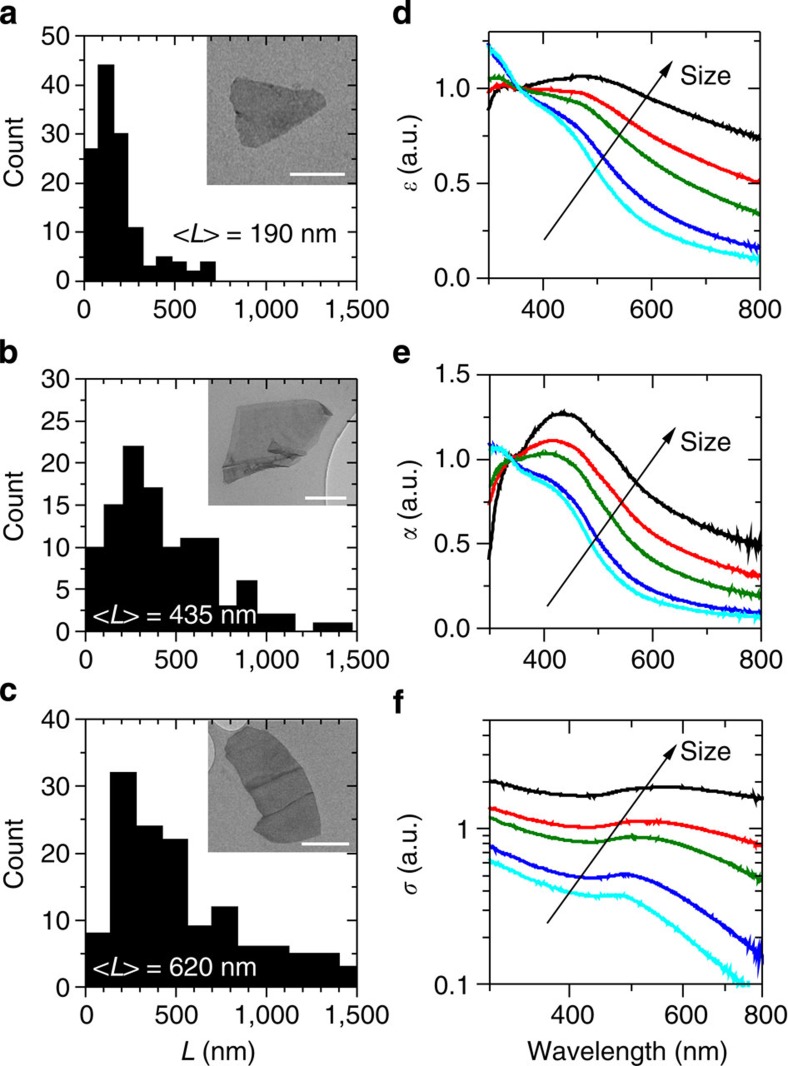
Size dependence of optical properties of exfoliated Black Phosphorous. (**a**–**c**) TEM length histograms of size-selected FL-BP in CHP including representative TEM images as insets (scale bars in **a**–**c**: 100, 200 and 200 nm). Sample sizes in **a**–**c** are 131, 111 and 140, respectively. (**d**) Extinction (*ɛ*) spectra normalized to 355 nm of FL-BP dispersions with different mean nanosheet lengths showing systematic changes as a function of size. Extinction spectra can be split into contributions from absorbance (*α*) and scattering (*σ*). (**e**) Absorbance spectra of the same dispersions (normalized to 340 nm) and (**f**) scattering spectra. Scattering spectra were obtained by subtracting the absorbance spectra from the normalized extinction spectra.

A great advantage of liquid exfoliation is the ability to perform size-selection^24^,^33^,^41^. This is important, as some applications (for example, mechanical reinforcement)^42^ require large nanosheets, while others (for example, catalysis)^43^ benefit from small nanosheets. To demonstrate that established size-selection techniques can be applied to liquid-exfoliated FL-BP, we have performed controlled centrifugation to obtain dispersions with varying size distributions (Methods and Supplementary Figs 9-10). Nanosheet length histograms are displayed in Fig. 3a–c (and Supplementary Fig. 11) for three representative size distributions with mean *L* ranging from 190 to 620 nm. Note that, for much of the remainder of the study, in addition of the std-BP, we have studied dispersions containing small (S-BP, <*L*>=130 nm) and large (L-BP, <*L*>=2.3 μm) to gain insights into size effects (see Supplementary Methods and Supplementary Figs 10 and 12).

We have also analysed the ultraviolet–visible optical response of dispersions of nanosheets of varying size. For each sample, we have measured the optical extinction spectra as shown in Fig. 3d. The absorbance and scattering spectra of the size-selected FL-BP dispersions are also shown in Fig. 3e,f. Very clear spectral changes as a function of size can be seen in all spectra. Such changes are primarily due to the fact that each sample contains a different distribution of nanosheet thicknesses, each of which contributes a significantly different component to the measured spectrum^10^. In addition, the effect of edges may also contribute to the size-dependent changes in spectral shape^33^,^44^. The scattering spectra shown in Fig. 3f also vary strongly with nanosheet length. We have characterized this in more detail in Supplementary Fig. 13, using this data to generate a metric to extract nanosheet length from scattering spectra.

### Photoluminescence

**Figure 4:**
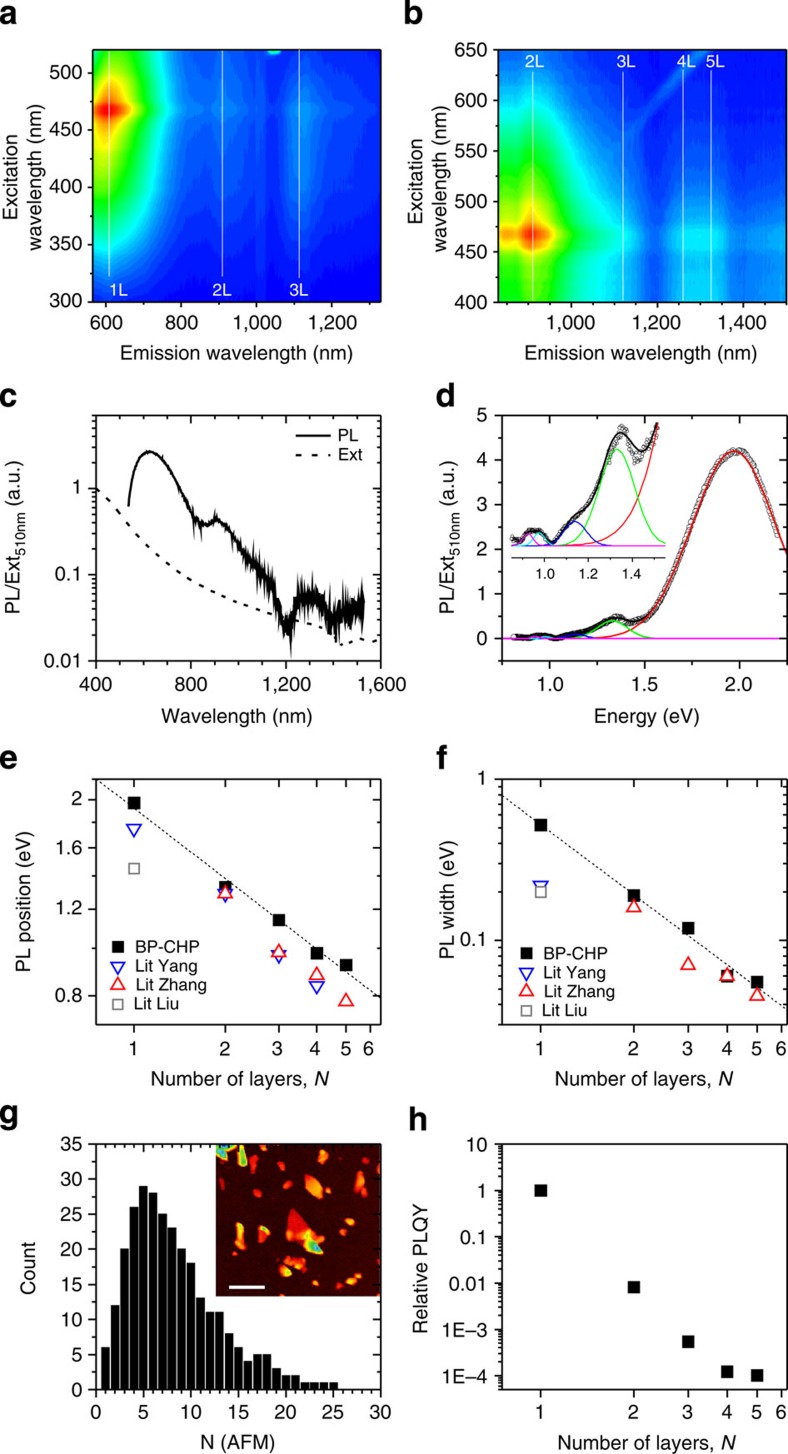
Photoluminescence of dispersed black phosphorous nanosheets. (**a**,**b**) Photoluminescence emission–excitation contour maps measured on a size-selected BP dispersion in CHP exfoliated under inert gas conditions (see Methods) measured with a 550 nm and 830 nm cut-off filter in emission, respectively. (**c**) Photoluminescence line spectrum (wavelength, *λ*_exc_=510 nm) for this BP dispersion in CHP. Also shown is the extinction spectrum for this dispersion as dashed line. (**d**) PL line spectrum, plotted versus photon energy, fitted to five Gaussian lines, representing the PL contributions from 1-, 2-, 3-, 4- and 5-layer nanosheets. (**e**,**f**) Position (**e**) and width (**f**) of fit lines shown in **d**, plotted versus layer number. The dashed lines in **e**,**f** show power law decays with exponents of −0.48 and −1.4, respectively. Also shown in **e**,**f** are data for mechanically cleaved BP nanosheets taken from Yang *et al*.^46^, Zhang *et al*.^13^ and Liu *et al*.^45^ (**g**) AFM image (inset, scale bar 500 nm) and statistical analysis of nanosheet thickness (expressed as layer number, sample size=281) for the samples used to measure PL. (**h**) Relative PL quantum yield (by integrated PL area) as a function of layer number.

A number of publications have demonstrated PL from mechanically exfoliated BP nanosheets with one to five layers^13^,^45^,^46^. To investigate the possibility of PL from liquid-exfoliated FL-BP, we prepared dispersions by performing the sonication under inert gas conditions to avoid any partial oxidation of the BP. However, we note that the sample produced using standard centrifugation was very similar to our std-BP exfoliated under ambient conditions (see Supplementary Figs 14–19 for more information). Prior to the PL measurement, additional centrifugation steps were performed (see Methods) to further remove large and thick nanosheets. Shown in Fig. 4a,b are PL emission–excitation contour maps measured for an FL-BP dispersion in CHP. Very clear emission lines can be seen at ∼600 and ∼900 nm with weaker features appearing at ∼1,150, ∼1,260 and ∼1,325 nm. We associate these features with PL from 1L to 5L BP, respectively. Plotted in Fig. 4c is a PL line spectrum, excited at 510 nm with the measured extinction spectrum for comparison. We have fitted this spectrum to five Gaussian (due to inhomogeneous broadening) lines in Fig. 4d, representing 1-, 2-, 3- 4- and 5-layer nanosheets. In agreement with previous results^13^, we see strong layer-number-dependence of both position and width of the fit components, reflecting the changes in the band structure of exfoliated BP^8^,^13^,^14^. The PL intensity also depends strongly on nanosheet thickness. To separate out the effects of thickness-dependent internal PL quantum yield (PLQY) and the population of nanosheets of a given thickness, we performed AFM analysis on this sample (inset of Fig. 4g and Supplementary Fig. 16). From such images, we measured nanosheet layer number and area (estimated as length × width) for ∼300 nanosheets with the layer-number distribution shown in Fig. 4g. We then calculate the relative PLQY by dividing the integrated PL area for each layer number by the volume fraction of nanosheets of that thickness (approximately equivalent to PL/absorbance for each layer number, Fig. 4h, see Supplementary Table 1). As observed previously^13^, we find the PLQY to be extremely sensitive to nanosheet thickness varying by 4 orders of magnitude from one to five layers. This effect^13^ is due to the thickness-dependent band structure of BP, as the internal PLQY (and width) is governed by the density of states distribution for electrons/holes, which depends on the number of band maxima and valleys.

### Degradation

**Figure 5:**
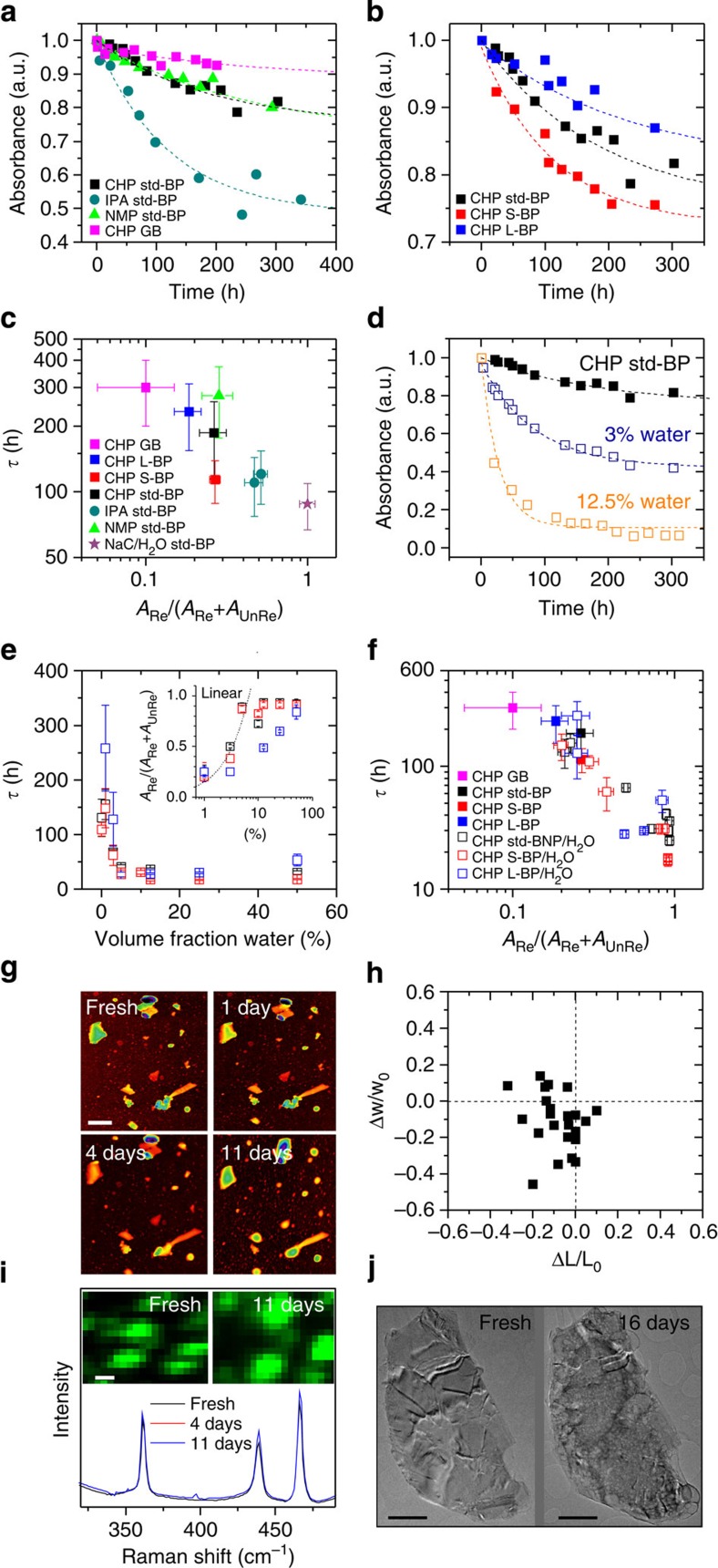
Stability of exfoliated black phosphorous nanosheets. (**a**,**b**) Relative absorbance at 465 nm, measured as a function of time, for (**a**) the standard FL-BP dispersion (std-BP) exfoliated in CHP, NMP and IPA, as well as BP exfoliated in CHP in a glovebox (CHP GB) and (**b**) std-BP in CHP compared with size-selected dispersions containing small (S-BP) and large nanosheets (L-BP). The dashed lines represent exponential decays: *A*=*A*_UnRe_+*A*_Re_*e*^−*t*/*τ*^, where *A*_Re_ represents the component of BP, which reacts with water/O_2_, *A*_UnRe_ represents the unreacted component and *τ* represents the reaction timescale. (**c**) Reaction timescale, *τ*, plotted versus the fraction of BP which reacts, *A*_Re_/*A*_UnRe_+*A*_Re_, for a number of different systems. (**d**) Fitted time-dependent absorbance (465 nm) data for std-BP in CHP and std-BP dispersions with 3 vol% and 12.5 vol% of water added. (**e**) Plot of *τ* versus volume fraction of added water. The inset shows the fraction of unstable BP plotted versus water content. The dashed line demonstrates this fraction to scale initially linearly with water content. (**f**) Plot of *τ* versus *A*_Re_/*A*_UnRe_+*A*_Re_ for BP exfoliated in CHP with and without the addition of water. (**g**) Sequence of AFM images of the same sample region of an as-prepared sample, and after 1, 4 and 11 days of exposure to ambient conditions, respectively (scale bar, 600 nm). Errors in **c**,**e**,**f** are statistical errors associated with the fits. (**h**) Map showing both fractional length, *L*, and width, *w*, changes as measured by AFM immediately after exfoliation after day 4. Negative values of Δ*w*/*w*_0_ and Δ*L*/*L*_0_ indicate that the nanosheets are getting smaller over time. (**i**) Mean Raman spectra (633 nm excitation) summed over the same sample region of an as-prepared sample, after 4 days and 11 days, respectively. Spectra are normalized to the silicon peak at 521 cm^−1^. Inset: Raman A^1^_g_ intensity map of the sample region (freshly prepared and after 11 days). (**j**) TEM images of the same flake deposited from a freshly prepared dispersion and after 16 days.

It has previously been shown that exfoliated BP degrades in the presence of water and oxygen, greatly limiting its application potential^7^,^8^,^12^,^32^. However, this may actually be less of a problem for liquid exfoliation as the solvation shell may protect the nanosheets from reacting with oxygen or water. To test this, we have monitored the temporal stability of liquid-dispersed BP by tracking the absorbance, *A*(*λ*=465 nm), of std-BP, S-BP and L-BP as a function of time (*A*=*αCl*, Fig. 5a, Supplementary Fig. 20 and Supplementary Note 1). As we expect the reaction products to be molecules with wide HOMO-LUMO gaps^32^, the measured absorbance will be dominated by the unreacted BP. This is supported by the lack of spectral changes with time. All samples were shaken before measurement to avoid sedimentation effects and measured at similar absorbances/concentrations. In this case, degradation of the FL-BP should result in a fall in measured absorbance over time, which we do indeed observe for BP exfoliated in a number of solvents (Fig. 5a). It is clear that exfoliation in CHP and NMP results in low reactivity, which can be reduced even further by preparing the dispersions in dried and deoxygenated CHP under inert conditions (sample CHP GB). In addition, we see an increase in the reactivity with decreasing nanosheet size (Fig. 5b, Supplementary Table 2 and Supplementary Fig. 21), suggesting that the chemical reaction starts from the edge as previously found for other 2D materials such as TiS_2_ (ref. 47).

Similar to Martel and co-workers^32^, we fit these curves to exponential decays: *A*=*A*_UnRe_+*A*_Re_*e*^−*t*/*τ*^, where *A*_Re_ represents the total amount of FL-BP which reacts over time and *A*_UnRe_ represents the component which never reacts. Typically we observe time constants of 10s–100s of hours. This is much longer than the decay times of ∼1 h observed by Martel and co-workers^32^ for mechanically cleaved BP in water, implying the degredation kinetics to be considerably different for solvent exfoliated BP. Such fits allow us to parameterize good solvents as those with long *τ* and small *A*_Re_/(*A*_UnRe_+*A*_Re_) (see Supplementary Table 3). In Fig. 5c, we plot *τ* versus *A*_Re_/(*A*_UnRe_+*A*_Re_), extracted from Fig. 5a,b). This plot clearly shows FL-BP exfoliated in dry, deoxygenated CHP to be most stable, followed by NMP and CHP dispersions exfoliated in ambient conditions with ambient IPA dispersions being the least stable among the organic solvents. In addition, we have exfoliated BP in an aqueous surfactant solution with sodium cholate (NaC) as stabilizer (Supplementary Figs 23 and 28). The BP in the aqueous environment degrades most rapidly, although still with considerably longer time constants than micromechanically cleaved BP. This suggests the surfactant shell to offer partial protection, as with carbon nanotubes^48^.

These results cannot solely be explained by residual water content of these solvents (Supplementary Fig. 22) so we suggest that the solvation shell of CHP does indeed protect the nanosheets from water and/or oxygen. This is consistent with recent computational studies which show NMP, CHP and IPA to form tightly packed solvation shells adjacent to BP surfaces. The same study showed the molecular ordering within the solvation shell to vary from solvent to solvent, perhaps explaining the observed solvent-dependent stability^49^.

We note that to stably exfoliate and suspend BP, a solvent must fulfil two criteria: it must have the correct surface energy^50^ to facilitate exfoliation and avoid reaggregation, and it must form a solvation shell which acts as a barrier to oxygen/water. Compared with most 2D materials, where only the first criterion is important, this implies that relatively few solvents will be successful at both exfoliating and protecting BP. Indeed we have only had success using CHP, NMP and to a lesser extent IPA. While others have used DMF, ethanol and other solvents to suspend FL-BP^30^,^31^, the in-solvent stability has generally not been studied making it impossible to assess whether they meet the second criterion. It is clear that more work is needed to expand the list of solvents that can both suspend and protect BP.

However, such protection is clearly not perfect as we see some reactivity in all samples. To characterize this, we have added water (which also contains dissolved O_2_) to dispersions of S-BP, std-BP and L-BP in CHP. Addition of water/O_2_ significantly increases the reactivity (Fig. 5d, see Supplementary Table 4 and Supplementary Fig. 23) with *τ* falling dramatically with added water content (Fig. 5e). In addition, *A*_Re_/(*A*_UnRe_+*A*_Re_) increases linearly with water contents up to ∼10 vol% water, above which it saturates (Fig. 5e inset). Plotting *τ* versus *A*_Re_/(*A*_UnRe_+*A*_Re_) for the water addition samples shows a steady deterioration of stability as water is added (Fig. 5f).

Furthermore, we investigated the degradation of liquid-exfoliated FL-BP after deposition onto substrates using AFM, Raman and TEM. In these experiments, we monitored the same sample region (that is, the same nanosheets) with AFM and Raman immediately after deposition until 11 days later. As shown by the AFM images in Fig. 5g (and Supplementary Figs 24–26), we observe broadening and blurring (that is, loss of fine structure) of the nanosheets. This is typically accompanied with shrinking of nanosheet length, *L* and width, *w* (Fig. 5h, Supplementary Fig. 27). In some cases, we observe an increased apparent height which is typically accompanied with an increased contrast in the phase images (Supplementary Figs 25,26), attributed to the adsorption of water. However, it is important to note, that even after 11 days of ageing under ambient conditions, the CHP-exfoliated FL-BP is clearly still intact, as the average Raman spectra (normalized to Si) of the same sample region show no spectral changes, especially with respect to A_g_^1^/A_g_^2^ (Fig. 5i and Supplementary Fig. 28). These data imply that the overall structure of the FL-BP nanosheets does not change with time, consistent with degradation occurring at nanosheet edges rather than the basal plane. This is further confirmed by XPS (Supplementary Fig. 29) and TEM analysis where we tracked the same nanosheets over time (Fig. 3j and Supplementary Fig. 30). While the flakes look disrupted and, as with AFM, blurred out at the edges, most of the nanosheets remain intact even after 16 days. It is important to emphasize that we clearly observe degradation when using a less favourable, low boiling point solvent such as IPA (Supplementary Fig. 31). Taken together this suggests that solvents such as CHP protect the nanosheets from degrading at the basal plane with the solvation shell remaining even after the bulk solvent has been removed. Importantly, it also shows that degradation starts at the edges. This implies that the structure of the solvation shell is different at the nanosheet edges in a way that makes water/O_2_ ingress at the edges much more likely than at the basal plane. This preferential edge degradation has important implications for future applications of BP. Edge degradation leaves the basal plane intact and should not greatly affect the properties of the BP nanosheets other than their size. This makes the environmental instability of BP less problematic for applications than might previously have been thought.

### Reaction mechanism

**Figure 6:**
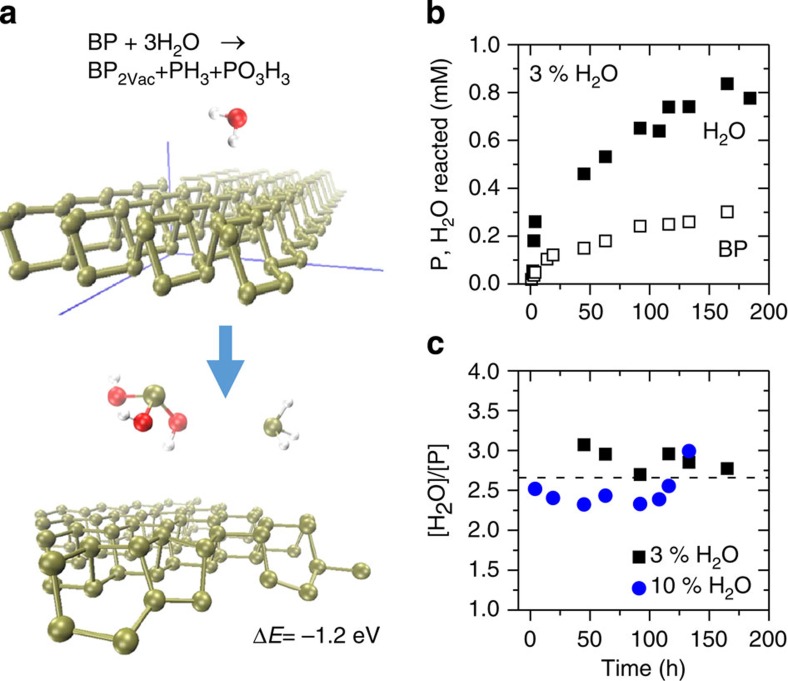
Reactivity of solvent-stabilized Black Phosphorous with water. (**a**) Edge selective degradation model for BP exposed to pure neutral water. Top and bottom panel represent reagent (BP edge+three water molecules) and reaction products (BP defective edge+phosphine+phosphorous acid), respectively, with the reaction energy also given. Green, red and white balls represent P, O and H atoms, respectively. (**b**) Experimental data for amount of reacted phosphorus and water, respectively, as a function of time for std-BP GB in CHP after addition of 3 vol% of water. The data was obtained from tracking both water and BP concentrations by ultraviolet–visible spectroscopy (see Supplementary Note 3). (**c**) Molar ratio of water/BP as a function of time measured for a std-BP GB dispersion in CHP after addition of 3 and 10 vol% of water. The molar ratio is centred at 2–3, which is reasonably consistent with the proposed edge degradation reaction.

If the basal plane is protected by solvent such that the dominant reaction pathway in liquid-exfoliated nanosheets occurs at the edges, then the reaction mechanism may differ to that proposed by Martel and co-workers^32^ Here we suggest an alternative pathway involving nanosheet edges, which is supported by density functional theory calculations (see also Supplementary Note 2). Since we experimentally observe a drop in pH with time after water addition and the formation of phosphorous and/or phosphoric acid (XPS), we propose the following acid–base disproportionation reaction involving nanosheet edges (Fig. 6a), which may become dominant when basal plane reactions are suppressed:


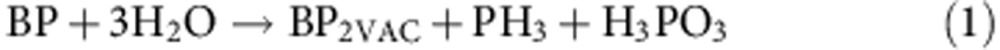


This reaction was evaluated for both edge sites and basal plane P atoms. In both cases, a defective structure BP_2VAC_, with two P vacancies in the BP supercell is formed (2.5% vacancy concentration, Supplementary Figs 32 and 33). The reaction energy of process (1) is evaluated as


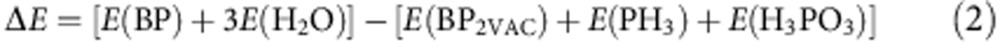


where [*E*(BP)+3*E*(H_2_O)] is the energy of the nanosheet and three isolated water molecules at infinite distance from the nanosheet and [*E*(BP_2VAC_)+*E*(PH_3_)+*E*(H_3_PO_3_)] is the energy of the defective nanosheet infinitely distant from the other isolated reaction products. This reaction is exothermic (Δ*E*=−1.2 eV, see Fig. 6) when the process occurs at the edge but endothermic (Δ*E*=0.26 eV) when occurring far from the edge (see Supplementary Figs 34 and 35). In either case, degradation is a multistep process. Since we are interested in whether a reaction starts at the edge or the basal plane, we have analysed the early steps in the reaction (approach of H_2_O and splitting of H_2_O to hydroxyl group and H atoms chemisorbed to neighbouring P). In both edge and basal plane cases, the water adsorption is slightly exothermic, while the splitting of the water is highly unfavourable on the basal plane (Supplementary Fig. 34). We have furthermore ruled out that the disproportionation reaction could proceed in the middle of BP nanosheet if activated by the formation of the first hole by studying the degradation process starting from a defective structure BP_2VAC_ (Supplementary Fig. 36). The reaction is again endothermic by ∼Δ*E*=0.26 eV further supporting that the reaction is unlikely to occur on the basal plane.

These calculations show that BP can react with water, even in the absence of oxygen, to remove P atoms from the nanosheet edge although this reaction may only be important when basal plane reactions^32^ are suppressed. To test the validity of the proposed reaction, we attempted to experimentally determine the stoichiometric ratio of reacted BP and water. After addition of water to a BP dispersion, the consumption of both water and BP can be tracked by ultraviolet–visible spectroscopy (see Supplementary Figs 37 and 38, Supplementary Note 3). Knowing the extinction coefficients of both components, this data can be converted to the concentration of reacted BP and water, respectively (Supplementary Fig. 37). The reacted concentration as a function of time for both BP and water is shown in Fig. 6b (using the CHP GB dispersion after addition of 3 vol% of water). It is clear that two to three water molecules are consumed for every P atom. As shown in Fig. 6c, a similar stoichiometry ratio is obtained when analysing a dispersion where initially 10 vol% of water was added. The thus experimentally determined H_2_O/P ratio of two to three agrees well with the proposed edge reaction. However, it can unfortunately not distinguish between the proposed edge disproportionation reaction and the reported basal plane oxidation^32^.

### Applications

For liquid-exfoliated FL-BP, the degradation timescale is slow enough to allow processing of nanosheets for applications testing in a number of areas. We believe that if applications potential is demonstrated, practical usage of FL-BP will be enabled by encapsulation^7^,^24^. In this work we have chosen three applications, which have not previously been described to demonstrate the broad potential of FL-BP.

**Figure 7:**
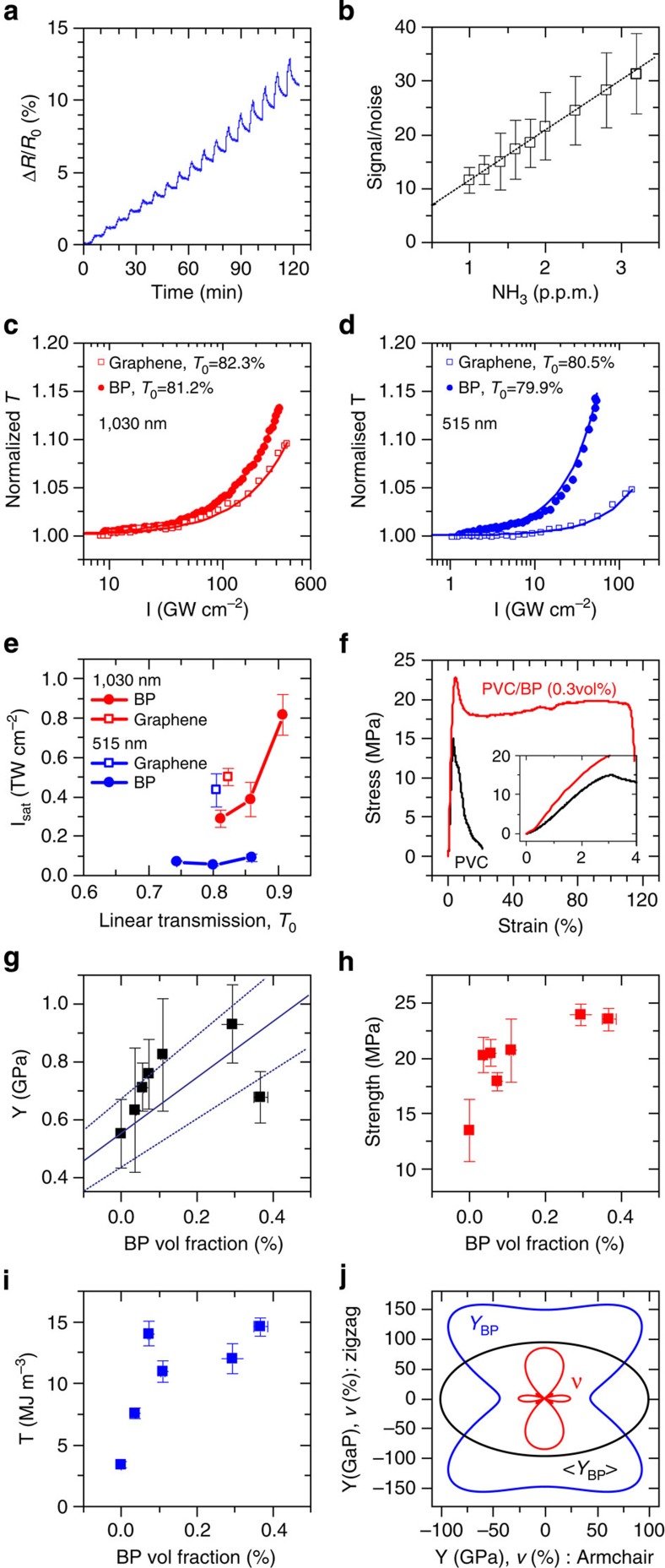
Applications of liquid-exfoliated FL-BP. (**a**,**b**) Sensing of NH_3_ gas using std-BP films. (**a**) Sensor response plot shows percentile resistance change versus time of the FL-BP films with a bias voltage of 1 V at room temperature, on consequent NH_3_ exposures at various concentrations from 1 to 10 p.p.m. (**b**) Plot of signal-to-noise ratio as a function of NH_3_ concentration from 1 to 3 p.p.m. The error bar represents the s.d. of five devices and the linear line indicates the fitted line. (**c**–**e**) Saturable absorption of std-BP and graphene in CHP for fematosecond pulses excited at (**c**) 1,030 nm and (**d**) 515 nm. Linear transmission *T*_*0*_ is given in the legend. (**e**) Saturation intensity of FL-BP and graphene as a function of *T*_*0*_. (**f**) Representative stress–strain curves for PVC and PVC: FL-BP (0.3 vol%). Inset: low strain regime. (**g**–**i**) Young's modulus, including the theoretical constant-strain rule-of-mixtures modulus prediction (blue using the Voigt–Reuss–Hill planar-averaged nanosheet modulus, blue-dashed using its Voigt and Reuss bounds) (**g**), tensile strength (**h**) and tensile toughness (**i**) plotted as a function of FL-BP volume fraction. (**j**) Calculated orientation dependence of the in-plane two-dimensional Young's modulus (*Y*_BP_, blue) and Poisson's ratio (*ν*, red), of black phosphorus, together with the Voigt–Reuss–Hill averaged in-plane modulus (<*Y*_BP_>, black). Errors in **b**,**e**,**g**–**i** are statistical errors associated with the fits.

Theoretical work has suggested FL-BP nanosheets as gas sensors^18^. We prepared thin films of FL-BL nanosheets by vacuum filtration followed by transfer to interdigitated electrode arrays. Two-probe measurements showed relatively high conductivities of ∼1 S m^−1^, similar to films of WTe_2_ nanosheets^51^. Shown in Fig. 7a (and Supplementary Fig. 39) is the resistance change of a thin film of FL-BP nanosheets on exposure to ammonia (NH_3_) gas. We observe a resistance increase, consistent with NH_3_ donating electrons to the p-type FL-BP. By extrapolation of the signal-to-noise level (Fig. 7b), and assuming the minimum detectable signal to be three times the root mean square noise level, we estimate a detection threshold of 80 p.p.b. This shows FL-BP networks to be competitive with other nano-sensors^53^,^54^,^55^ and a very promising material for gas detection.

The nonlinear optical response of the FL-BP dispersions was investigated by open-aperture Z-scan using a 340-fs pulsed fibre laser^55^. In Fig. 7c,d, it is clearly seen that normalized transmission of the FL-BP dispersions increases with laser intensity at both 1,030 and 515 nm. Such broadband saturable absorption (SA) suggests that the FL-BP nanosheets could serve as an ultrafast nonlinear saturable absorber, an essential mode-locking element for ultrashort pulsed lasers^56^,^57^. Since graphene is a well-known broadband saturable absorber^58^, we carried out the same nonlinear measurement for graphene dispersions prepared by the similar liquid exfoliation. At the same level of linear transmission, the FL-BP dispersions exhibit much stronger SA response than the graphene dispersions at both wavelengths. The saturable intensity *I*_s_ is obtained by fitting the Z-scan data with the SA model d*I*/d*z*=−*αI*, where *α*=*α*_0_/(1+*I*/*I*_Sat_): *α*_0_ is the linear absorption coefficient and *I* is the excitation intensity. As shown in Fig. 7e, *I*_Sat_ of FL-BP is much lower than that of graphene at both 1,030 and 515 nm when the linear transmission is equal. The significant ultrafast nonlinear property of FL-BP implies a huge potential in the development of nanophotonic devices, such as mode-lockers, Q-switchers, optical switches and so on^59^.

The impressive mechanical properties of 2D materials in general^60^ suggest the potential to use FL-BP as a reinforcing filler in composites. Shown in Fig. 7f are the representative stress–strain curves for a film of polyvinylchloride (PVC) and a PVC:FL-BP (0.3 vol%, see methods and Supplementary Figs 40 and 41). It is clear that the mechanical properties improve considerably both in the high and low strain regimes. Shown in Fig. 7g-i are the composite modulus, strength and tensile toughness, plotted as a function of BP loading content. In each case, mechanical properties increase considerably for loading levels of only 0.3 vol%. The modulus, *Y*, increases from 500 MPa for PVC to 900 MPa for the 0.3 vol% composite. In addition, at 0.3-vol% loading, the measured strength of the composite doubles while its tensile toughness displays a sixfold increase. These are significant increases and are competitive with those found using graphene as a filler in both PVC^61^ and polymers in general^62^.

We used first-principles simulations (see Supplementary Note 4) to obtain the bulk BP Young's modulus as a function of orientation in the plane of the crystal as shown in Fig. 7j, yielding a Voigt–Reuss–Hill in-plane average modulus of 〈*Y*_BP_〉=97 GPa. We also calculated the Poisson's ratio finding it to be highly anisotropic. Assuming the BP nanosheets lie in-plane^63^, we can use the calculated 〈*Y*_BP_〉, coupled with the rule of mixtures^62^ to predict the composite modulus as a function of BP volume fraction, *V*_f_: *Y*_comp_=〈*Y*_BP_〉*V*_f_+*Y*_poly_(1−*V*_f_). We find this prediction (blue line) to agree well with the experimental data even though we have not corrected for the finite aspect ratio of the nanosheets or their layered nature.

## Discussion

In conclusion, we have shown that the BP crystals can be efficiently exfoliated in appropriate solvents to yield high-quality, few-layered nanosheets with controllable size, which can be used for fundamental measurements such as PL studies. Compared with mechanically cleaved nanosheets, liquid-exfoliated FL-BP is remarkably stable in CHP, probably due to protection by the solvation shell. However, addition of water results in degradation of the nanosheets. Both experimental and computational studies indicate the degradation to occur at the nanosheet edge with no basal plane damage and proceed by reaction of the FL-BP with water. Based on this, we propose an alternative BP degradation reaction, which can occur when basal plane reactions are supressed. Importantly, this means that the net result of degradation is reduction on nanosheet size rather than alteration of flake properties. We demonstrate that liquid-exfoliated FL-BP nanosheets have potential for use in applications as gas sensors, saturable absorbers and reinforcing fillers for composites. We believe this work is important as it will facilitate the large-scale production of FL-BP. Such a goal is realizable in practise because, even when produced in ambient conditions, CHP-exfoliated FL-BP is stable for ∼200 h (*cf*, ∼1 h for mechanically cleaved BP). We believe these advances will facilitate its development in a broad range of applications.

## Methods

### Sample preparation

BP crystals were purchased from Smart Elements (purity 99.998%) with all other materials sourced from Sigma Aldrich (all used as received). BP was lightly ground with pestle and mortar and immersed in CHP (concentration 2 g l^−1^). The dispersion was sonicated for 5 h at 60% amplitude with a horn-probe sonic tip (VibraCell CVX, 750W) under cooling, yielding a stock dispersion. Aliquots of the stock dispersion were centrifuged at 1,000 r.p.m. (106*g*) for time periods varying from 5 to 240 min in a Hettich Mikro 220R centrifuge equipped with a fixed-angle rotor 1016. The supernatant was decanted and subjected to absorbance spectroscopy. The supernatant with centrifugation conditions 1,000 r.p.m. for 180 min was denoted std-BP. The std-BP was subsequently separated into small and large stable nanosheets. For this purpose, aliquots of std-BP dispersion were subjected to an additional centrifugation of 5 kr.p.m. (2,660*g*) for 120 min. The supernatant (containing small flakes) was decanted and characterized as S-BP, while the sediment (containing large flakes) was redispersed in fresh CHP and characterized as L-BP. Alternatively, the FL-BP was size-selected by controlled centrifugation with subsequently increasing rotation speeds. The sediment after 2 kr.p.m. (426*g*, 2 h) was discarded, while the supernatant was subjected to further centrifugation at 3 kr.p.m. (958*g*, 2 h). The sediment was collected in fresh solvent, while the supernatant was subjected to further centrifugation at 4 kr.p.m. (1,702*g*, 2 h). Again, the sediment was collected and the supernatant was centrifuged at high r.p.m. This procedure was repeated for 5 kr.p.m. (2,660*g*, 2 h), 10 kr.p.m. (10,170*g*, 2 h) and 16 kr.p.m. (25,000*g*, 2 h) to yield samples with decreasing sizes in the respective sediments. For part of the degradation studies and PL measurement, BP was exfoliated under inert conditions by sonication in an argon-filled glovebox (O_2_<0.1 p.p.m.; H_2_O<0.1 p.p.m.) in pump-freeze deoxygenated and dry CHP with a water content of 29 p.p.m. (0.5 g l^−1^ BP, 15 ml CHP, tapered tip Bandelin Sonoplus 3100, 25% amplitude, 2 h, pulse 2 s on, 2 s off). The resultant dispersions were transferred into centrifugation vials, which were sealed and centrifuged for 3 h at 100*g* to obtain the std-BP GB sample. To increase the population of few-layered species for the PL measurement, this std-BP GB sample was centrifuged for 16 h at 25*g*. The sediment was discarded and the supernatant centrifuged again at 710*g* for 180 min. The PL spectra shown in the main manuscript were acquired from the supernatant of this centrifugation after dilution to an optical density of 0.4 cm^−1^. All solvent transfer was carried out in the glovebox.

### Characterization

Optical extinction and absorbance were measured on a Perkin Elmer 650 spectrometer in quartz cuvettes. To distinguish between contributions from scattering and absorbance to the extinction spectra, dispersions were measured in an integrating sphere using a home-built sample holder to place the cuvette in the centre of the sphere. The absorbance spectrum is obtained from the measurement inside the sphere. A second measurement on each dispersion was performed outside the sphere to obtain the extinction spectrum. This allows for the calculation of the scattering spectrum (extinction absorbance). The experiments to track both water and BP degradations were performed on a Perkin Elmer Lambda 1050 spectrometer in extinction.

Bright-field TEM imaging was performed using a JEOL 2100, operated at 200 kV, while HRTEM was conducted on a FEI Titan TEM (300 kV). High resolution TEM images (Fig. 5f) were taken using an FEI Titan 60–300 Ultimate Microscope operated at 300 kV. The FL-BP was dropped on grids using a drop casting method and excess fluid was absorbed by an underlying filter membrane. It was then baked in vacuum at 120 °C for several hours. The samples were imaged on the day they were received which is termed Day 1. The same flake was then imaged on Day 3 and 16. No changes in nanosheet structure of morphology were observed between Day 1 and Day 3, but by Day 16 a combination of reaction products and water adsorption results in a liquid layer on the flake. This layer can be removed with the beam, and comparison of the shape of the flakes between Day 1 and Day 16 shows that the overall shape and size of the flake has not changed. There is also lattice apparent in the flake on Day 16 when using a high magnification ( × 300k).

Aberration-corrected STEM images were taken using a Nion Ultrasteme 100 (cold filed emission gun at the SuperSTEM Laboratory in Daresbury, UK. The suspended FL-BP flakes were dropped onto lacey carbon-coated copper TEM grids as before. The samples were then prebaked at 120 °C in a vacuum overnight. The images were recorded using a 100 kV acceleration voltage with a HAADF field detector and low-pass bright-field imaging. Figure 1f shows a representative Butterworth-filtered HAADF STEM image and Figure 1g shows a low-by-pass bright-field STEM image of the material. The lattice shows high uniformity with the presence of very few defects or dislocations. Depending on the zone axis used, the lattice can exhibit a ‘dumbbell'-type configuration.

AFM was carried out on a Veeco Nanoscope-IIIa (Digital Instruments) system equipped with an E-head (13 μm scanner) in tapping mode after depositing a drop of the dispersion transferred to IPA on a pre-heated (150 °C) Si/SiO_2_ wafer with an oxide layer of 300 nm. Raman spectroscopy on individual flakes was performed using a Horiba Jobin Yvon LabRAM HR800 with 633 nm excitation laser in air under ambient conditions. XPS was performed under ultra-high vacuum conditions (<5 × 10^−10^ mbar), using monochromated Al Kα X-rays (1,486.6 eV) from an Omicron XM1000 MkII X-ray source and an Omicron EA125 energy analyser. An Omicron CN10 electron flood gun was used for charge compensation and the binding energy scale was referenced to the adventitious carbon 1s core level at 284.8 eV. Core-level regions were recorded at an analyser pass energy of 15 eV and with slit widths of 6 mm (entry) and 3 mm × 10 mm (exit), resulting in an instrumental resolution of 0.48 eV. After subtraction of a Shirley background, the core-level spectra were fitted with Gaussian–Lorentzian line shapes using the Marquardt's algorithm.

PL in dispersion was acquired on a Horiba Scientific Fluorolog-3 system equipped with 450 W Xe halogen lamp and a nitrogen-cooled InGaS diode array detector (Symphony iHR 320). Spectra were obtained at 5 °C using appropriate cutoff filters (see Supplementary Figs 17–19).

### Modelling

The QuantumEspresso package was used to evaluate the reaction energies. In this case, we selected an energy cutoff of 50 Ryd, a grid of 2 × 2 × 1 Monchorst–Pack k-points (see Supplementary Note 2) and ultrasoft pseudopotentials. Langreth and Lundqvist van der Waals-corrected exchange and correlation functional^64^ was used and atomic forces were relaxed until they were <5 × 10^−3^ eV A^−1^.

We also calculated bulk and in-plane mechanical properties of BP (see Supplementary Note 4 and Supplementary Tables 5–7) to clarify the microscopic origins of an observed dramatic increase in the Young's modulus of composites reinforced with BP nanosheets. In this case, the PBE functional, augmented with the empirical dispersion correction of the B97-D functional^65^ was used to compute the elastic constants of bulk phosphorus by finite-difference, the nanosheets in question being closer to the bulk than monolayer regime. The Voigt–Reuss–Hill approach,^66^ was used to estimate both isotropic (found to be comparable to current literature^67^) and nanosheet in-plane-only estimates of the average nanosheet Young's modulus, as well as upper (Voigt) and lower (Reuss) expected error bounds.

### Applications

For nonlinear optical measurements, an open-aperture Z-scan system was used to study the ultrafast nonlinear optical properties of the FL-BP (std-BP) and graphene dispersions. This measures the total transmittance through a sample as a function of incident laser intensity, while the sample is sequentially moved through the focus of a lens (along the *z*-axis)^55^,^57^. All experiments were performed with 340-fs pulses from a mode-locked fibre laser, which was operated at 1,030 nm and its second harmonic, 515 nm, with a pulse repetition rate of 1 kHz. All dispersion samples were tested in quartz cuvettes with 1-mm pathlength.

Gas sensing was conducted on the FL-BP (std-BP) prepared in CHP and then subsequently transferred into 2-proponal by centrifugation as conducted for the AFM measurements to facilitate filtration onto a nitrocellulose membrane. Following filtration, the film was allowed to dry under vacuum conditions. The FL-BP film was cut into 10 × 2-mm rectangular pieces. These were then transferred onto silicon dioxide wafer, while the nitrocellulose membrane was dissolved using the transfer method according to the study by Wu *et al*.^68^

For gas sensing, gold electrodes were sputtered on top of an adhesion layer of nickel (Ni/Au=30/70 nm) using a metal shadow mask, which has a 2-mm-wide and 200-μm-long channel. All devices were loaded in a gas-sensing chamber and annealed at 100 °C for 1 h to remove residues and adsorbates on the surface. The gas-sensing chamber was kept at room temperature at a pressure of 10 Torr, with a 100 s.c.c.m. flow of the NH_3_ and N_2_ mixtures. The resistance change of five devices on interval gas exposure was simultaneously measured using a Keithley model 2612A SourceMeter and a Keithley 3706 System Switch at a constant bias voltage of 1 V. The initial resistance and root mean square noise were calculated from the first 500 data points, ∼2 min before the first gas injection. NH_3_ for 2 min and pure N_2_ for 5 min were periodically introduced to record sensor response and recover, respectively.

A previously prepared nanofiller FL-BP dispersion in CHP, centrifuged between 1 kr.p.m. for 180 min and 3 kr.p.m. for 120 min and redispersed in fresh CHP solvent, was subsequently filtered onto a Polyester Membrane Filter (0.2 μm) of known mass. The membrane was dried in a vacuum oven at 100 °C for 2 h and the mass of the membrane was remeasured to attain the mass of the filtered nanofiller. The nanofiller was redispersed by bath sonication (Branson 1510 Model 45 kHz) in a 65:35 tetrahydrofuran (THF), chloroform solvent mixture. PVC was dissolved in a solvent mixture (65:35 THF/chloroform). A range of FL-BP/PVC/THF/Chloroform dispersions (from 0 to 0.0074 volume fraction) were made by adding the FL-BP/THF/Chloroform filler solution to the PVC/THF/Chloroform solution with varying increments of loading. These solutions were of constant mass (150 mg of FL-BP and polymer) and constant volume (5 ml of FL-BP, polymer and solvent). These samples were sonicated in the same bath as before for 1 h to homogenize after the blending of the solutions. The homogenized solution mixtures were then dropcast into 5 × 5 × 1 cm Teflon trays and placed in a vacuum oven for 4 h at 40 °C under no vacuum to form composite films. The films were then kept overnight (∼17 h) at 50 °C under full vacuum to ensure that the solvent was completely removed and to protect the filler material from decomposition before testing. For mechanical measurements, the films were cut into 2.25-mm strips and then tested on a Zwick Roell tensile tester with a 100 N load cell at a strain rate of 10 mm min^−1^.

## Additional information

**How to cite this article:** Hanlon, D. *et al*. Liquid exfoliation of solvent-stabilized few-layer black phosphorus for applications beyond electronics. *Nat. Commun.* 6:8563 doi: 10.1038/ncomms9563 (2015).

## Supplementary Material

Supplementary Information Supplementary Figures 1-41, Supplementary Tables 1-4, Supplementary Notes 1-4, Supplementary Methods and Supplementary References.
