# Supplementary material for: Liquid exfoliation of solvent-stabilized few-layer black phosphorus for applications beyond electronics
Source: Nat Commun. 2015 Oct 15;6:8563. doi: 10.1038/ncomms9563 (PMC4634220; doi:10.1038/ncomms9563)
Supplement: Supplementary Information — Supplementary Figures 1-41, Supplementary Tables 1-4, Supplementary Notes 1-4, Supplementary Methods and Supplementary References. [file ncomms9563-s1.pdf]

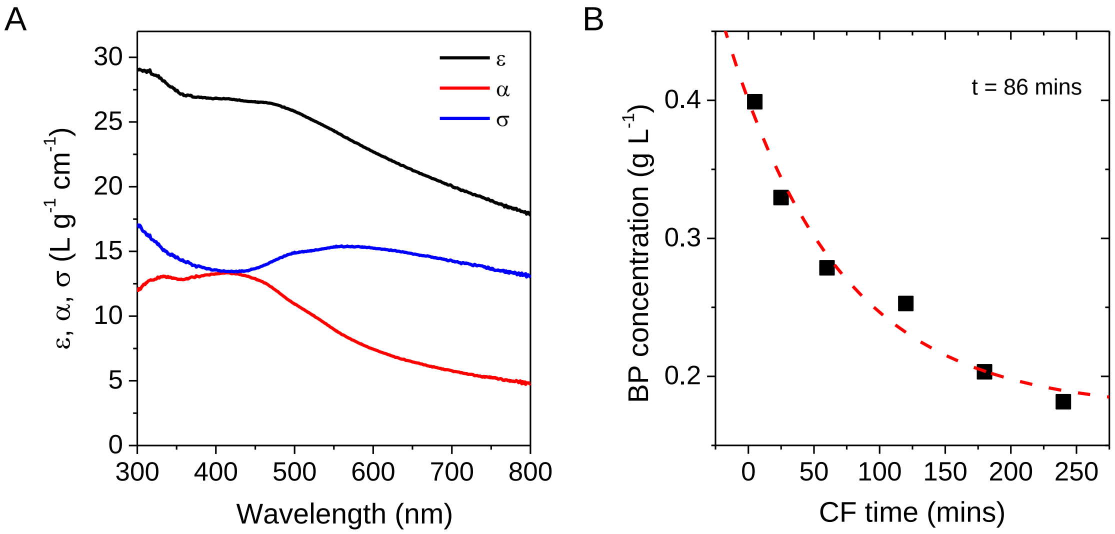

Supplementary Figure 1 | Optimisation of centrifugation conditions. A) Absorbance, extinction and scattering coefficient spectra of the std- BP dispersion in CHP ($C_{initial}$ (BP) = 2 g/L, centrifugation 1 krpm, 180 min). The extinction coefficient ε and absorbance coefficient α at 465 nm was determined as 26.5 L $g^{-1}cm^{-1}$ and 15 L $g^{-1}cm^{-1}$, respectively by filtration and weighing. We then use the absorbance spectra and the related absorbance coefficient to determine the FL-BP concentration (Lambert-Beer law) in the supernatant after centrifugation at different times. B) Concentration as a function of centrifugation time at 1 krpm (106 g). The data follows an exponential decay with a time constant (τ) of 86 min. Therefore to obtain a dispersion where the nanosheets are stable we centrifuge for $t > 2\tau$. This methodology has been demonstrated for previous systems.[1] We note that the absorbance spectra, if available, give a better measure for concentration than extinction spectra as size dependences from scattering (especially for uncentrifuged stock dispersions) are avoided.[2]

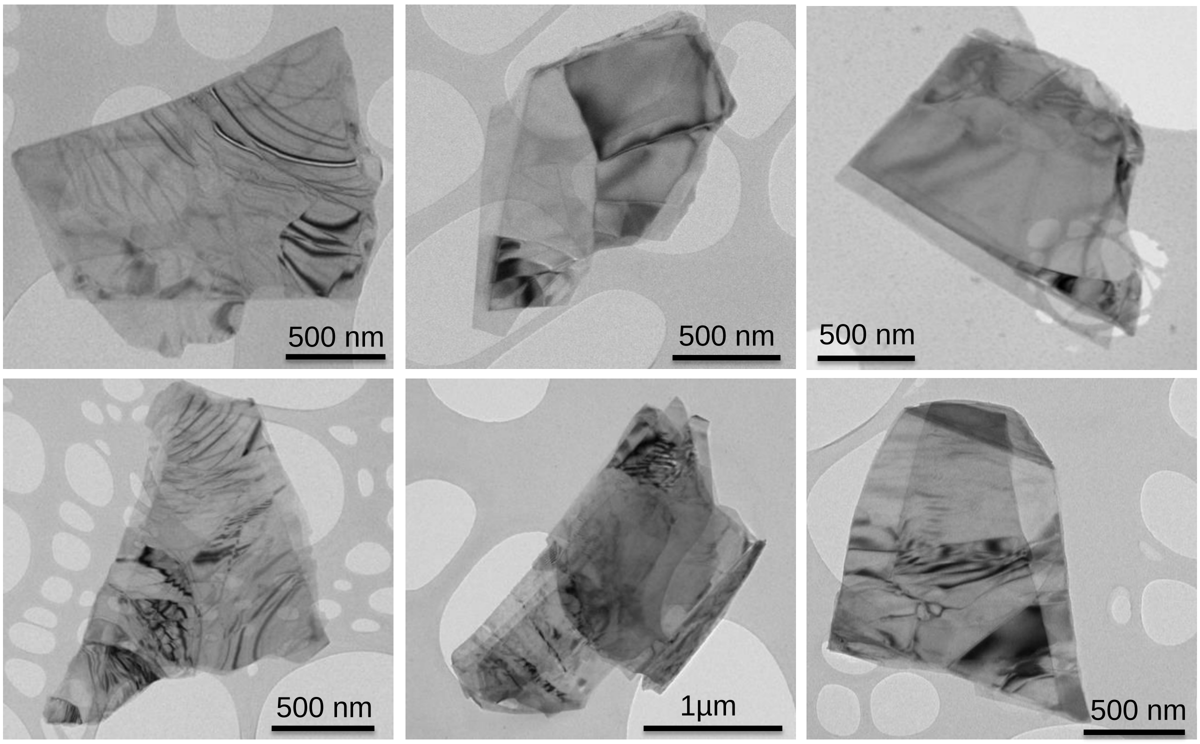

Supplementary Figure 2 | Additional low resolution TEM images of std-BP in CHP. Shown above are six examples of TEM images of BP nanosheets collected in this study.

.

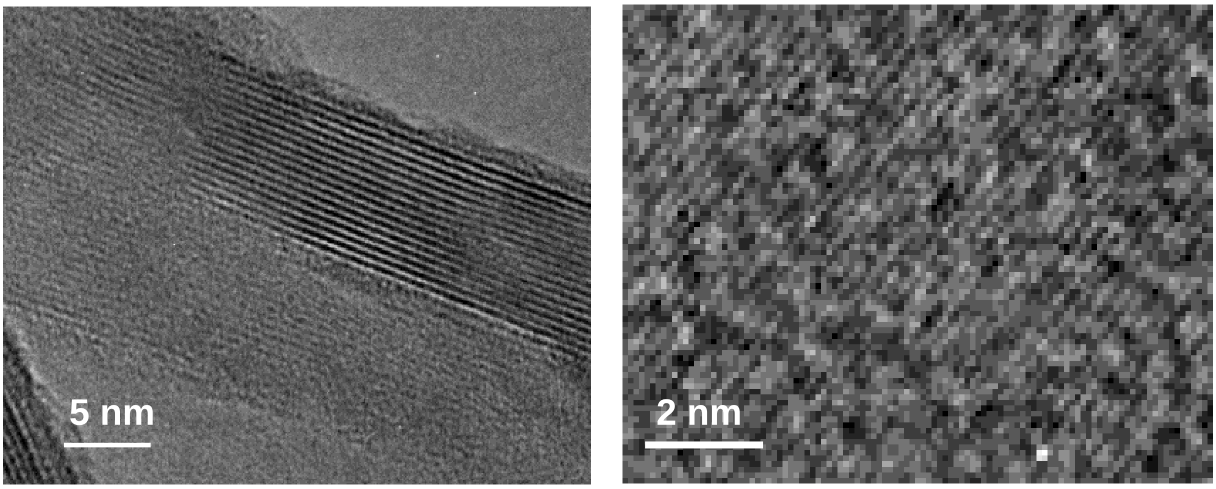

Supplementary Figure 3 | High resolution TEM images of std-BP in CHP. As discussed in the main manuscript, CHP is a highly suitable solvent for the dispersion and exfoliation of BP because it i) yields reasonably thin nanosheets and ii) partly protects the nanosheets from degradation. However, it causes some difficulties when imaging the nanosheets in HRTEM. We attribute this to the high boiling point (284°C) making it difficult to remove the solvent completely from the nanosheets leaving a carbonaceous coverage on the nanosheets (which is still efficiently suppressing the basal plane degradation, see below). This makes it difficult to resolve the lattice structure as shown in the figure. For the STEM analysis presented in figure 1G-H in the main manuscript, we have therefore turned to FL-BP exfoliated in IPA using equal processing parameters (sonication and centrifugation time and speed), even though the nanosheets are significantly less stable (see below).

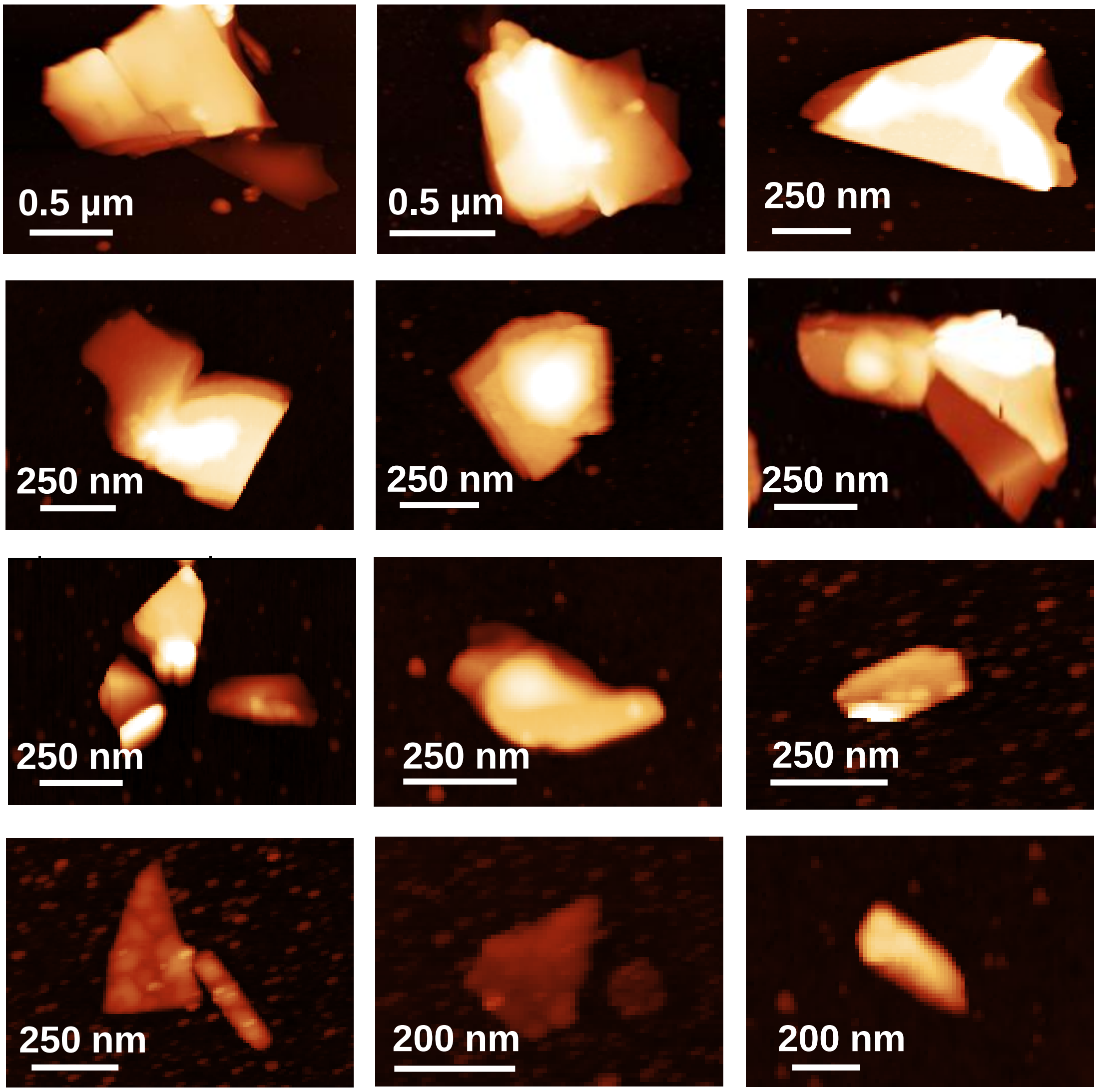

Supplementary Figure 4 | Representative atomic force microscopic (AFM) images of std-BP in CHP after transfer to IPA. We note that it is very challenging to deposit nanosheets from high boiling point solvents CHP. This is because the slow solvent evaporation causes aggregation to occur making statistical analysis impossible, where only individual, non-aggregated flakes are taken into account. Accordingly, the FL-BP was transferred from CHP to IPA by a high speed centrifugation step (22,640 g). The faint yellow, almost clear CHP supernatant was decanted and the sediment reagitated (by 5 min bath sonication) in IPA prior to drop-casting the dispersion on pre-heated (150 °C) Si/SiO_2 wafers. Due to the rather quick solvent evaporation, we found aggregation to be minimal over wide regions.

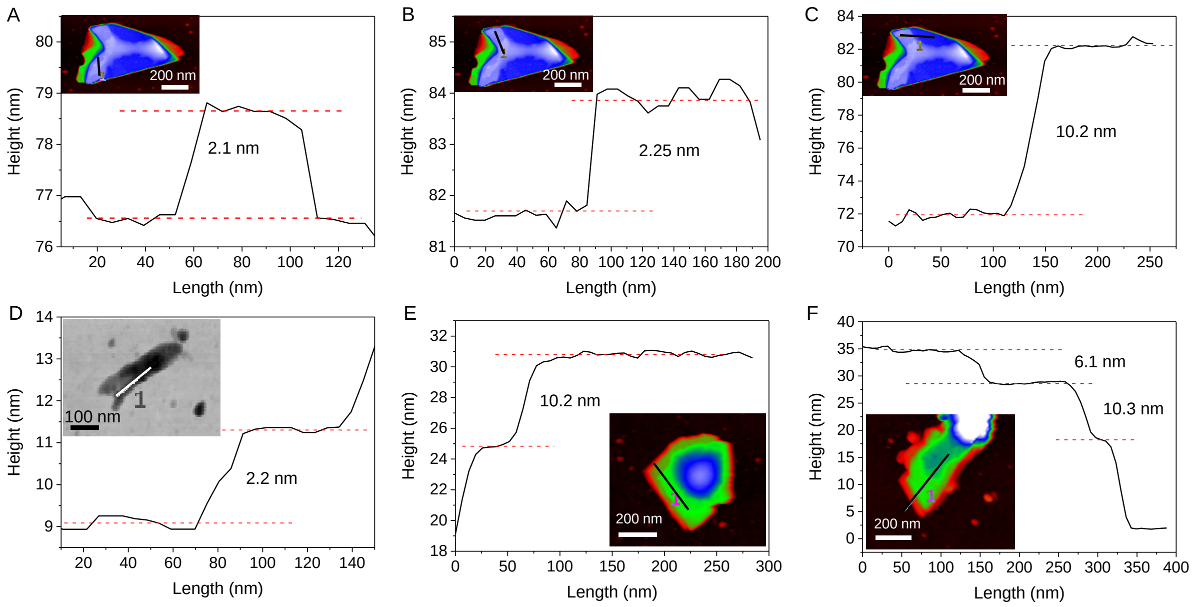

Supplementary Figure 5 | Height profiles across BP nanosheets with steps. The profile was taken along the lines shown on the corresponding nanosheets in the inset. The dashed red lines indicate where the heights for the step height analysis were taken. The related step heights in nm are also shown. The error in assessing the height profile is ~ 0.3 nm. In total, 70 height profiles were examined and the step heights were plotted in ascending order in figure 2E. The apparent height of each step is always a multiple of a discrete value which represents the apparent height of one BP monolayer. To determine the height of one monolayer and the associated error, the as determined values were grouped in 1-7 layers (with step heights of ~ 2, 4, 6, 8, 10, 12, 14 nm) and the mean height of the individual data points in each group is plotted in figure 2E as inset. The error in the mean step group height is taken as the sum of the average step height error and the standard deviation step heights in each group. The apparent monolayer height is determined by plotting the mean step group height in ascending order and finding the slope including the statistical error. The apparent height of one LPE BP monolayer was then determined as 2.06 ± 0.2 nm. Hence, to convert the measured apparent AFM height to number of layers, the measured height was divided by 2.06 nm. Currently, it is unclear why the apparent height of liquid exfoliated nanosheets is overestimated in AFM. However, we note that we have also observed this for LPE graphene,[3] MoS_2[2] and GaS.[4] In the case of graphene and MoS_2, we were able to verify the step heights and number of layers by Raman or Raman/PL spectroscopy on individual nanosheets. This is probably a result of residual solvent on top and below nanosheets, as well as potentially trapped between the layers widening the interlayer distance. In addition, accurate height measurements on inhomogeneous samples (such as nanosheets partially covered with solvent) using AFM

tapping mode are generally challenging, as capillary forces and adhesion depend strongly on the material and scanning parameters.[5, 6]

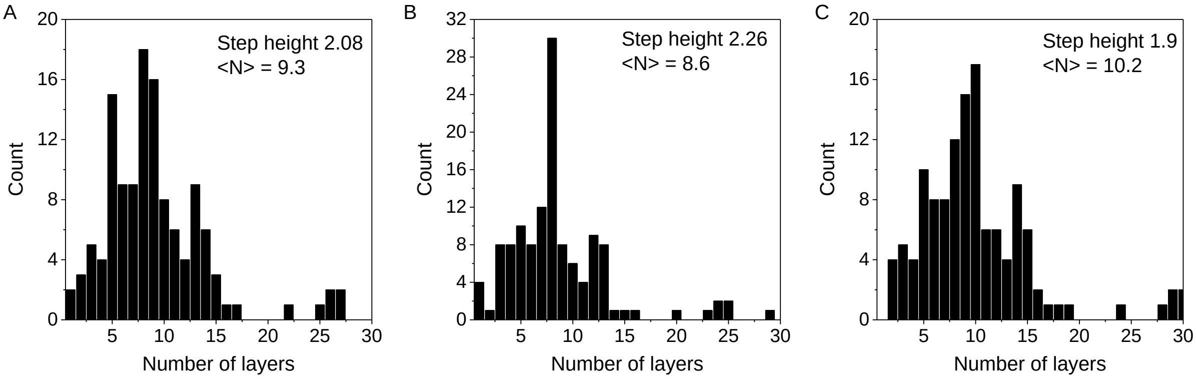

Supplementary Figure 6 | Impact of step height uncertainty on mean number of layers of BP. Number of layer histograms of the std-BP sample obtained from dividing the measured AFM height by the mean of the monolayer height and the upper and lower boundaries of the error. A) Histogram assuming a monolayer height of 2.06 nm, B) Histogram assuming a monolayer height of 2.06 + 0.18 nm, C) Histogram assuming a monolayer height of 2.06 - 0.18 nm. The mean number of layers <N> is indicated in the panels and varies between 8.6-10.2 giving a contribution to the error in <N> of 0.8. In each histogram, the standard error of the mean is 0.5. This yields a mean number of layers of the std sample of 9.4 ± 1.3 nm.

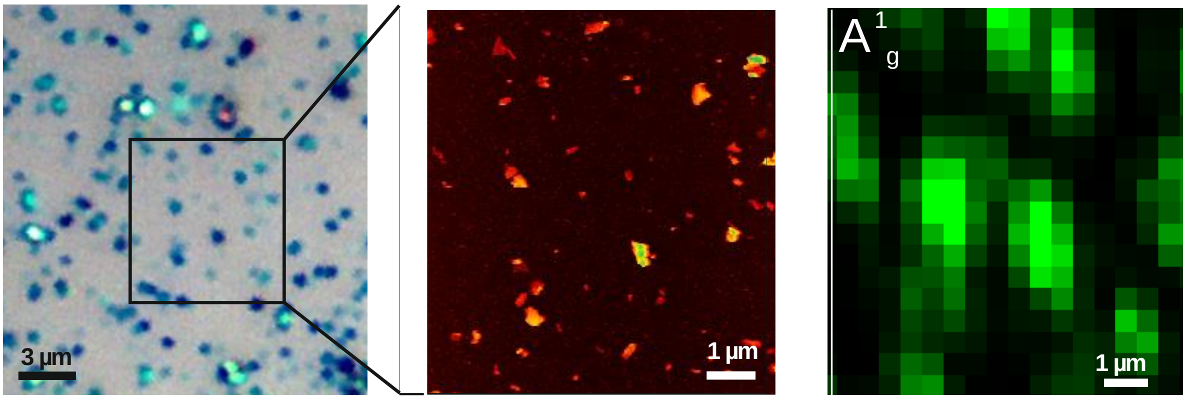

Supplementary Figure 7 | AFM-Raman relocalisation. The relocalisation makes use of the optical contract of nanomaterials deposited on opaque bilayered substrate. From left to right: optical overview micrograph of the region subjected to AFM and Raman. Middle: AFM image of the region marked by the rectangle in the optical micrograph (identified by optical zoom/camera of the AFM). Right: Raman map of the same region.

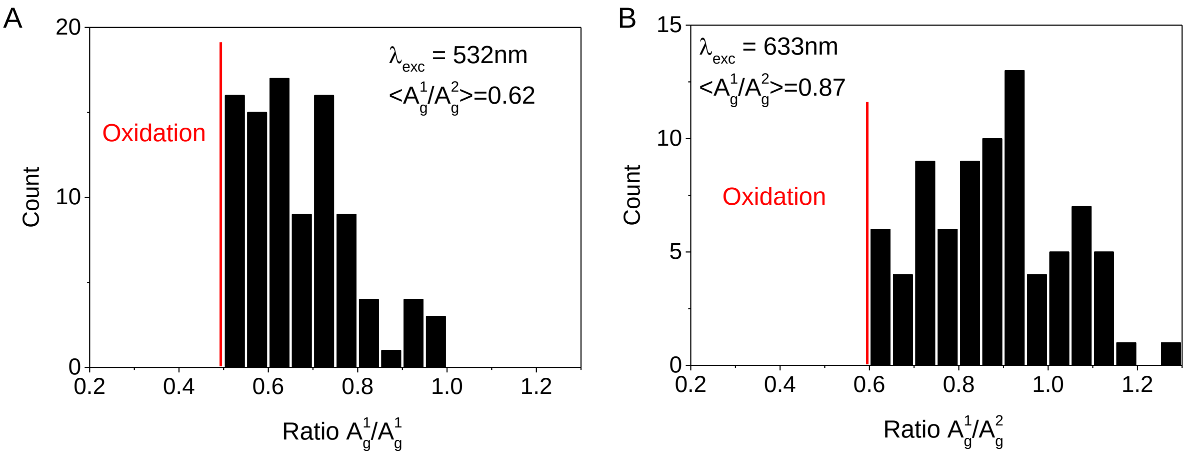

Supplementary Figure 8 | Dependence of A^1_g/ A^2_g ratio on the excitation wavelength. Histogram of the intensity ratio of the Raman A^1_g/A^2_g modes obtained from the analysis of 120 baseline corrected spectra acquired over an area of 25x25 μm^2 measured with different excitation energies. A) 532 nm, B) 633 nm. While the A^1_g/A^2_g intensity ratio is lower (both in mean and the lowest values obtained) when measured with an excitation wavelength of 532 nm, it is nonetheless consistent with negligible basal plane degradation.[7]

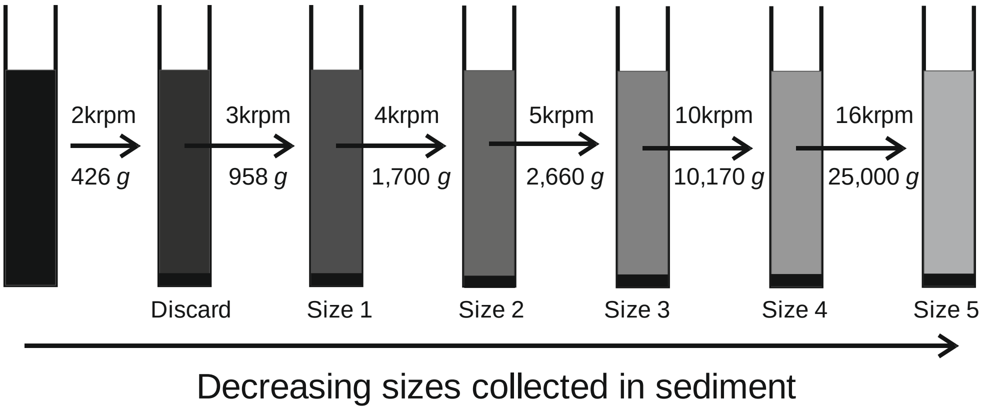

Supplementary Figure 9 | Size selection procedure 1. Schematic representation of the controlled centrifugation protocol applied to select a broad variation of sizes from a raw dispersion. Starting with an as-sonicated dispersion, we centrifuge at 2 krpm (424 g) for 2 h and decant the supernatant while the sediment is discarded. In this case the sediment is removed, as it is composed of primarily unexfoliated nanosheets. The supernatant is subjected to another centrifugation at 3 krpm (958 g) for 2 h. Again the supernatant is decanted and moved to the next centrifugation step at higher rpm, while the sediment is redispersed in fresh solvent (CHP) by mild agitation and collected. The process of decanting the supernatant and redispersing the sediment in fresh solvent is employed through multiple steps to achieve varying size distributions of nanosheets in the respective dispersions. Repeating this procedure yields multiple dispersions, where the lateral dimensions of the nanosheets decrease with increasing centrifugation speed. The centrifugation speeds are indicated in the figure. This protocol bears a number of advantages over analysing the supernatant after centrifugation at different rpms: i) basically no material is lost, as both sediment and supernatant is collected, ii) due to redispersing the sediment, the concentration can be adjusted and controlled by the volume of fresh solvent added and iii) it is universal and can be applied to any solvent, surfactant, or polymer system. Statistical TEM analysis was performed on all size selected samples showing that dispersions with mean lateral dimensions ranging from 150-620 nm (Fig 3A-C and Fig. S11).

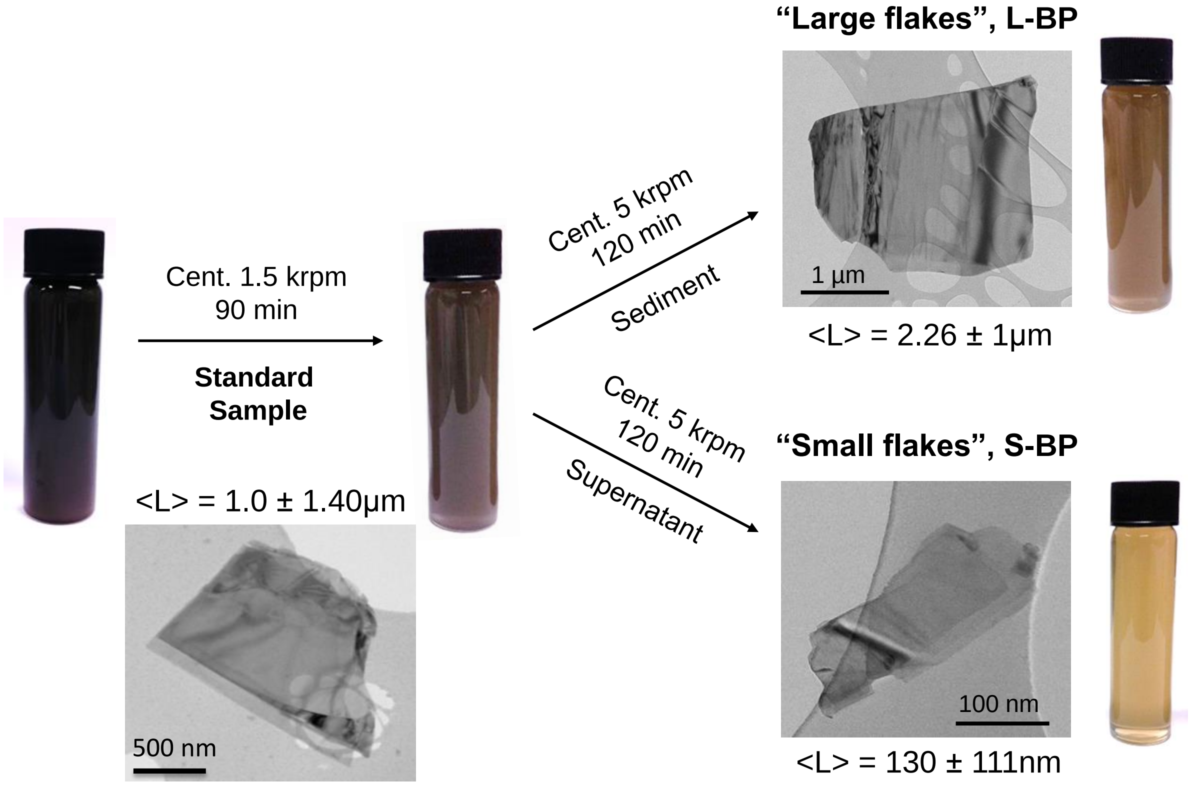

Supplementary Figure 10 | Size selection procedure 2. Schematic representation of the size selection to obtain "small" and "large" nanosheets used throughout main manuscript. The procedure described in supplementary figure 9 is highly beneficial when multiple dispersions with varying flake size distributions are targeted. However, for the study on degradation of smaller *versus* larger flakes described in the main manuscript, we have employed a simple procedure as outlined below. We have subjected the std-BP dispersion to one more centrifugation step at medium speed (5 krpm, 2,660 g, 2 h) and collected both supernatant containing "small nanosheets", S-BP and redispersed the sediment containing "larger nanosheets", L-BP. This bears the advantage that we start from the well characterised std-BP dispersion that already only contains nanosheets that are not sedimenting, as the centrifugation time for the first centrifugation step was optimised (Fig. S1). This procedure gives dispersions with mean nanosheet lengths of 130 nm in the case of S-BP and 2.26 µm in the case of L-BP.

3.2 TEM of size selected samples

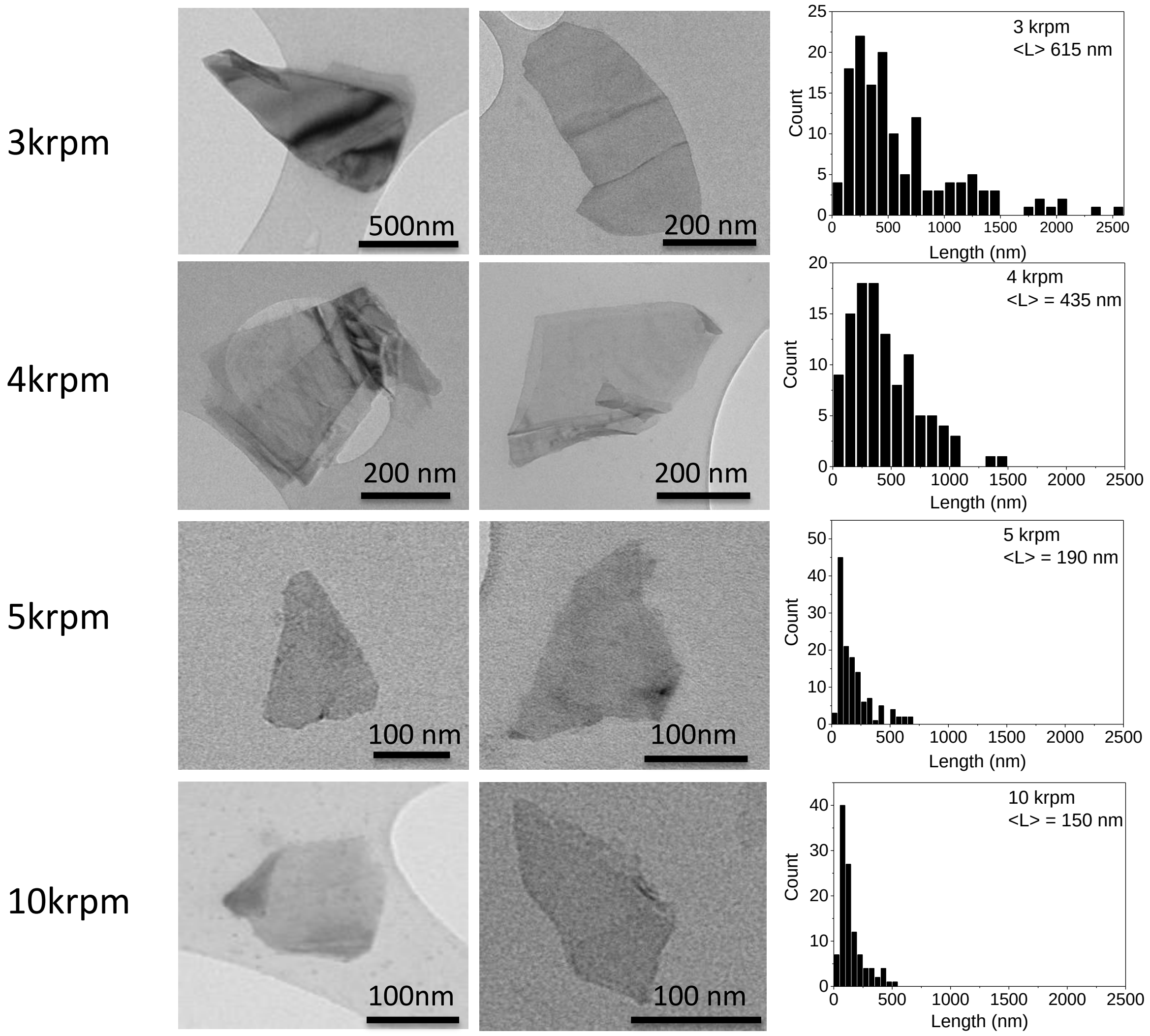

Supplementary Figure 11 | Statistical TEM analysis of size selected samples. Representative low-resolution TEM images and L histograms of size-selected FL-BP in CHP. Centrifugation speeds according to supplementary figure 9 are indicated in the left panels.

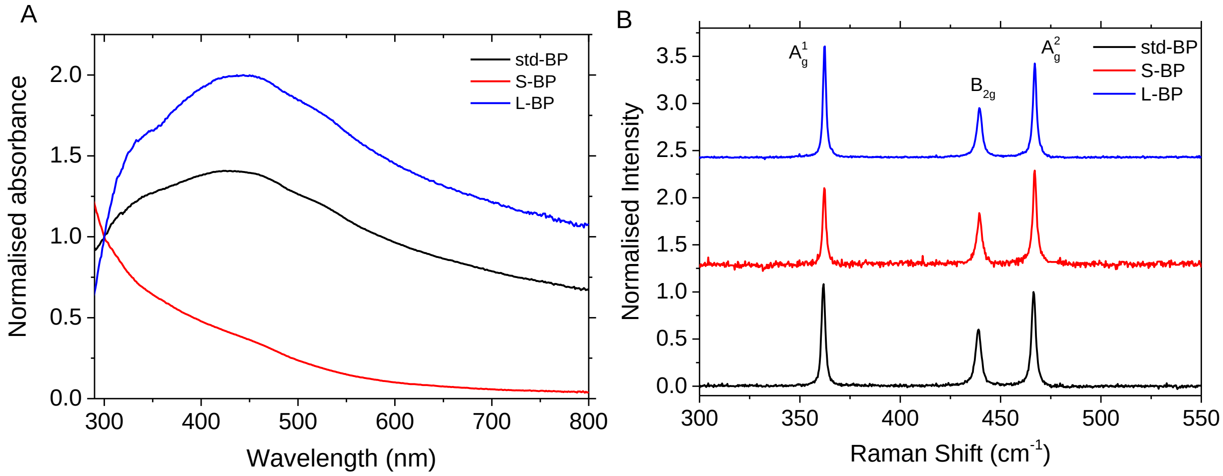

Supplementary Figure 12 | Optical characterisation of “small” and “large” FL-BP nanosheets. A) Absorbance spectra of S-BP and L-BP as obtained according to the size selection scenario 2 (supplementary figure 10) compared to the standard dispersion (all in CHP). Consistent with the data described in the main manuscript (figure 3D-F), larger flakes show a broader range of absorption while the smaller nanosheets absorb strongly at shorter wavelengths. B) Raman spectra of the dispersions dispersions (excitation wavelength 633 nm after filtration onto an alumina membrane and removal of the solvent through drying in a vacuum oven at elevated temperature. Analysis confirms that both small and large nanosheets retain the characteristic phonons associated with the bulk powder. We note that the laser intensity was kept below 0.2 mW due to pronounced sample heating on the alumina membranes (opposed to measurements after deposition onto wafers). The A^1_g/A^2_g intensity ratio of the S-BP sample is slightly lower in mean compared to std-BP and L-BP pointing towards a more pronounced degradation presumably due to edge effects (see below).

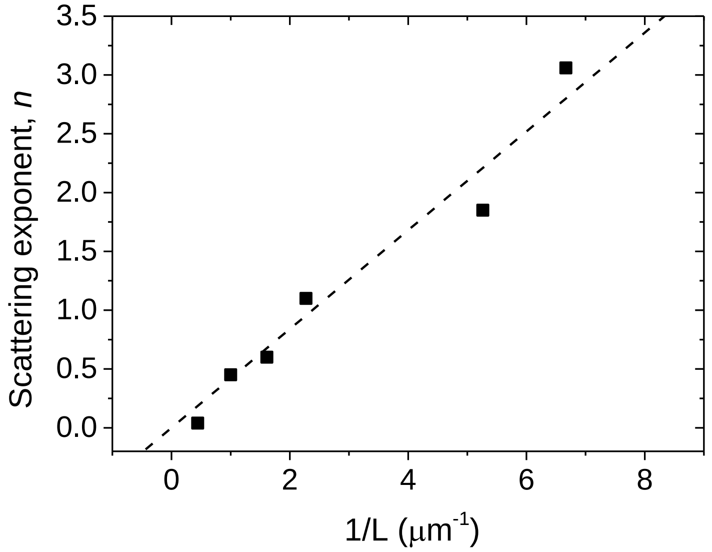

Supplementary Figure 13 | Scattering size metric. Long wavelength scattering exponent, *n*, plotted *versus* inverse nanosheet length. The scattering exponent was obtained by fitting the scattering spectra from 700-800 nm plotted on a log-log plot to a linear relation to obtain the scattering exponent –*n* as slope of the fit. The dashed line in the figure represents an empirical relation of *n* to the inverse nanosheet length. The nanosheet length can thus be determined from the scattering spectra by L(μm) = 0.42 / *n*.

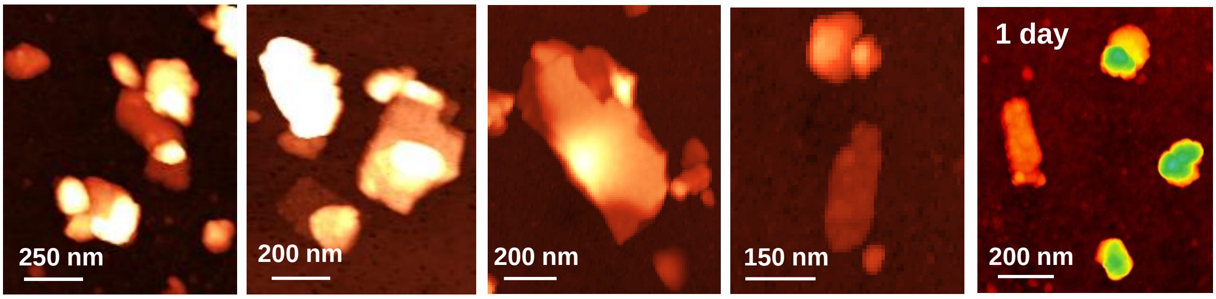

Supplementary Figure 14 | Basic characterisation of the BP exfoliated under inert conditions: AFM images. Representative AFM images of the std-BP samples produced from exfoliation in the glovebox. Nanosheets are similar in appearance to exfoliation under ambient conditions.

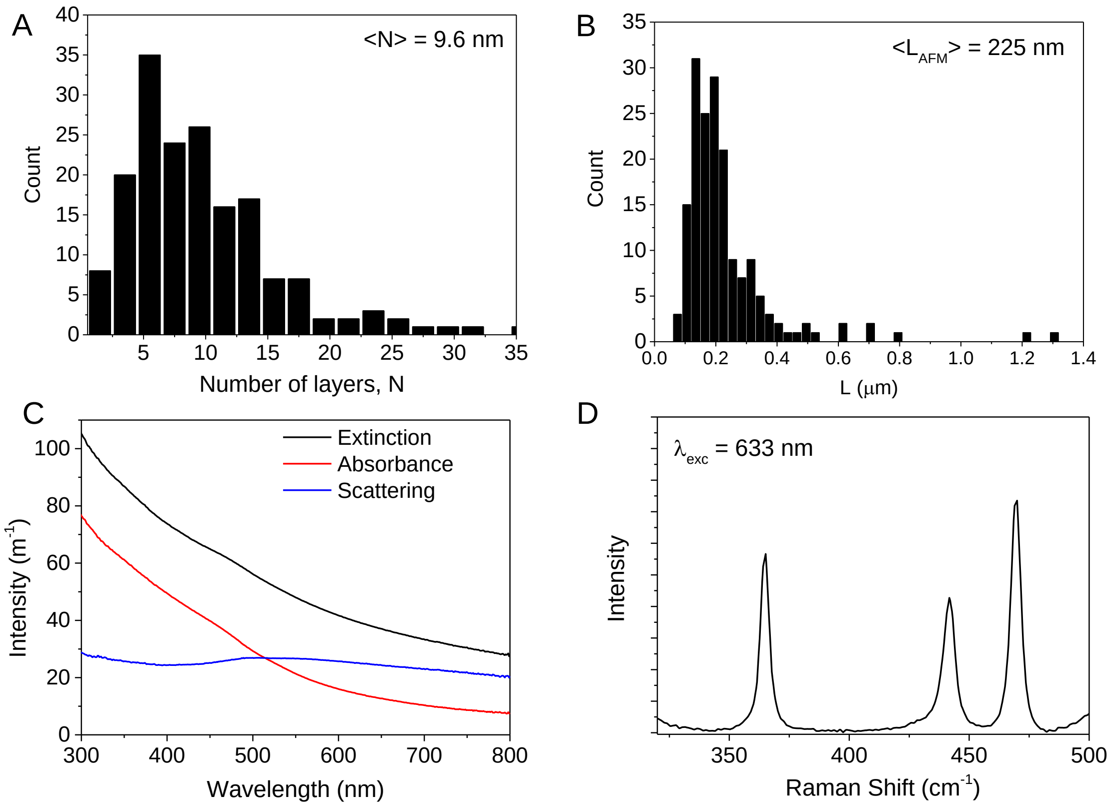

Supplementary Figure 15 | Basic characterisation of the BP exfoliated under inert conditions: Statistical AFM analysis and optical properties. A) Histogram of number of layers of the std-BP GB sample, B) Histogram of AFM length of the std-BP GB sample. Typically, length from AFM is not reliable in the case of polydisperse samples, as large nanosheets (> 500 nm) tend to be under represented thus introducing a bias towards the smaller nanosheets. However, the mean AFM length (225 nm) of the std-BP GB sample can still be compared to the std-BP exfoliated under ambient. In the latter, AFM L was determined as 365 nm. This suggests that the nanosheets exfoliated under ambient conditions are slightly smaller in lateral dimensions. We attribute this to the different sonicator that had to be used in the glovebox. C) Extinction, absorbance and scattering spectra of the std-BP GB sample in CHP. The smaller mean L is further confirmed by the extinction/absorbance/scattering spectra that show a lower contribution from scattering (compare to figure 1J in main manuscript) suggesting a smaller mean length. D) Mean Raman spectrum (over 120 individual spectra, excitation wavelength 633 nm). The A^1_g/A^2_g intensity ratio is slightly lower compared to the std-BP. This has also been observed for the S-BP sample exfoliated under ambient and is thus consistent with a slightly higher level of oxidation due to the smaller lateral dimensions (discussion of edge reactivity see below and main manuscript).

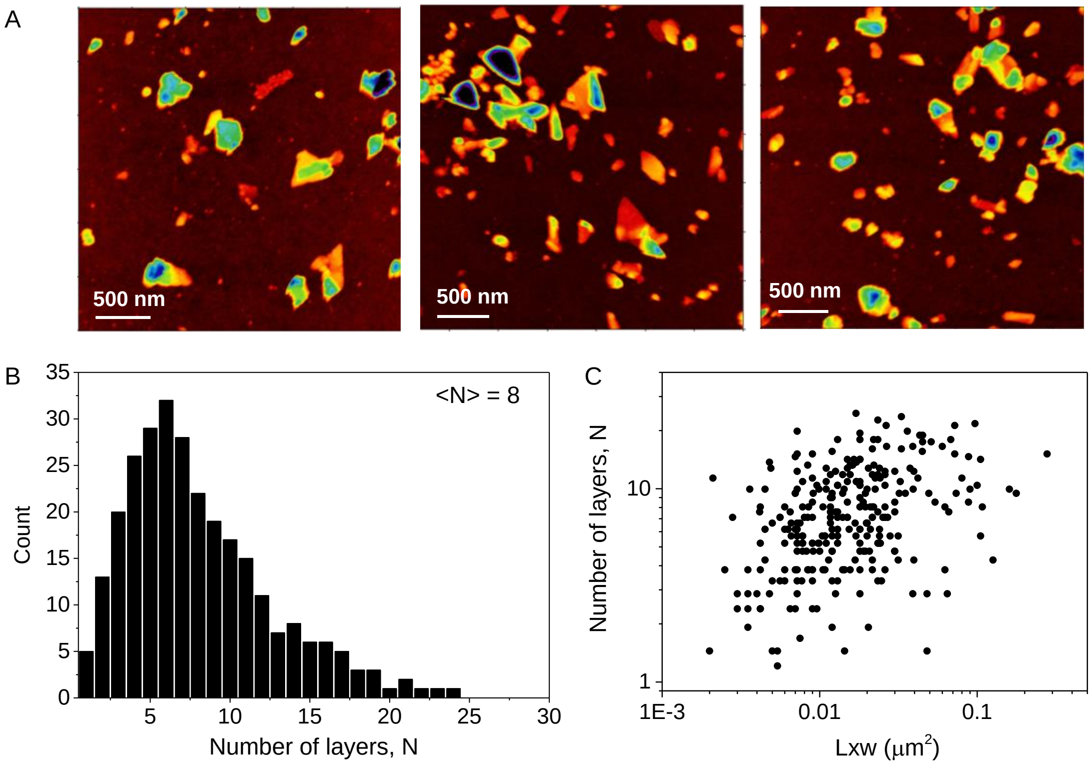

Supplementary Figure 16 | Statistical AFM analysis of the BP dispersion used for PL. A) Representative overview AFM images used for the statistical analysis of the small-BP-PL sample. Samples were transferred to IPA to facilitate deposition as described in the methods section. Multiple wafers were analysed that were exposed to ambient conditions for no longer than >2h to avoid a bias in the statistics due to adsorbed water. B) Histogram of number of layers from measurement of ~ 300 individual nanosheets. C) Plot of nanosheet thickness versus area (approximated as length x width) showing a much weaker correlation between thickness and area than observed when samples are prepared using standard centrifugation conditions. This implies that a potential effect of edges on PL properties are negligible, or at least similar across 1-5 layers.

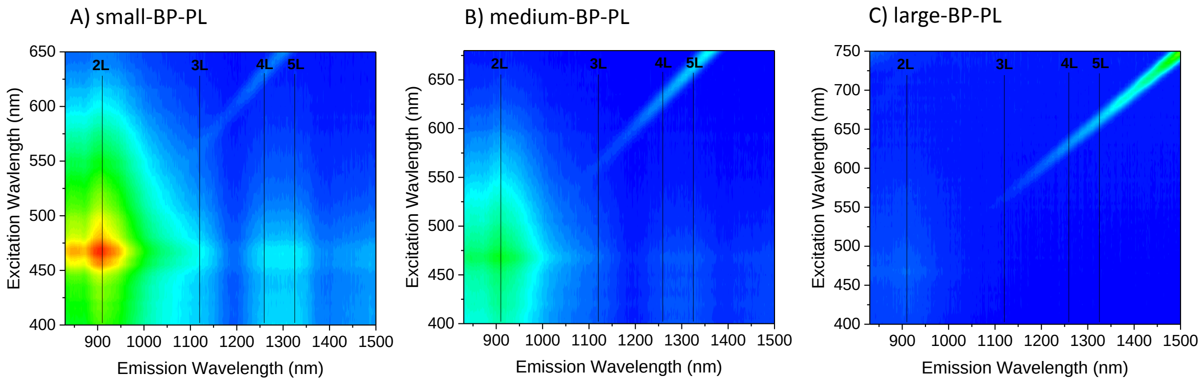

Supplementary Figure 17 | Photoluminescence spectroscopy: High wavelength excitation-emission contour plots (measured with 830 nm cut-off filter in emission) of FL-BP samples in CHP with three size distributions. The samples were diluted to the same optical density. A) small-BP-PL, B) medium BP-PL, C) large BP-PL. To study the fundamental properties of liquid exfoliated BP, the presence of water and oxygen was minimised during the sample preparation by performing the sonication and all solvent transfer in a glove box as described in the methods section. This is not a prerequisite for the study, but rather a precaution. Since we expect the recorded PL intensity (under equal acquisition parameters and similar optical densities) to be dependent on the volume fraction of the 1-5 layered nanosheets, we have also measured PL on three samples with different thickness distributions (SI methods). Consistent with the expectation, the photoluminescence intensity (per optical density) is lower as nanosheets are (larger and) thicker. It is important to note that PL peak positions, quantum efficiencies and widths are strongly influenced by the static dielectric constant of the environment. This gives rise to quantitative deviations when comparing liquid-exfoliated BP to micromechanically cleaved BP deposited on substrates. The dielectric constants of pyrrolidone-based solvents are very high resulting in a low exciton binding energy.[8] The measured photoluminescence energy is therefore presumably very close to the optical gap of the BP (and also slightly higher than for micromechanically cleaved BP).

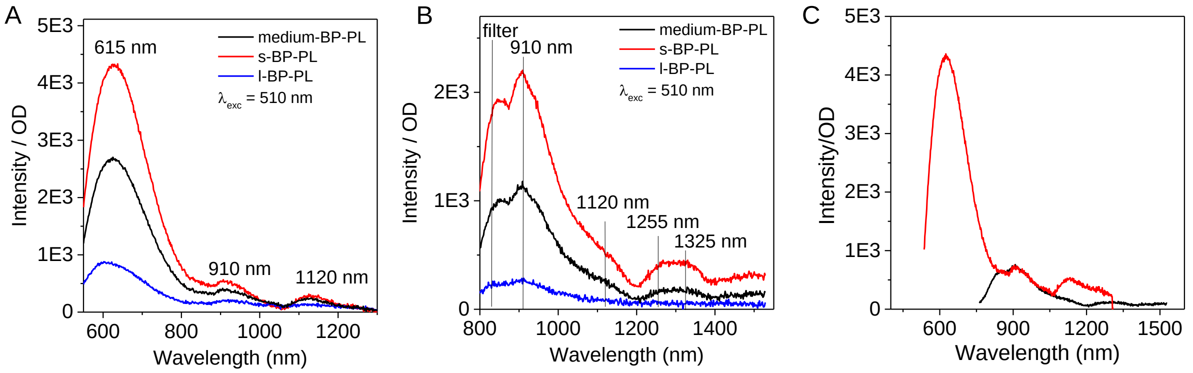

Supplementary Figure 18 | Photoluminescence spectroscopy: Single spectra. Emission single spectra at 510 nm excitation of the three different FL-BP samples. A) Low wavelength region measured using a 550 nm cut-off filter in emission, B) High wavelength emission measured using a 830 nm cut-off filter in emission. In this case, integration times were increased from 8 s to 15 s to resolve the relatively faint features. The spectra and contour maps were acquired using cut-off filters on the emission side to avoid the presence of the overtone of the excitation wavelength. Due to the broad spectral region of interest from ~550-1500 nm and strong variations in PL intensities, different cut-off filters and integration times were used (see SI methods). C) The spectra of the s-BP-PL were combined to yield the spectrum shown in the main manuscript after normalising the higher wavelength region spectrum to the lower wavelength spectrum at 910 nm. This was done by normalising the higher wavelength spectrum (measured with 830 nm cut-off filter) to the lower wavelength spectrum (measured with 550 nm cut-off filter) at 910 nm. As shown in supplementary figure 18C, this gives very good overlap in the spectral region of 850-1050 nm. In the case of the spectrum measured with the 550 nm cut-off filter, data points for > 1000 nm were discarded, while in the case of the spectrum measured with the 830 nm cut-off filter, data points at < 1000 nm were discarded to yield the combined spectrum.

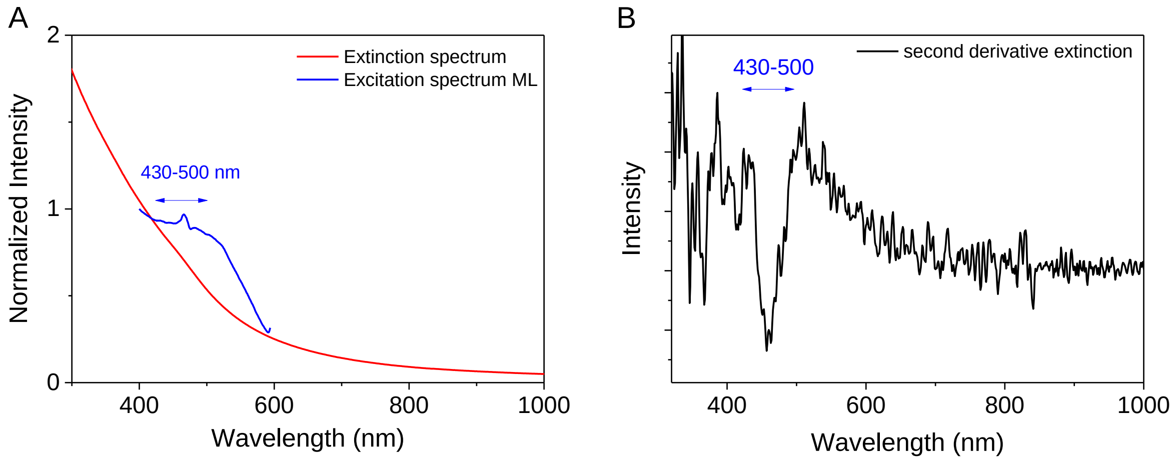

Supplementary Figure 19 | Photoluminescence spectroscopy: Extinction and excitation spectra. A) Extinction spectrum of small-BP-PL and excitation spectrum at the 615 nm emission showing a plateau region at 430-500 nm. We note that the spectral range for the acquisition of the excitation spectrum was limited to ~ 0.5 x 615 – 615 nm. The excitation spectrum shows a plateau region at 430-500 nm. The signal from the 2-5 layer species was very weak and no valuable information could be extracted from it. B) Second derivative of the extinction spectrum showing a peak in the same spectral range. Unfortunately, due to the polydispersity of the sample, no clear features are observed in the extinction spectrum. However, interestingly, when analysing the (smoothed) second derivative of the extinction spectrum, it is clear that a hidden component in the extinction spectrum is present at the same spectral region as the plateau of the excitation spectrum. This implies that the (randomly oriented) BP monolayer nanosheets strongly absorb at 430-500 nm which is reasonably consistent with reports on polarisation dependent measurements on micromechanically cleaved BP.[9]

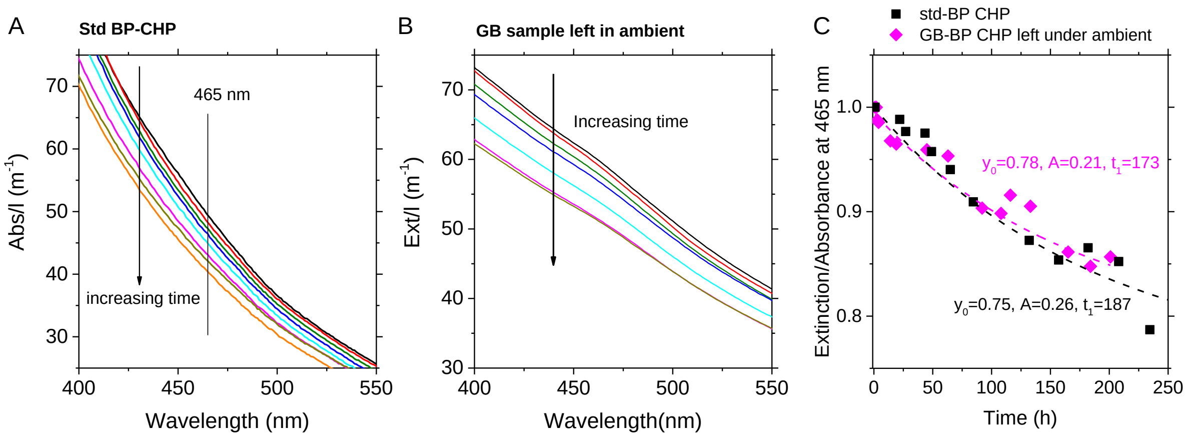

Supplementary Figure 20 | Tracking BP degradation by UV-Vis spectroscopy: Absorbance *versus* extinction. A) Absorbance spectra of a standard dispersion in CHP over time showing sample degradation. B) Extinction spectra of the std-BP exfoliated in the glovebox after exposure to ambient conditions showing similar spectral changes over time. C) Plot of normalised absorbance/extinction decrease as a function of time. The data from both samples (exfoliated under ambient and under inert gas conditions) measured in absorbance and extinction, respectively, follows the same exponential decay. For a detailed discussion on BP degradation see Supplementary Note 1.

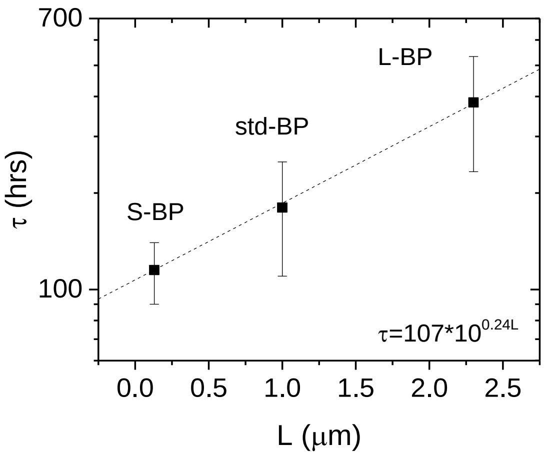

Supplementary Figure 21 | Tracking BP degradation by UV-Vis spectroscopy: Impact of nanosheet size. Degradation time constant plotted *versus* mean nanosheet size for std-BP, S-BP and L-BP in CHP. For a detailed discussion on BP degradation see Supplementary Note 1.

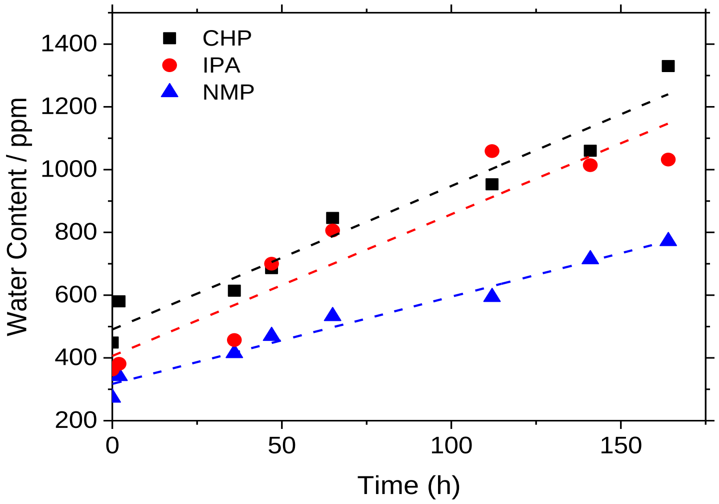

Supplementary Figure 22 | Tracking BP degradation by UV-Vis spectroscopy: Water content of the solvents (determined by Karl Fisher titration) used in this study as a function of time. The data point at 0 h is defined as the moment the sealed bottle was opened for the first time. For a detailed discussion on BP degradation see Supplementary Note 1.

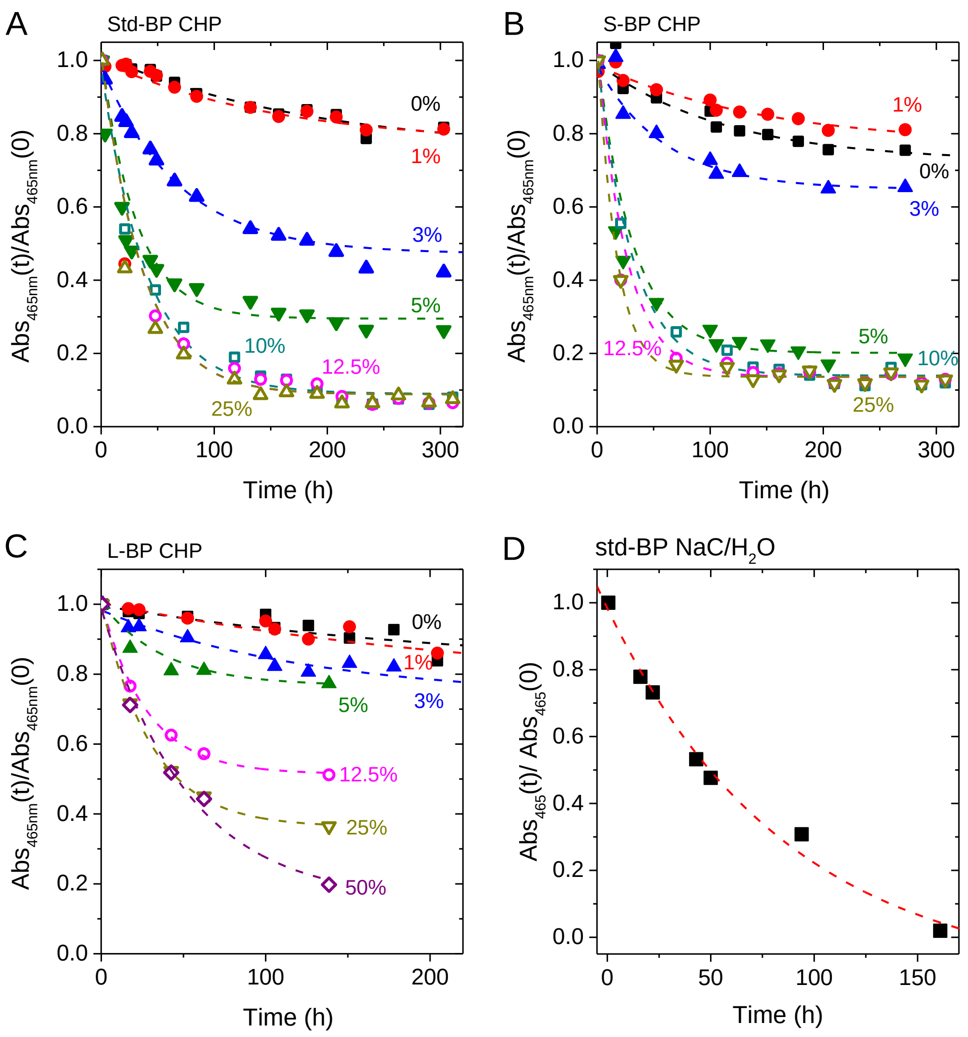

Supplementary Figure 23 | Tracking BP degradation by UV-Vis spectroscopy: The role of water. Absorbance monitoring as a function of time of FL-BP in CHP for different volume fractions of water added. A) std-BP, B) S-BP, C) L-BP. For a detailed discussion on BP degradation see Supplementary Note 1. D) Absorbance *versus* time for std-BP exfoliated in an aqueous solution of the surfactant sodium cholate, NaC. The NaC concentration was 6 g/L. All BP degrades after ~160 h due to the effectively infinite reservoir of water and oxygen.

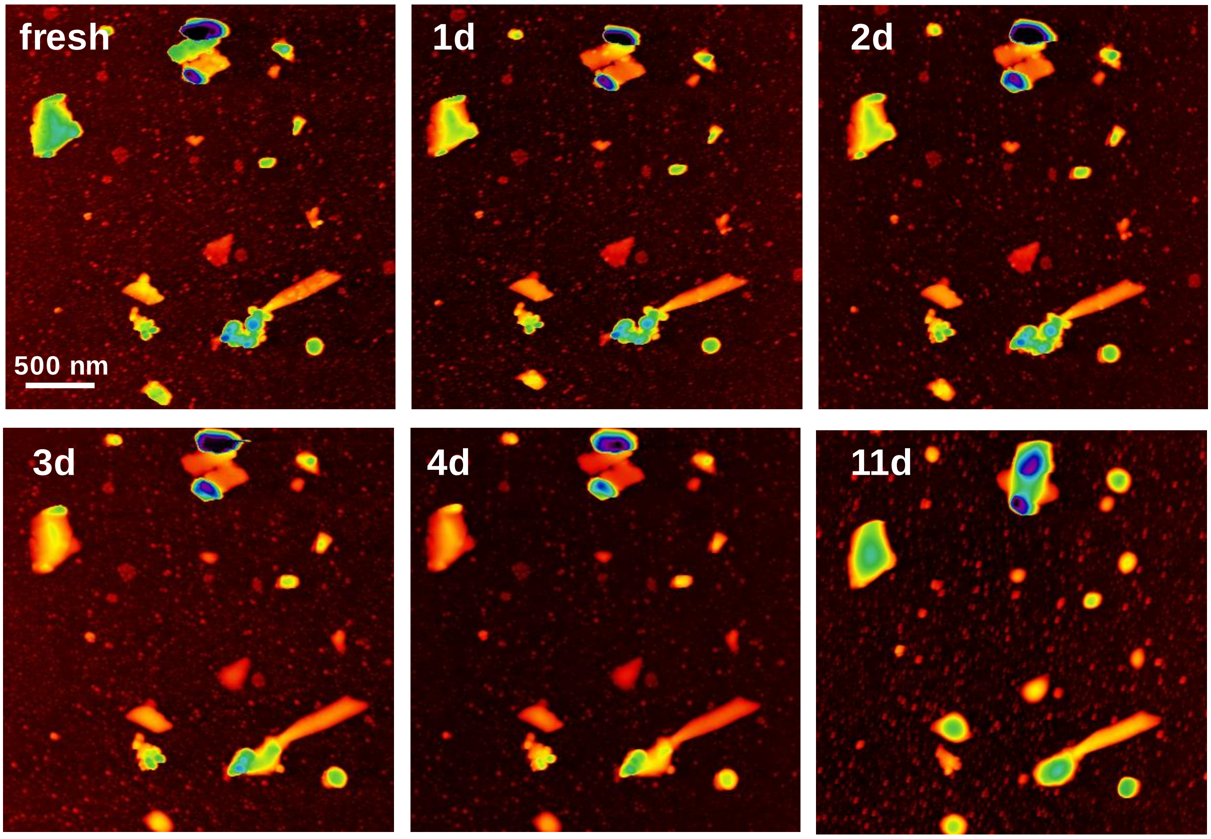

Supplementary Figure 24 | Stability of std-BP exfoliated in CHP: AFM Overview AFM height images of deposited std-BP (after transfer to IPA). The same sample region was relocated after 1, 2, 3, 4 and 11 days, respectively. Initially, the nanosheets have sharp well defined edges and steps – a prerequisite for the step height analysis described in the main text. Tracking this area over time, we observe a softening of these features where the edges are no longer sharp becoming blob-like in nature while after eleven days the nanosheets become thicker and rounder in shape.

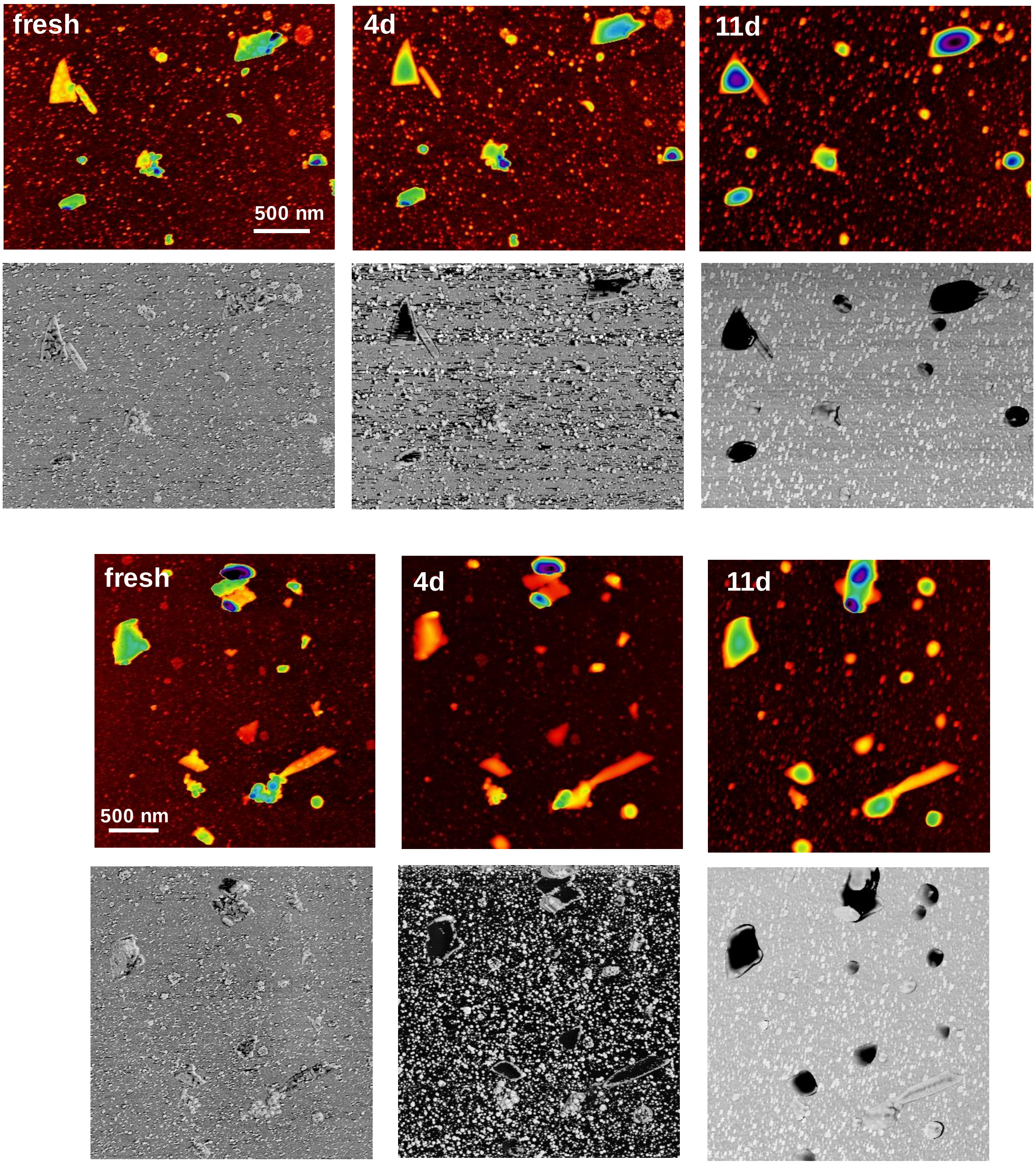

Supplementary Figure 25 | Stability of std-BP exfoliated in CHP: AFM. Zoomed-in AFM height (rainbow colour code) and phase (grey colour code) images of deposited std-BP (after transfer to IPA). The same sample region was relocated after 4 and 11 days, respectively. In regions of increased apparent height of the FL-BP nanosheets, a significantly increased phase contrast is observed. This can be attributed to adsorption of a material (presumably water) with a significantly different interaction with the cantilever. This is unfortunate, as it makes an analysis an accurate determination of layer number impossible in the case of samples exposed to ambient conditions already after a few hours.

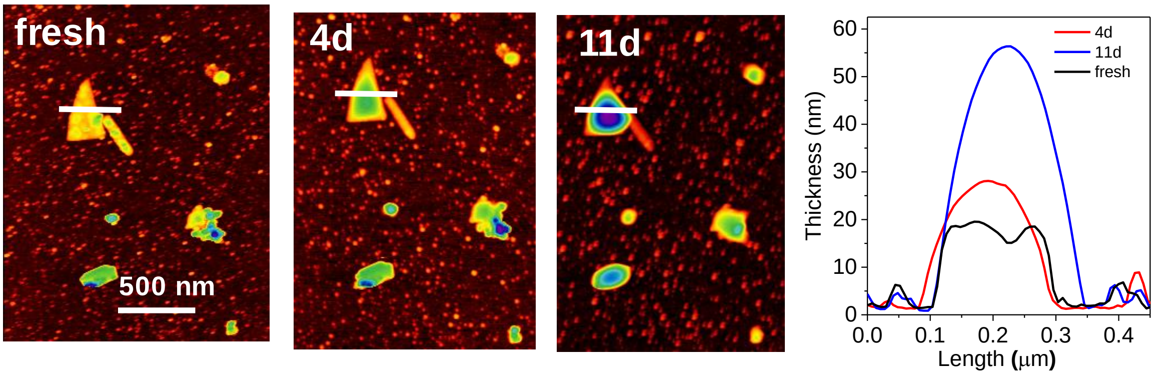

Supplementary Figure 26 | Stability of std-BP exfoliated in CHP: AFM. Sequence of AFM height images of deposited std-BP (after transfer to IPA) and height profile along the white line. The height profile becomes drop-like in shape suggesting adsorption of water

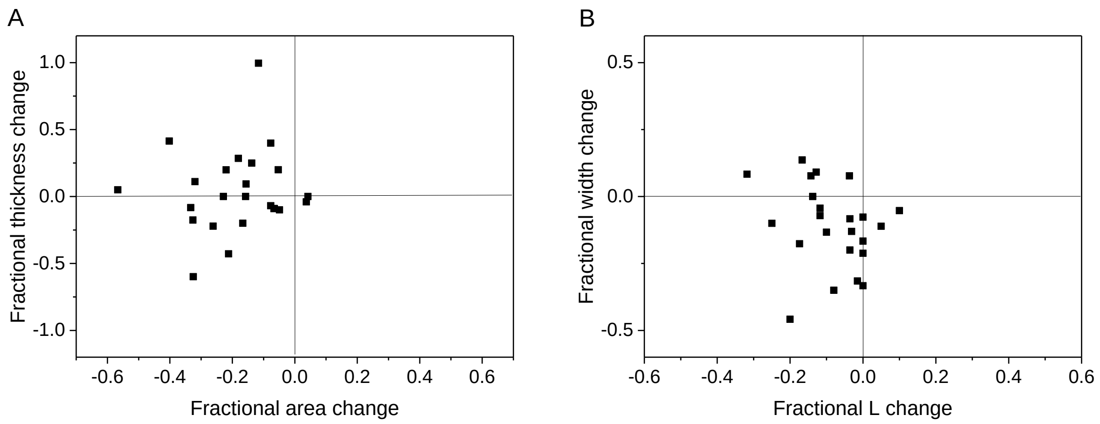

Supplementary Figure 27 | Stability of std-BP exfoliated in CHP: AFM shrinking. The data from degradation in liquid on S-BP compared to L-BP suggests that the degradation starts from nanosheet edges. We have therefore attempted to analyse this by AFM on the deposited nanosheets by measuring length, *L*, width, *w*, and thickness, *t*, on a number of nanosheets after ageing. Unfortunately, the water adsorption is too pronounced after three days or more also leading to an apparent increase in lateral dimensions as shown in the height profile in supplementary figure 26. The data after >4 days was therefore not analysable. A) To test whether we observe shrinking, we plot the fractional thickness change as a function of the fractional area ($l \times w$) change. This means that we divided the thickness and area, respectively, of a given nanosheet after two days ageing by the dimensions of the same nanosheet when it was imaged directly after production and deposition. From this data, it is clear that there is no trend in apparent height changes. However, despite the scatter in the data, it is evident that the nanosheets consistently shrink without correlation to their thickness. This again strongly suggests that degradation of FL-BP starts from the edges and not the basal plane. B) Fractional width change of deposited FL-BP nanosheets after two days plotted as a function of fractional length change. The data clearly shows that the majority of the nanosheets are shrinking in lateral dimensions.

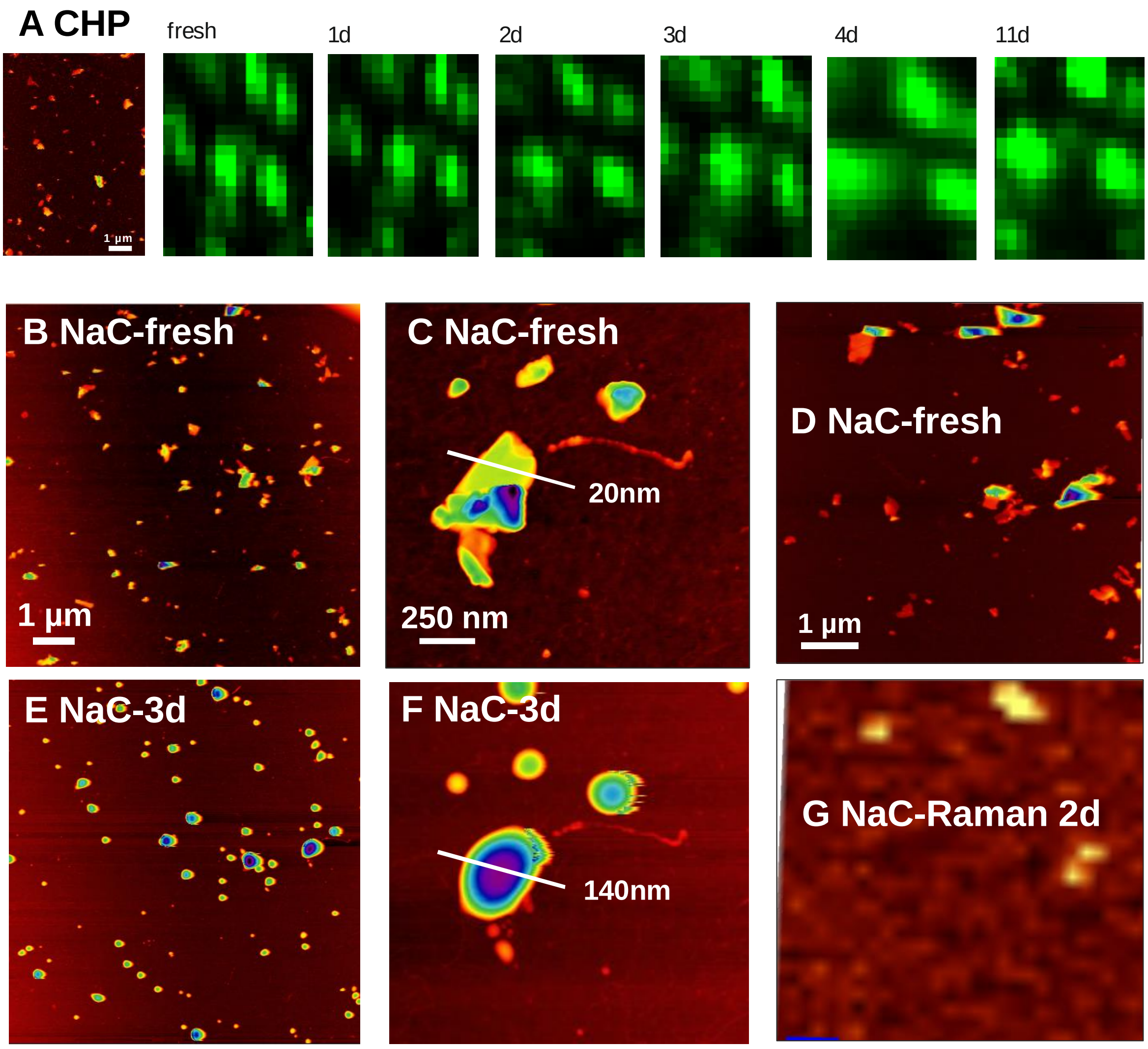

Supplementary Figure 28 | Stability of std-BP exfoliated in CHP and NaC: Raman. A: Tracking degradation of BP from CHP by Raman. The same sample area was relocated under the Raman microscope the same day the AFM was performed. The AFM image and the Raman A^1_g intensity maps of the same sample region after exposure to ambient conditions for 1-11 days is shown. While the Raman intensity maps of the region appear to blur out, this cannot be related to the ageing time, as it is very challenging to maintain the same focus of the laser beam on the sample. Importantly, we do not observe any spectral changes, shifts in phonon peak positions, additional Raman modes etc (see main manuscript figure 5I). We thus conclude that, even after 11 days, the CHP exfoliated FL-BP is still reasonably intact. B-G) Degradation after deposition from aqueous NaC. B-D) AFM images of freshly prepared BP. E-F) Same sample areas as in B and C respectively after 3 days of exposure to ambient. The nanosheets have completely disintegrated. G) Raman A^1_g intensity map of the sample region in D after 2 days of exposure to ambient. Only the formerly largest nanosheets show remaining Raman signal.

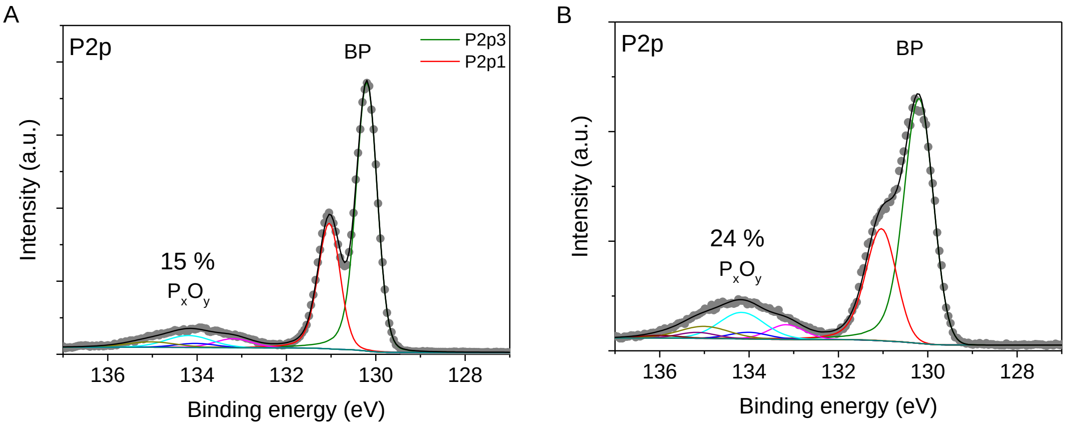

Supplementary Figure 29 | Stability of std-BP exfoliated in CHP: XPS. Fitted XPS P2p core level spectra of std-BP after filtration. A) fresh sample, B) after ageing for three days in dispersion. Analysis of the reaction products of the degradation using XPS showed that a "fresh" std-BP film (as deposited) showed a P_xO_y content of 15%. After 3 days ageing in dispersion, a second film was prepared and subjected to XPS. The P_xO_y species increased to 24% suggesting that the degradation of the FL-BP is a result of the reaction with water under ambient conditions also in the dispersion. The increased percentage of P_xO_y species is perfectly consistent with the 8 % loss of material in the std-BP after 3 days monitored by absorbance spectroscopy. The P_xO_y species had to be fit with four components suggesting the subsequent reactions occurred after an initial oxidation OR disproportionation. In addition, the broadening of the $P2p_{1/2}$ and $P2p_{3/2}$ contributions in the core level spectra suggest a structural rearrangement and/or disruption of the FL-BP. We note that the top layer of the films after filtration which was exposed to the environment during filtration and drying was removed by scotch tape prior to XPS due to the surface sensitivity of the measurement.

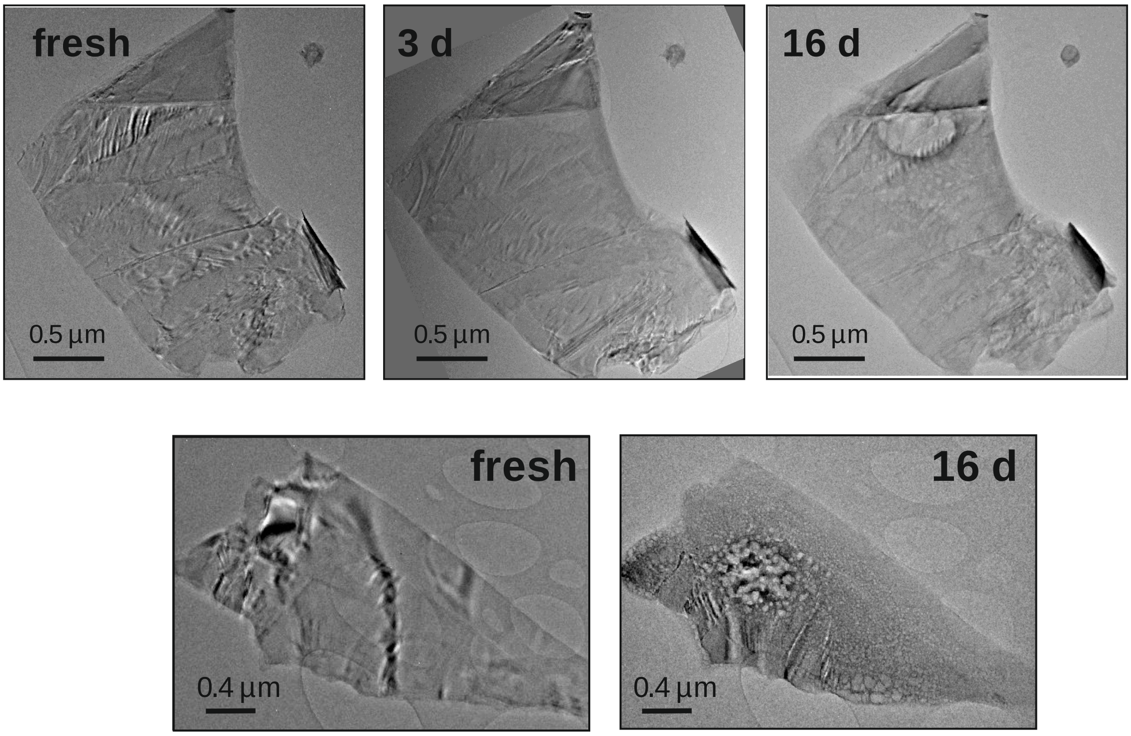

Supplementary Figure 30 | Stability of std-BP exfoliated in CHP: TEM. Sequences of TEM images of the same nanosheet (std-BP-CHP) directly after deposition, after 3 days and 16 days of ageing, respectively. Totally consistent with AFM, the edges blur out and soften. In addition, accumulation of droplets is frequently observed on the basal plane. This is accompanied with beam damage on the basal plane during imaging suggesting that the droplets are adsorbed water that reacts also with the basal plane under the electron beam.

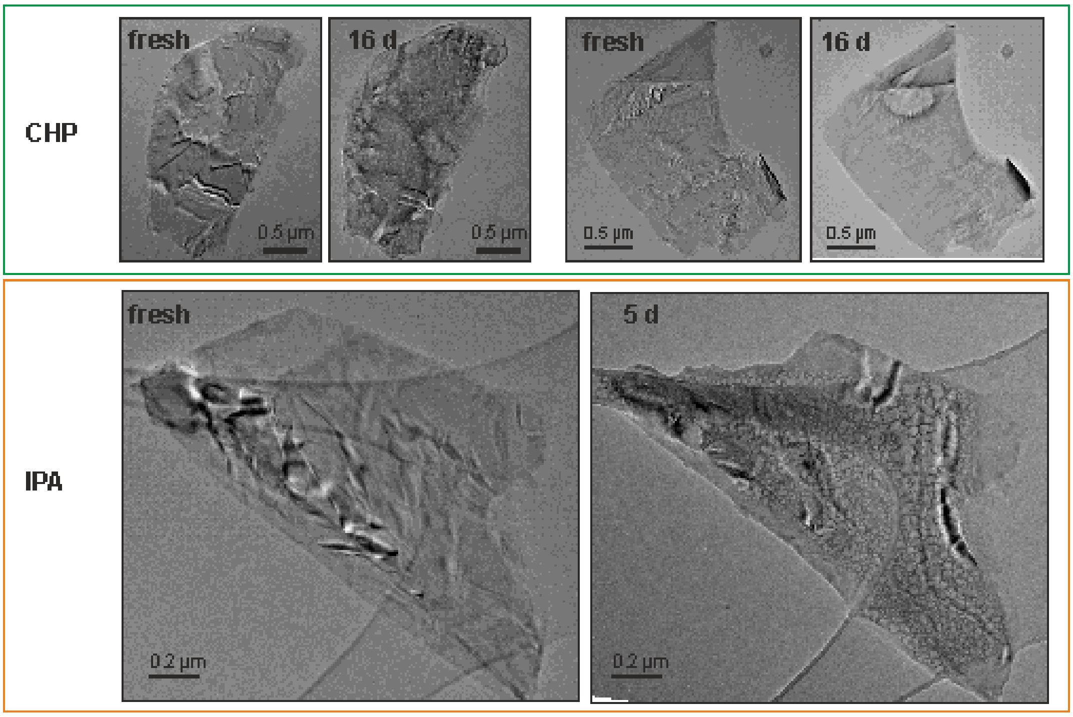

Supplementary Figure 31 | Stability of std-BP exfoliated in IPA: TEM Top: TEM images of FL-BP exfoliated in CHP. The nanosheets are reasonably intact after >16 d. Bottom: FL-BP exfoliated in IPA. Visual disintegration of the flake occurred already after 5 days. We thus conclude that, while beneficial for HRTEM, IPA is evaporated easily from the FL-BP after deposition leaving the flakes exposed to the ambient condition. The high boiling point of CHP is favourable in this case, as it leaves a protective solvent layer on the exfoliated sheets also slowing down the degradation after deposition.

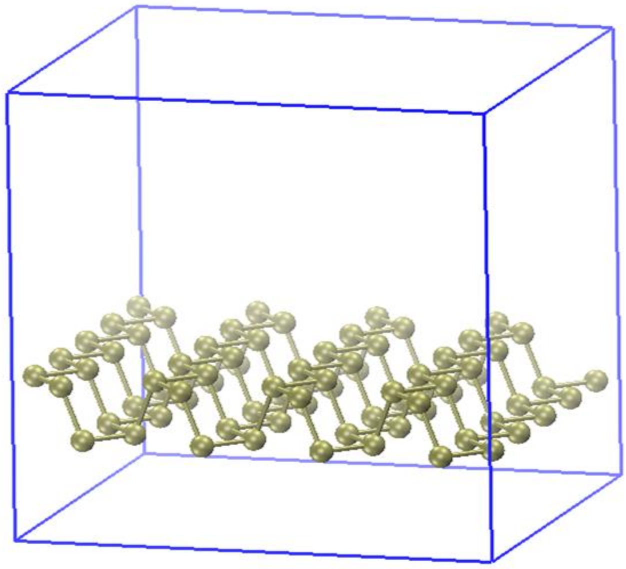

Supplementary Figure 32 | Supercell used to model a defect-free BP monolayer. The cell is constructed by replicating the BP unit cell 5 × 4 times along x and y, respectively. The supercell size is *a*=16.579 Å, *b*=18.465 Å, *c*=18.22 Å. Periodic replica of the monolayer along z are at a distance of about 16 Å, so that the layers should be considered as non-interacting. The peculiar BP structure is characterised by interconnected P atomic rows staggered along the z direction. The green spheres represent the P atoms.

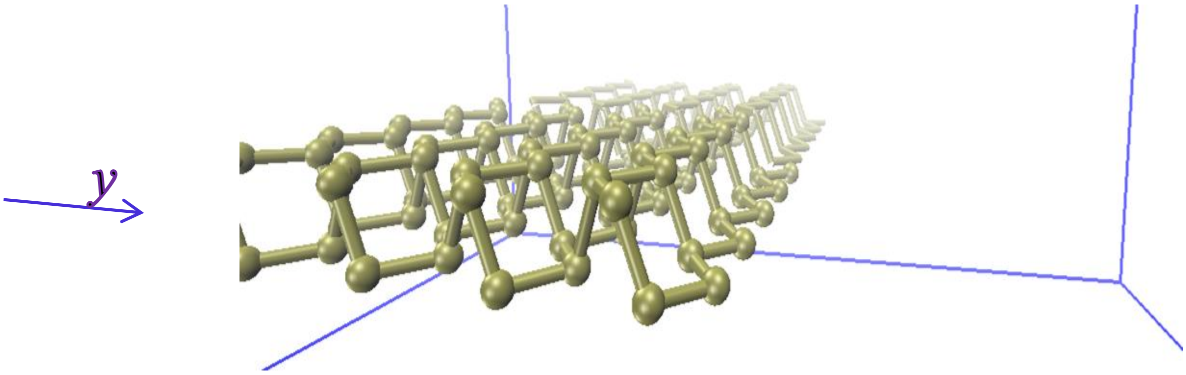

Supplementary Figure 33 | Model for the edge of a BP nanosheet. This is obtained by cleaving the bonds connecting the up and down atomic rows of an ideal BP monolayer along the x-direction. The system is periodic in the y-direction. For the simulations an orthorombic supercell is used, obtained by replicating the rectangular unit cell of the monolayer 5 x 4 times along the x and y directions, respectively. The supercell size is a=16.579 Å, b=35.033 Å, c=18.22 Å. Periodic replica of the nanoribbon along z and y are at a distance of approximately 16 Å and 17.5 Å, respectively (there is no interaction between the periodic replica). The relaxed structure of the edge is unreconstructed and terminates with an unsaturated zigzag row of P atoms. Green spheres represent P atoms.

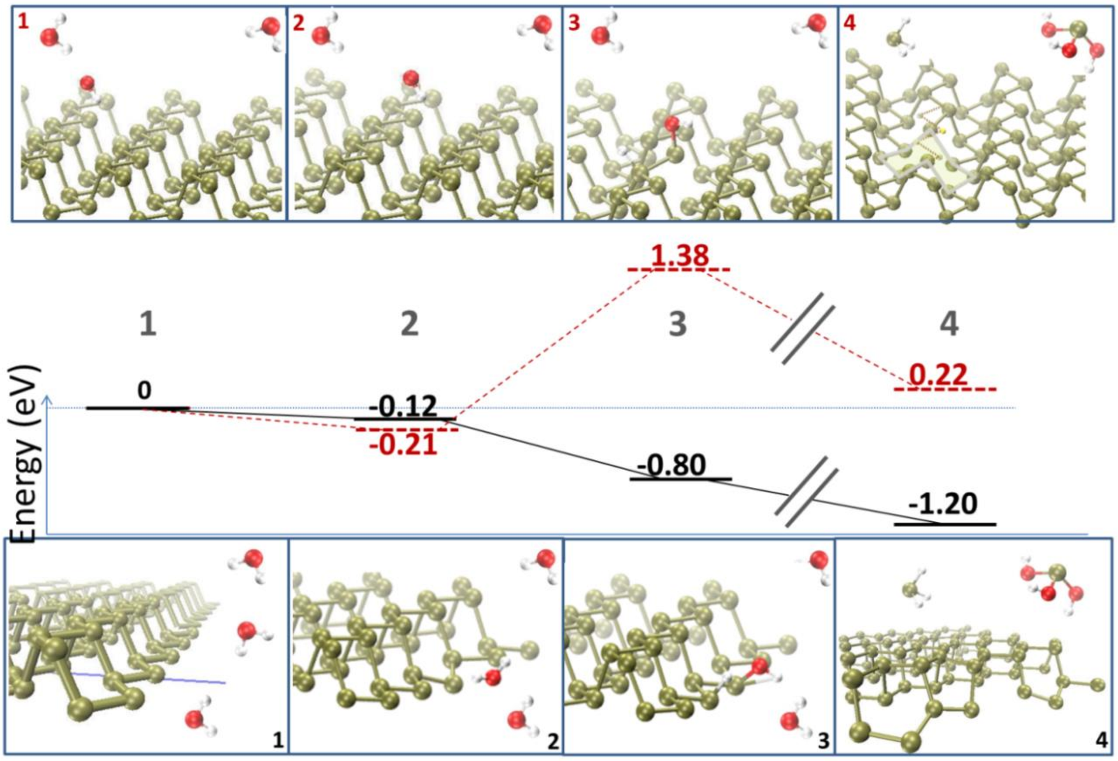

Supplementary Figure 34 | Illustration of the energy profile of the early stages of the reaction of water with BP and the final configuration. Reaction at the basal plane: images at the top and red profile. Reaction at the edge: images at the bottom and black profile. Discussion see Supplementary Note 2.

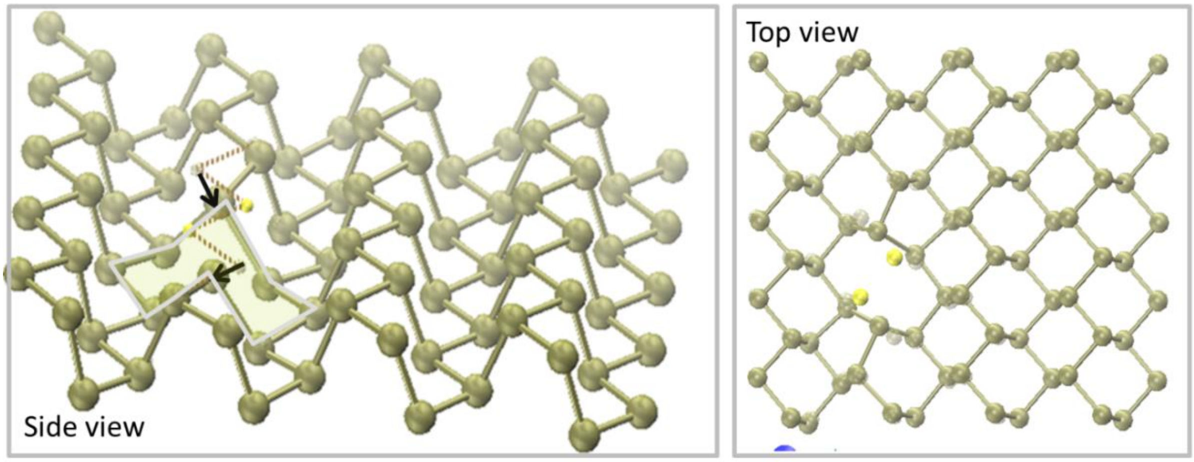

Supplementary Figure 35 | Side and top view of the defective nanosheet after PH_3 and H_3PO_3 release. This leads to the formation of a hole on the surface. Undercoordinates P next to vacancies (highligted in yellow) relax toward the lower BP plane to mantain 3-folded coordination. Transparent green spheres show the position of P atoms adjacent to the vacancies before geometry optimization. The black arrows show the relaxation pathway. Discussion see Supplementary Note 2.

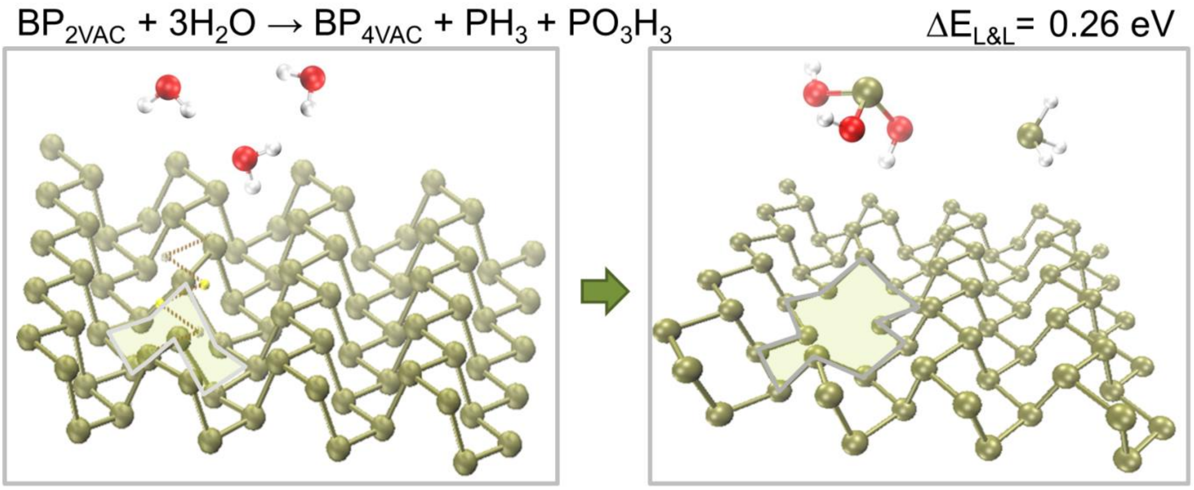

Supplementary Figure 36 | Degradation model for BP exposed to water attacking a hole in the middle of the nanosheet. Right and left panels represent the reagent (BP defective monolayer with the hole due to 2 P vacancies and three water molecules) and reaction products (BP defective monolayer with the big hole due to 4 P vacancies and both phosphine and phosporous acid), respectively. In the upper left part of the figure we report the reaction energy calculated at the level Langreth and Lundqwist DFT functional. Green, red and white spheres represent P, O and H atoms, respectively. The P vacancies are denoted as transparent green spheres. Discussion see Supplementary Note 2.

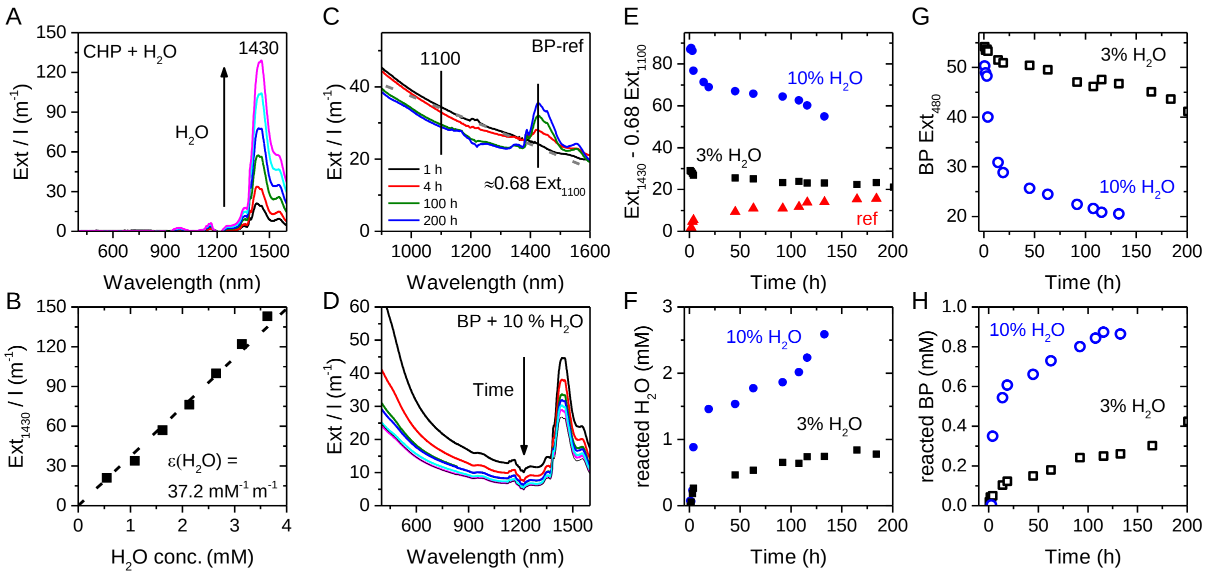

Supplementary Figure 37 | Experimental evaluation of the stoichiometry of the reagents in the BP degradation. A) Extinction spectra of different amounts of water added to CHP. B) Extinction coefficient of water in CHP at 1430 nm. C) Extinction spectra of BP exfoliated under inert gas conditions in dry CHP. The BP background at 1430 nm can be estimated to be 0.68 $Ext_{1100\ nm}$. In addition, the spectra show the uptake of water with time. D) Extinction spectra over time of BP in CHP after an initial addition of 10 vol% of water to accelerate the reaction rate. Both water and BP extinction decreases due to the degradation reaction. E) Plot of water extinction expressed as Ext_{1430nm} - 0.68 Ext_{1100nm} (to account for BP background) as a function of time for the reference sample and the samples after addition of 3 and 10 vol% of water. F) Plots of the reacted water as a function of time. G) Plot of extinction at 480 nm (attributed to only black phosphorus) as a function of time showing the characteristic exponential decay. H) Plots of the reacted BP as a function of time. Details see Supplementary Note 3.

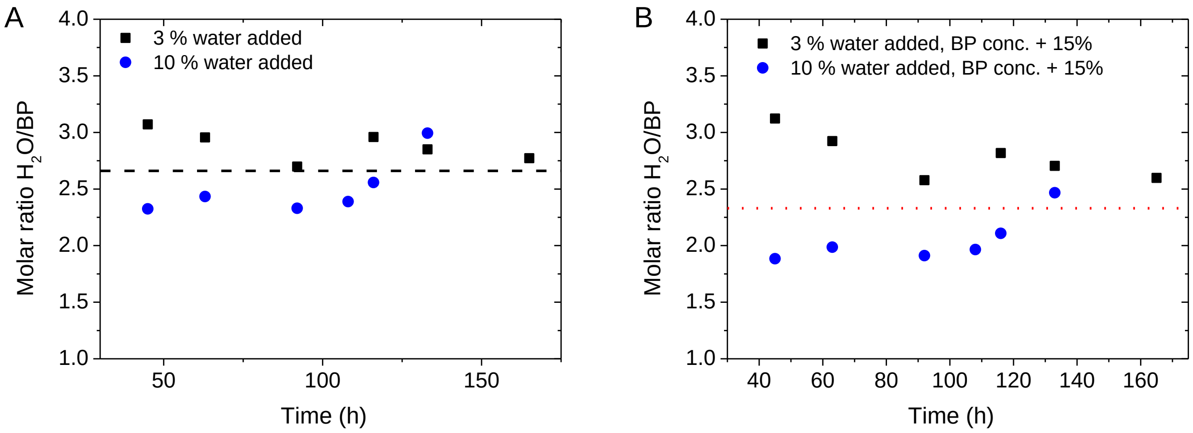

Supplementary Figure 38 | Impact of error in determining the extinction coefficient on stoichiometry ratio. A) plot of water/P stoichiometry ratio as a function of time as shown in the main manuscript compared to B) the same plot if assuming the extinction coefficient of BP to be overestimated by 15 %. Details see Supplementary Note 3.

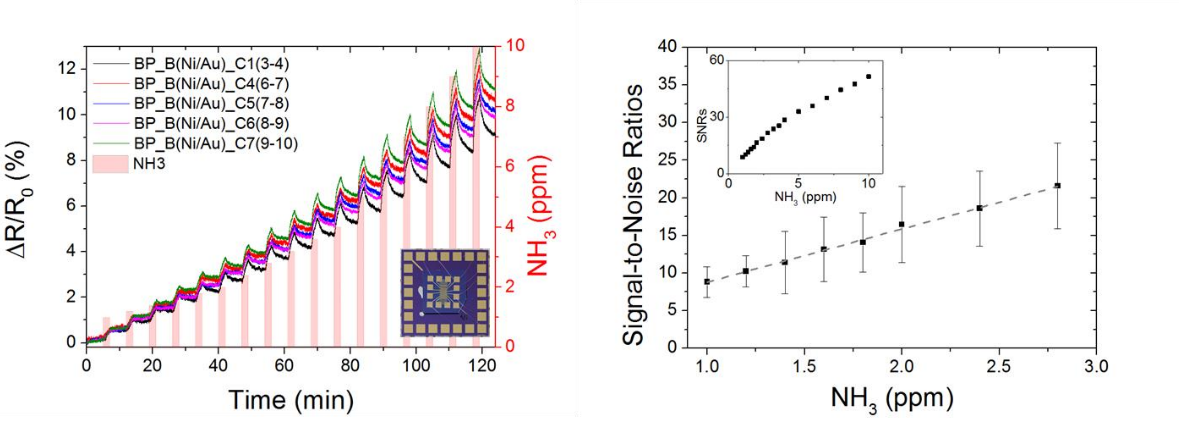

Supplementary Figure 39 | Gas sensing. A) Sensor response as a function of time overlayed increasing NH_3 concentration, B) Signal to noise ration with increasing NH_3 concentration. As NH_3 is an electron-donor, adsorbed NH_3 molecules on the surface of BP films shift the Fermi level to conduction band, resulting in a resistance increase consistent with p-type characteristic of BP films. Our empirical limit of detection (LOD) has been measured to be 1 part-per-million (ppm) with a signal-to-noise ratio (SNR) of 11.6. A theoretical LOD can be now extrapolated from the linearity of the SNR vs. NH_3 concentration as indicated in S41B. According to the IUPAC[10] the SNR must be at least a factor of three higher than the root-mean-square (RMS) of the noise. This suggested that with the BP sensor a detection threshold of 80 ppb is achievable. This extreme sensitivity maybe due to the fact that BP has larger adsorption energies of nitrogen-based gases than other 2D materials.[11-13] Thus we can expect to achieve better sensitivities than literature reports for non-functionalised nano-sensors.[14-16] It is also visible that recovery in pure N_2 flow is not complete at room temperature as its baseline gradually shifts during consecutive NH_3 injections. Tight-bonding between BP and NH_3 molecules may be responsible for this slow recovery, as it has been commonly observed in nanomaterial-based sensors at low working temperature. Annealing and ultra-violet light illumination have been suggested for acceleration of the recovery speed.[16, 17]

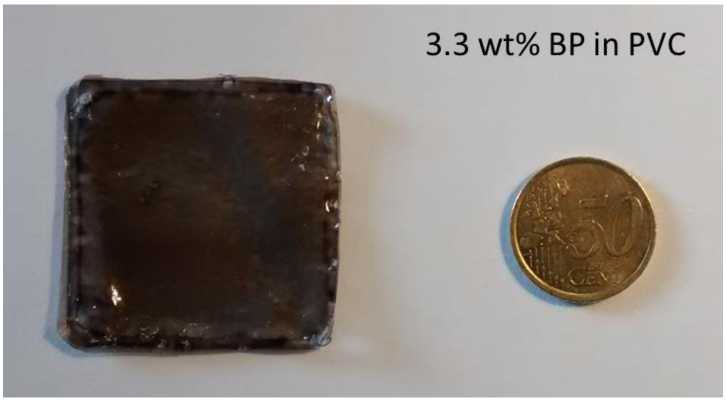

Supplementary Figure 40 | Photograph of BP:PVC composite film with high BP volume fraction. Composites of polyvinylchloride filled with various BP contents were produced as described above and in the main text (methods). While the low volume fraction composites were very uniform, the higher volume fractions displayed a degree of clumping, consistent with nanosheet aggregation.

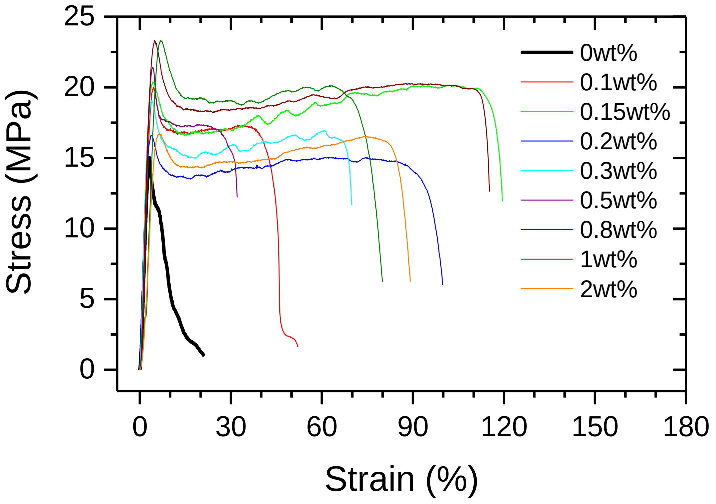

Supplementary Figure 41 | Full set of representative stress-strain curves for BP:PVC films with different BP loading levels from 0-2wt%.

	VOLUME FRACTION
1L	0.5 %
2L	2.2 %
3L	5.1 %
4L	6.1 %
5L	6.4 %

Supplementary Table 1 | Volume fraction of 1-5-layered BP. To compare relative internal quantum yields for 1-5 layered BP, we estimated the volume fractions of the respective nanosheet thicknesses by measuring length, width and thickness of ~300 nanosheets in AFM (supplementary figure 17). The as-determined volume fractions are summarised in table S1.

SAMPLE	L (nm)	$A_{Re} / (A_{UnRe} + A_{Re})$	τ (h)
L-BP	2300	0.19	233
Std-BP	1000	0.26	187
S-BP	130	0.27	114

Supplementary Table 2 | Kinetic degradation data for std-BP, S-BP and L-BP in CHP.

SOLVENT	$A_{Re} / (A_{UnRe} + A_{Re})$	τ (h)
CHP glovebox	0.1	300
CHP	0.26	190
NMP	0.28	275
IPA	0.5	115
NaC-H_2O	1	88

Supplementary Table 3 | Kinetic degradation data for std-BP in different solvents.

SAMPLE	$A_{Re} / (A_{UnRe} + A_{Re})$	τ (h)
Std-BP 1%	0.23	155
Std-BP 3%	0.5	67
Std-BP 5%	0.89	40
Std-BP 10%	0.7	30
Std-BP 12.5%	0.93	36
Std-BP 25%	0.93	25
Std-BP 50%	0.92	30
S-BP 1%	0.2	150
S-BP 3%	0.38	62
S-BP 5%	0.87	30
S-BP 10%	0.82	30
S-BP 12.5%	0.91	17
S-BP 25%	0.91	17
S-BP 50%	0.91	18
L-BP 1%	0.75	258
L-BP 3%	0.73	128
L-BP 5%	0.75	27
L-BP 12.5%	0.51	28
L-BP 25%	0.36	39
L-BP 50%	0.15	53

Supplementary Table 4 | Kinetic degradation data for BP in CHP after addition of water.

	a (Å)	b (Å)	c (Å)	V (Å^3)
**Present work**	**3.33**	**4.40**	**10.47**	**153.41**
**Appalakondaiah et al.[18]**	**3.30**	**4.40**	**10.43**	**151.3**
**Kozuki *et al.*[19]**	**3.31**	**4.37**	**10.47**	**151.77**

Supplementary Table 5: Computed lattice parameters, and unit-cell volume V, of bulk black phosphorus.

	c_{11}	c_{22}	c_{33}	c_{44}	c_{55}	c_{66}	c_{23}	c_{13}	c_{12}
**Present work**	**53.2**	**190.8**	**69.6**	**21.8**	**4.7**	**65.5**	**46.1**	**1.4**	**12.1**
**Appalakondaiah *et al.*[18]**	**52.3**	**191.9**	**73.0**	**25.5**	**8.8**	**63.6**	**40.8**	**0.6**	**8.3**

Supplementary Table 6: Computed stiffness tensor elements of bulk black phosphorus (GPa).

	G_{VRH}	B_{VRH}	Y_{VRH}	ν_{VRH}
**Present work**	**29.4**	**38.5**	**70.3**	**0.30**
**Appalakondaiah *et al.*[18]**	**25.0**	**40.0**	**62.0**	**0.24**

Supplementary Table7: Voigt-Reuss-Hill estimates of isotropic mechanical properties (the shear modulus G_{VRH}, bulk modulus B_{VRH}, Young's modulus Y_{VRH}, and Poisson ratio ν_{VRH}) of bulk black phosphorus (GPa).

Supplementary Note 1: Degradation of BP

Previous studies of mechanically cleaved black phosphorus have identified stability issues with nanosheets exposed to ambient conditions.[20-22] Here we have studied time dependent optical absorption and extinction over a period of 300 h to probe any change in our dispersed nanosheets (Supplementary figure 20A). Over time, a decrease in the absorbance

spectra is observed. We argue that any loss of FL-BP material by actual degradation, should be accompanied with loss of optical absorbance from FL-BP. Therefore plotting the absorbance at a fixed wavelength (such as 465 nm) can be used to track degradation (Supplementary figure 5A-B in the main manuscript).

To test whether an analysis of extinction spectra would also reflect the degradation and whether such an analysis is robust, we have compared the evolution of the absorbance of the std-BP over time to the extinction of the std-BP GB sample exposed to ambient conditions after the exfoliation (Supplementary figure 20). It is clear that both absorbance and extinction is reduced as BP is consumed as a result of degradation Supplementary figure 20C shows that both samples follow a similar exponential decay irrespective of whether absorbance or extinction is tracked. All degradation plots in the main manuscript except the std-BP GB stored under inert gas were generated using absorbance. The analysis of extinction spectra was mainly used to simultaneously track the consumption of water (Supplementary figure and Supplementary Note 3).

It is important to note that we do not observe any changes in peak intensity ratios or spectral shape in the absorbance/extinction spectra. This again suggests that degradation occurs from the edge, as any basal plane reaction would induce defects and potentially create new “edge-like” domains in the basal plane which would be manifested in spectral changes. Since this is not the case, any loss of FL-BP material by FL-BP degradation is only accompanied in a loss in absorbance intensity.

We fit the as-obtained absorbance versus time curves to an empirical exponentially decaying function of the form: $A = A_{UnRe} + A_{Re}e^{-t/\tau}$, where A_{Re} represents the total amount of FL-BP which reacts over time and A_{UnRe} represents the unreacted component. The fit constants are shown in supplementary table 2. Interestingly, $A_{Re} / (A_{UnRe} + A_{Re})$ ~ 0.25, widely independent on nanosheet size (except for the large nanosheets), implying that 25% of the BP to degrades in all cases (after an infinite time period). This suggests that the degradation to be limited by the amount of reagent (water/oxygen) present. Importantly, after 3 days, only ~8 % of the std-BP had dissolved (as confirmed by XPS, Supplementary figure 29), showing these systems to be stable enough to allow processing for applications.

As shown in Table S2 the experimental time constant associated with the exponential decay varies strongly with sample and so nanosheet size. This data is plotted in supplementary figure 21. Empirically, the time constant varies with nanosheet length as $\tau(\mathrm{hrs}) = 107 \times 10^{0.24L(\mu\mathrm{m})}$. Incidentally, this behaviour, although it has its basis in a complex,

multistep reaction, is approximately that which would be found for a simple reaction following first order kinetics with a rate constant given by $k(\mathrm{s}^{-1}) = 2.6\times10^{-6}\times10^{-0.24L(\mu\mathrm{m})}$.

During the course of our experiments, we also attempted to analyse the BP degradation in a number of different solvents. However, in most cases, we did not obtain stable dispersions with a significant enough concentration of exfoliated BP. This is important because we wanted to keep the initial optical density in the degradation studies constant to ensure comparability. In some cases, such as cyclohexanone, the concentration of exfoliated BP was high enough to conduct the optical measurements. The degradation data was nonetheless not reliable, as precipitation and reaggregation of the LPE-BP occurred after a few days which will likely impact the recorded optical densities and hence the time dependent degradation. The data is therefore not included in the discussion

Supplementary figure 22 shows the water content in the as-received solvents used in this study (CHP, NMP and IPA) immediately after opening a fresh bottle and over the course of ~160 h. The straight lines are a guide for the eye, as we rather expect the data to follow an exponential increase opposed to a linear increase. It is interesting to note that the water content in CHP is even higher than in IPA. This emphasises that the slower degradation in CHP cannot be attributed to a lower water content in the solvent, but is a result of basal plane protection

The role of water in the degradation, was analysed through purposeful addition of varying proportions of water to std-BP, S-BP and L-BP samples whilst measuring the optical density of the nanosheets as a function of time as described in the main manuscript. All degradation plots of std-BP, S-BP and L-BP are shown in supplementary figure 23 and the kinetic data is summarised in supplementary table 4. As shown in the main manuscripts, time constants decrease with increasing water content accompanied with an increased proportion of reacted BP. Notably, the L-BP showed slower reaction rates compared to std-BP and in particular S-BP again suggesting degradation to start from the edge. It is therefore also clear that water/O_2 plays an important role in the degradation of the nanosheets through increasing the rate of degradation. Unfortunately, since oxygen is highly soluble in water, we could not distinguish between the role of oxygen and water in these experiments. However, we did not observe different degradation kinetics when additionally bubbling oxygen through the dispersion.

We also note that this degradation is accompanied with a change in pH which typically decreases from 10 to 8. However, a quantification was not possible, as the pH determination

in CHP as organic solvent is not accurate. Nonetheless, this suggest the reaction products to be phosphorous and/or phosphoric acid (consistent with XPS).

Supplementary Note 2: Modelling of the degradation

<u>Energy profile of the early stages of the reaction starting from the edge and the basal plane</u>

Whether occurring at the edge or in the middle of a BP nanosheet, the degradation is a multistep process, where subsequent adsorption and splitting processes of three water molecules eventually lead to the formation and release of PH_3 and PO_3H_3 molecular products. The reaction investigated is in general: (BP_{2VAC} is a BP flake with two vacancies)

$BP + 3H_2O \rightarrow BP_{2VAC} + PH_3 + H_3PO_3$.

The BP oxidation i) starts with a water molecule approaching the flake and ending up in a physisorbed state (Supplementary figure 34). Water adsorption both in the middle of the flake and at the edge is a slightly exothermic process, with adsorption energies of 0.21 eV and 0.12 eV[1], respectively for the middle and the edge (Supplementary figure 34, step 2). (ii.) The reaction then proceeds with water splitting. The hydroxyl group and the H atom from water chemisorb on two neighbouring P atoms. Importantly, in the middle of the nanosheet the water splitting is endothermic (by 1.38 eV), whilst it is exothermic by 0.80 eV at the edge (Supplementary figure 34, step 3). This further confirms that the oxidation is thermodynamically favourable only at the edge.

After the completed reaction to release PH_3 and H_3PO_3, two P vacancies per BP unit are formed resulting in either a big hole in the centre of the surface (Supplementary figure 35) or a defective edge. Different final configurations for defective BP in the centre and at its edge (with P vacancies in different configurations) have been compared. Insets 4 show the most stable final states. It is clear that the overall reaction energy is negative when the reaction starts at and proceeds from the edge, while it is positive in case of the scenario at the centre.

It is interesting to note that there is a relevant difference between overall reaction energies for the process in the centre of PB, when calculated at PBE level[23] (endothermic by 0.99 eV) or corrected by vdW [4] (endothermic by 0.22 eV).This shows the importance of including vdW contribution in the description of BP defective structures and their interaction with water.

Finally, possible inclusion of rotational and translational entropic contributions to the reaction energies should not be sufficient to change the sign of reaction energy for the reaction at the edge, and should make the reaction in the centre even more endothermic, as the number of free molecules reduces from three water molecules to two reaction products.

---

[1] The adsorption energy in this case is evaluated as $\Delta E=[E(BP)+ E(H_2O)] -E(BP+WAT)$ representing the difference between the energy of the configuration where a water molecule and a BP nanosheet are at infinite distance and that of a flake with water adsorbed (on the surface or at the edge)

Reaction starting from defective BP

We also studied the degradation process when water attacks a defective nanosheet. This is an important test, as high local energies in the sonication process may provide enough activation for the degradation to occur in the basal plane. We have therefore evaluated the energy of the following reaction

$BP_{2VAC} + 3H_2O \rightarrow BP_{4VAC} + PH_3 + H_3PO_3$.

This process originates from a nanosheet incorporating two P vacancies and produces a defective layer with 4 P vacancies, i.e. a well discernible hole in the basal plane (Supplementary figure 36) in addition to the other reaction products. The reaction is again endothermic by approximately $\Delta E=0.26$ eV, which is very similar to the value for the reaction started on the ideal monolayer. Thus, such a process is not expected to occur spontaneously. This is consistent with the intact lattice observed experimentally (see figures 1H and 1H in the main manuscript), which did not show evidence for holes.

Supplementary Note 3: Experimental evaluation of the stoichiometry of the reagents in the BP degradation

Tracking extinction spectra over time can also be used to simultaneous follow the consumption of water and BP. With knowledge of the extinction/absorbance coefficients, this can in principle be used to quantify the reacted concentration of water and BP, respectively. It would of course be beneficial to also use absorbance spectra for this analysis. However, the spectral range of our integrating sphere set up is limited so that we had to estimate the concentrations from extinction spectra.

To do this, we first determine the extinction coefficient of water in CHP. Supplementary figure 37A shows extinction spectra of water added to anhydrous CHP showing peaks from the water at higher wavelengths centred at around 1430 nm. Supplementary figure 37B shows a plot of the extinction at 1430 nm as a function of water concentration in the CHP. The extinction coefficient was determined as 37.2 mM^{-1} m^{-1}. A difficulty in the analysis of the water extinction in the BP samples arises from the fact that BP also contributes to the intensity at 1430 nm. This is best observed in the case of the std-BP GB sample (Supplementary figure 37C). This is particularly problematic because the BP background will change in intensity as a function of time due to the degradation. We therefore estimate the BP background in each measurement as follows: At 1100 nm, the contribution from water to the extinction spectra is negligible. Therefore, we can attributed the intensity at this spectral position to only BP. From the spectrum of the std-BP GB sample with negligible water content, we can estimate the contribution of BP to the extinction at 1430 nm (used to determine the water content) as ~ 0.68 Ext_{1100}. Furthermore, when analysing the spectra of the std-BP GB samples after various time intervals of exposure to ambient conditions, we see an increase in the water extinction. This means that significantly more water is taken up from the environment by the hygroscopic solvent CHP than reacted with BP. This is because degradation time constants of BP are high for low water contents. On the contrary, we observe a clear fall of the water (and BP) extinction with reaction time when a sample is studied where water was initially added (10 vol%) to accelerate the reaction (Supplementary figure 37D).

The corrected water extinction expressed as Ext_{1430} - 0.68 Ext_{1100} is plotted as a function of time for three samples in supplementary figure 37E (std-BP GB left under ambient and after addition of water to yield initial water contents of 3 and 10 vol% respectively). Clearly, the water content rises exponentially in the GB reference sample and decreases for both samples where water was initially added. We use our water extinction coefficient at 1430 nm

to first determine the concentration of water in the samples as a function of time. This can also be expressed as concentration of reacted water as a function of time which is given by the initial concentration of water – the measured concentration of water at a given reaction time – the water uptake as estimated from the reference sample. The resultant data of reacted water is plotted as a function of time in Supplementary figure 37F.

Similarly, as shown throughout the manuscript, the concentration of BP also drops as a function of time as reflected by the exponential decay curves of extinction vs time. The data of the corresponding samples is shown in Supplementary figure 37G. These can be converted to plots of reacted BP as a function of time (Supplementary figure 37H). By dividing the reacted concentration of water by the reacted concentration of BP, we can estimate the stoichiometry ratio of water/BP in the degradation reaction as shown in the main manuscript figure 6C.

The as-obtained water/BP stoichiometry ratio is overestimated compared to the theoretical expectation. It is therefore important to identify the main source of uncertainty. Of course, the necessity to measure extinction rather than absorbance may have an impact on the accuracy of the data analysis. However, as shown in Supplementary figure 20, the error appears to be negligible.

The greatest source of error is certainly presented in the accuracy of the extinction coefficients used to convert extinction into concentration. These values can have errors of up to ±5 % in the case of molecules and are even larger in the case of nanomaterials. This is related to the fact that it is difficult to accurately determine the mass of dispersed nanosheets after filtration and weighing. This is particularly difficult in the case of BP, as the filtered films cannot be extensively washed with low boiling point solvents to remove the CHP more completely due to the then more pronounced degradation occurring at shorter time constants. The samples can also not be extensively dried for long periods of time at elevated temperature to remove all of the residual CHP. It is therefore likely that our mass after filtration and weighing is overestimated which means the extinction coefficient of the BP is probably underestimated. To test whether this would shift the experimentally determined stoichiometry ratio in the right direction, we have reanalysed the data under the assumption that our extinction coefficient of BP is underestimated by 15%. The resultant graphs are presented in supplementary figure 38revealing that this has indeed an impact on the experimentally determined stoichiometry ratio. In fact the more reliable data points with the higher water concentration are close to a stoichiometry ratio of 1.5.

Supplementary Note 4: Calculated mechanical properties of black phosphorus

Calculated bulk and in-plane mechanical properties of black phosphorus

The mechanical properties of bulk phosphorus (BP) were calculated by means of Kohn-Sham density-functional theory (DFT)[24] as implemented in the QuantumEspresso package,[25] using the Perdew-Burke-Ernzerhof (PBE) exchange-correlation functional.[23] An ultrasoft pseudopotential was used to represent the core electrons, while van der Waals interactions were treated using the B97-D empirical dispersion correction functional,[26] which includes damped pairwise dispersion corrections between atoms of the form r^{-6}. Convergence was achieved to within 1 meV per atom, with respect to plane-wave energy cutoff, kinetic energy cutoff and Brillouin zone sampling. Full geometry optimisation yielded the lattice parameters shown in supplementary table 5, which compared well to both literature[18] and experiment.[19]

Our computed elastic constants for BP are shown in supplementary table 6. In order to compute these constants, the relaxed unit cell was subjected to normal strain parallel to the *x*, *y*, and *z* directions, and shear strain about the axes perpendicular to the *yz*, *xz* and *xy* planes, in the range [-12%, +12%] and in intervals of 2%. The elastic tensor elements, shown in supplementary table 6, were extracted from the linear coefficients of polynomials fitted to the resulting stress-strain profiles, and again compare reasonably well with literature.[18]

The Voigt-Reuss-Hill model[27] was used to estimate isotropically averaged mechanical properties of BP, based on our computed stiffness tensor elements. The Voigt estimate assumes a constant strain throughout the material, and is computed by averaging the stresses and strains defining the stiffness tensor C over solid angles, and yields an upper-bound to the isotropically averaged Young's modulus. The Reuss model, on the other hand, assumes constant stress and likewise averages the compliance tensor $\mathrm{S} = \mathrm{C}^{-1}$ yielding a lower-bound. The Voigt-Reuss-Hill estimate[28] is defined as the average of the two, and provides reasonable results for many solids.[29-31] The results are shown in supplementary table 7 in comparison with those provided by Ref [18].

Two-dimensional estimates of elastic properties

Having verified the agreement of our computed isotropically averaged elastic constants for BP with previous results, we now turn to two-dimensional averages used in simulating the Young's modulus of a composite of phosphorus nanosheet and rubber, as discussed in the Methods section and shown in Fig. 7G. In this composite material, the reinforcing BP nanosheets are assumed to form a planar sediment, based on knowledge of similar materials[32] and enhancement of the elastic modulus is measured in this plane. In order to simulate this reinforcement, therefore, we require in-plane, two-dimensional averages of the elastic properties of BP, rather than fully isotropically averaged quantities. In this case, due to weak van der Waals bonds between layers, moreover, we may neglect the Poisson's ratios related to out-of-plane strain, and the $6x6$ stiffness tensor C reduces to a $3x3$ matrix of the form

$$C_{2D} = \begin{pmatrix} c_{11} & c_{12} & 0 \\ c_{12} & c_{22} & 0 \\ 0 & 0 & c_{66} \end{pmatrix} \quad (1)$$

Defining the two-dimensional compliance tensor $S_{2D} = C_{2D}^{-1}$ with elements s_{ij} we calculated the angular dependence of the two-dimensional Young's modulus $Y(\theta)$, shear modulus $G(\theta)$, and Poisson's ratio $\nu(\theta)$ using the following equations:[33, 34]

$$\mathrm{Y_{BP}}(\theta) = (\mathrm{s}_{11}\cos^4\theta + \mathrm{s}_{22}\sin^4\theta + (2\mathrm{s}_{12} + \mathrm{s}_{66})\cos^2\theta\sin^2\theta)^{-1} \quad (2)$$

$$G(\theta) = \left((4s_{11} + 4s_{22} - 8s_{12} - 2s_{66})\cos^2\theta\sin^2\theta + s_{66}(\cos^4\theta + \sin^4\theta)\right)^{-1} \quad (3)$$

$$\nu(\theta) = -\frac{s_{12}(\cos^4\theta + \sin^4\theta) + (s_{11} + s_{22} - s_{66})\cos^2\theta\sin^2\theta}{\mathrm{s}_{11}\cos^4\theta + \mathrm{s}_{22}\sin^4\theta + (2\mathrm{s}_{12} + \mathrm{s}_{66})\cos^2\theta\sin^2\theta} \quad (4)$$

The results of which are shown in Fig. 7J. We also computed quasi-Voigt (Y_V) and quasi-Reuss (Y_R) values of the two-dimensional Young's modulus by integrating $Y(\theta)$ arithmetically and harmonically, *via*

$$Y_v = \frac{1}{2\pi}\int_0^{2\pi} Y_{BP}(\theta)\, d\theta\,, \quad (5)$$

$$Y_R = \left(\frac{1}{2\pi}\int_0^{2\pi}\frac{1}{Y_{BP}(\theta)}\, d\theta\right)^{-1}\,, \quad (6)$$

$$\langle Y_{BP}\rangle = \frac{1}{2}(Y_V + Y_R) \quad (7)$$

This yielded results of $Y_V = 110.45$ GPa, $Y_R = 84.24$ GPa and a two-dimensional quasi-Voigt-Reuss-Hill average of $\langle Y_{BP}\rangle = 97.35$ GPa which is also shown in Fig. 7J for comparison with $Y(\theta)$.

Comparison of computed effective medium and experimental Young's moduli for a black phosphorus reinforced composite

The in-plane average of the BP Young's modulus, $\langle Y_{BP}\rangle$ in conjunction with the measured modulus of the polymer matrix at 0% BP loading, Y_{poly} was used to simulate the modulus of the BP reinforced rubber composite, as discussed in the Methods section and shown in Fig. 7G. For this, we assume loading in the plane of BP sedimentation, hence neglecting out-of-plane response, and random orientation of BP flakes in the plane. As such, an effective medium model assuming loading parallel to extension, and hence constant strain, such as the simple rule of mixtures estimate, defined by

$$Y_{comp} = \langle Y_{BP}\rangle V_f + Y_{poly}\left(1 - V_f\right) \qquad (8)$$

where V_f is the volume fraction of BP nanosheets, may be expected to provide a reasonable approximation for the composite Young's modulus. As shown in Fig.6.G, this model fits the experimental data within the large error bars inherited from the experimental uncertainty in Y_{poly} The uncertainly in the computed elastic constants, which broadens the error bars with increasing concentration of BP nanosheets, is defined by $\Delta\langle Y_{BP}\rangle = \frac{1}{2}(Y_V - Y_R)$.

Supplementary Methods

Materials

Black phosphorus crystals were purchased from Smart Elements (purity 99.998%); N-cyclohexyl-2-pyrrolidone (CAS 6837-24-7) and 2-Proponal (CAS 67-63-0) and all other chemicals were purchased from Sigma Aldrich. All reagents were used without any further purification.

Sample preparation

Dispersion of FL-BP nanosheets

Pieces of black phosphorus crystal were lightly ground, using a pestle and mortar, to give a powder. This powder was subsequently added to CHP (80 mL) in a 100 mL open top beaker to yield an initial concentration of 2 g/L. The beaker was connected to an external cooling system that allowed for cooled water (5 °C) to flow around the dispersion during sonication. This solution was sonicated for 5 hours at 60% amplitude with a horn-probe sonic tip (VibraCell CVX, 750W). The sonication was pulsed for 6s on 2s off to avoid any damage to the processor and reduce solvent heating. These conditions produce a standard stock dispersion which is subjected to further centrifugal optimisation. These samples were subsequently centrifuged at 1krpm for 180 min and subjected to absorbance spectroscopy.

Centrifugation conditions

Aliquots of the stock dispersion (25 mL) were placed in vials (28 mL) and centrifuged at 1,000 rpm (106 *g*) for time periods varying from 5 to 240 min in a Hettich Mikro 220R centrifuge equipped with a fixed-angle rotor 1016. The supernatant was decanted and subjected to absorbance spectroscopy. Centrifugation methodologies as previously shown,[1] based on sedimentation rates following an exponential decay are also observed here. Dispersions are deemed stable for centrifugation times > $2t_1$ after which >85% of the sedimenting material has been removed (NB, depending on the rpm, the sedimenting material will consist of unexfoliated crystallites and large nanosheets). Here, t_1= 90 min. The supernatant with centrifugation conditions 1,000 rpm for 180 min was denoted standard sample (std-BP). The std-BP was subsequently separated into small and large stable nanosheets.

For size selection purposes, aliquots of std-BP dispersion were subjected to an additional centrifugation of 5 krpm (2,660 *g*) for 120 min. The supernatant was decanted and characterised as S-BP while the sediment was re-dispersed in fresh CHP and characterised as L-BP. This process yielded three stable nanosheets dispersions, std-BP, S-BP and L-BP each subjected to further characterisation.

Nanosheets size selection controlled centrifugation with subsequently increasing rotation speeds

The sediment after 2 krpm (426 *g*, 2 h) was discarded, while the supernatant was subjected to further centrifugation at 3 krpm (958 *g*, 2 h). The sediment was collected in fresh solvent, while the supernatant was subjected to further centrifugation at 4 krpm (1,702 g, 2 h). Again, the sediment was collected and the supernatant centrifuged at high rpm. This procedure was repeated for 5 krpm (2,660 *g*, 2 h), 10 krpm (10,170 *g*, 2 h) and 16 krpm (25,000 *g*, 2 h) to yield samples with decreasing sizes in the respective sediments. A Hettich Mikro 220R centrifuge equipped with a fixed-angle rotor 1016 and sample aliquots of 25 mL was used for rotations up to 5 krpm. For the high speed centrifugations, 1.5 mL vials and a rotor 1195-A was used.

Exfoliation under inert conditions and sample for PL

CHP solvent (Sigma-Aldrich, 99%) was dried by allowing it to stand over a desiccant (4 Å molecular sieves from Sigma-Aldrich predried at 300 °C under Argon atmosphere for 72 h) under argon for 96 h. A coulometric Karl Fischer titrator was used to determine the water content. Residual traces of oxygen were removed by pump freeze treatment (five iterative steps) before introducing the CHP into the argon-filled Labmaster sp glovebox (MBraun), equipped with a gas filter to remove solvents and an argon cooling systems, with an oxygen content <0.1 ppm and a water content <0.1 ppm.

Upon introduction to the glove box the sample was stored in a dark bottle until the experiment was completed. Black phosphorus crystal was ground inside the glove box and used as a crushed powder. This was then added to the dried CHP solvent to form a mixture of BP/CHP (BP concentration 0.5 g/L, 15 mL CHP). The sample was sonicated in situ at 25% amplitude with a pulse rate of 2s on 2s off, for a total of 2 hours in a Bandelin Sonoplus 3100. This sonication process was repeated three times to build up a stock dispersion. After sonication the samples were combined and subjected to centrifugation at 106 g for 3 hours in sealed centrifugation vials outside of the glove box. The sample was reintroduced to the glove

box and the supernatant was decanted. The supernatant was diluted, with dry CHP (29 ppm of water), to a similar optical density as the previous stability studies. It is important to note that unlike previous solvents which have been used to exfoliate materials in our glove box it is common during the sonication to observe an increase in the oxygen levels (~3-4ppm) of the glove box, however in the case of CHP we did not observe such increase.

For the degradation study, the glove box sample was measured by storing a stock solution of the sample in the glove box and removing 1.5ml to be subjected to extinction spectroscopy (Lambda 1050, see below). After the measurement, this aliquot was discarded. For the entire study the glove box sample was wrapped in aluminium foil to avoid light exposure and stored under conditions with water and oxygen contents of < 0.1 ppm.

For the PL measurement, the std-BP GB sample was further treated as follows: to increase the population of few-layered species for the PL measurement, this std-BP GB sample was centrifuged for 16 h at 25 g. The sediment was discarded and the supernatant centrifuged again at 710 g for 180 min. All solvent transfer was carried out in the glovebox. The PL spectra shown in the main manuscript were acquired from the supernatant of the final centrifugation step at 710 *g*. The three sizes (small, medium, large-BP-PL) shown in the SI are the following: small-BP-PL: supernatant after the final centrifugation step at 710 *g*, large-BP-PL: sediment after the final centrifugation step at 710 *g*, medium-BP-PL: dispersion before this final centrifugation step.

Karl Fisher titrations of solvents

To determine the water content of solvents over the course of the study (~160 h) we used the “Karl Fischer titration” a well-known water analysis method (Metrohm USA Inc., http://www.metrohmusa.com/Service/FAQ/Titration/Coulo.html). The Coulometric Karl Fischer reaction involves adding a known mass of sample to the reaction vessel containing a base, alcohol and iodine. A current change in the electrode is noted which relates to a chemical change occurring in the vessel.

Composite preparation

Filler Solution Preparation: Previously prepared nanofiller (FL-BP) dispersion in N-Cyclohexyl-2-pyrrolidone, centrifuged between 1 krpm for 180min and 3 krpm for 120 min and redispersed in fresh CHP solvent, was filtered onto a Polyester Membrane Filter (0.2 micron) of known mass. The membrane was dried in a vacuum oven at 100°C for 2 h and the mass of the membrane remeasured to attain the mass of the filtered nanofiller. The nanofiller

was redispersed by bath sonication (Branson 1510 Model 45 kHz) in a 65:35 tetrahydrofuran (THF), chloroform solvent mixture. Polymer Solution Preparation: Poly (vinyl chloride) (PVC) supplied by Sigma Aldrich in granular form (~48000 MW) was dissolved in a solvent mixture (65:35 tetrahydrofuran, chloroform) using a reflux setup at 30°C to form a 30 g/L solution. Film Preparation: A range of FL-BP/PVC/THF/Chloroform dispersions (from 0 to 0.0074 Vf) were made by adding the FL-BP/THF/ Chloroform filler solution to the PVC/THF/ Chloroform solution with varying increments of loading. These solutions were of constant mass (150 mg of FL-BP & polymer) and constant volume (5 mL of FL-BP, polymer & solvent). These samples were sonicated in the same bath as before for 1 h to homogenise after the blending of the solutions. The homogenised solution mixtures were then dropcast into 5cm x 5cm x 1cm Teflon trays. The samples were then placed in a vacuum oven for 4 hours at 40 °C under no vacuum to form composite films by removal of the solvent through evaporation. The films were then kept overnight (~17 h) at 50°C under full vacuum to ensure that the solvent is completely removed and to protect the filler material from decomposition before testing.

Gas sensing

Gas sensing was conducted on the FL-BP (std-BP) prepared in CHP and then subsequently transferred into 2-proponal to facilitate filtration onto a nitrocellulose membrane. Following filtration the film was allowed to dry under vacuum conditions. The FL-BP film was cut into 10×2mm rectangular pieces. These were then transferred onto silicon dioxide wafer while the nitrocellulose membrane was dissolved using the Wu et al transfer method.[35]

For gas sensing, gold electrodes were sputtered on top of an adhesion layer of nickel (Ni/Au = 30/70 nm) using a metal shadow mask, which has 2 mm wide and 200 um long channel. All devices were loaded in a gas sensing chamber and annealed at 100 °C for 1 hour to remove residues and adsorbates on the surface. The gas sensing chamber was kept at room temperature at pressure 10 Torr, with a 100 sccm flow of the NH_3 mixtures. The resistance change of five devices upon interval gas exposure was simultaneously measured using a Keithley model 2612A SourceMeter and a Keithley 3706 System Switch at a constant bias voltage of 1 V. The initial resistance and RMS noise were calculated from the first 500 data points, approximately 2 min, before the first gas injection. NH_3 for 2 min and pure N_2 for 5 min were periodically introduced to record sensor response and recover, respectively.

Characterisation

Optical extinction and absorbance was measured on a Perkin Elmer 650 spectrometer in quartz cuvettes with a path length of 0.4 cm. To distinguish between contributions from scattering and absorbance to the extinction spectra, dispersions were measured in an integrating sphere using a home-built sample holder to place the cuvette in the centre of the sphere (NB cuvettes need to be transparent to all sides). The absorbance spectrum is obtained from the measurement inside the sphere. A second measurement on each dispersion was performed outside the sphere to obtain the extinction spectrum. This allows calculation of the scattering spectrum (extinction-absorbance). The experiments to track both water and BP degradations were performed on a Perkin Elmer Lambda 1050 spectrometer in extinction. During the course of the degradation experiments, all samples were stored under ambient conditions in a box with an open lid and thus exposed to similar light intensities.

Bright field transmission electron microscopy imaging was performed using a JEOL 2100, operated at 200 kV while HRTEM was conducted on a FEI Titan TEM (300 kV). Holey carbon grids (400 mesh) were purchased from Agar Scientific and prepared by diluting dispersion to a low concentration and drop casting onto a grid placed on a filter membrane to wick away excess solvent. Statistical analysis was performed of the flake dimensions by measuring the longest axis of the nanosheet and assigning it “length, L”. Aberration-corrected scanning transmission microscopy (STEM) images were taken using a Nion Ultrasteme 100 (cold filed emission gun (FEG)) at the SuperSTEM Laboratory in Daresbury, UK. The suspended FL-BP was dropped onto lacey carbon coated copper TEM grids as described above. The samples were then prebaked at 120^{0}C in a vacuum overnight. The images were recorded using a 100 kV acceleration voltage using a high field annular dark field detector (HAADF) and low-pass bright field imaging.

Atomic force microscopy (AFM) was carried out on a Veeco Nanoscope-IIIa (Digital Instruments) system equipped with a E-head (13 μm scanner) in tapping mode after depositing a drop of the dispersion (10 μL) on a pre-heated (150 °C) Si/SiO_2 wafer with an oxide layer of 300 nm. Typical image sizes were 3-10 μm at scan rates of 0.4-0.6 Hz. To facilitate deposition, the FL-BP sample in CHP was transferred to IPA by centrifugation at 15 krpm (22,640 g, rotor 1195-A), removal of CHP supernatant and reagitation of the sediment in IPA.

Raman spectroscopy on individual flakes was performed using a Horiba Jobin Yvon LabRAM HR800 with 633 nm excitation laser in air under ambient conditions. The Raman emission was collected by 100× objective lens (N.A. = 0.8) and dispersed by 1800 gr/mm. To

avoid sample heating we carried out all Raman experiments at 10% of maximum laser power (<2 mW) when measuring on Si/SiO_2 wafers and 0.1% (<0.02 mW). Relocalisation was achieved by using the optical contrast of nanomaterials deposited on opaque bilayered substrates.

X-ray Photoelectron Spectroscopy was performed under ultra-high vacuum conditions ($<5\times10^{-10}$ mbar), using monochromated Al Kα X-rays (1486.6 eV) from an Omicron XM1000 MkII X-ray source and an Omicron EA125 energy analyser. An Omicron CN10 electron flood gun was used for charge compensation and the binding energy scale was referenced to the adventitious carbon 1s core-level at 284.8 eV. Core-level regions were recorded at an analyser pass energy of 15 eV and with slit widths of 6 mm (entry) and 3 mm x 10 mm (exit), resulting in an instrumental resolution of 0.48 eV. After subtraction of a Shirley background, the core-level spectra were fitted with Gaussian-Lorentzian line shapes and using Marquardt's algorithm. Samples were prepared by filtering the dispersion onto alumina membranes. Prior to XPS, the top layer of the film which was exposed to ambient conditions was peeled off with scotch tape.

Photoluminescence (PL) was acquired on a Horiba Scientific Fluorolog-3 system equipped with 450 W Xe halogen lamp, double monochromator in excitation (grating 600 lines/mm blazed at 500 nm) and emission (grating 100 lines/mm blazed at 780 nm) and a nitrogen cooled InGaS diode array detector (Symphony iHR 320). Spectra were obtained at 5°C in front face mode to avoid potential contributions from reabsorption effects. Excitation and emission band widths were typically 10 nm and integration times 8 s for the low wavelength emission and 15 s for the higher wavelength features. The BP dispersions were diluted to an optical density of 0.4 cm^{-1} at 540 nm. For the spectral region of 550-1300 nm in emission, a 550 nm cut-off filter on the emission side was used. To resolve the lower energy features, a 830 nm cut-off filter was placed on the emission side.

For mechanical testing, the polymer composite films were cut into 2.25 mm strips and then tested on a Zwick Roell tensile tester with a 100N load cell at a strain rate of 10 mm/min.

NLO: An open-aperture Z-scan system was used to study the ultrafast nonlinear optical properties of the FL-BP (std-BP) and graphene dispersions. This measures the total transmittance through a sample as a function of incident laser intensity, while the sample is sequentially moved through the focus of a lens (along the z-axis).[36, 37] All experiments were performed with 340 fs pulses from a mode-locked fiber laser, which was operated at 1030 nm and its second harmonic, 515 nm, with a pulse repetition rate of 10 KHz. All dispersion samples were tested in quartz cuvettes with 1 mm pathlength.

For gas sensing, gold electrodes were sputtered on top of an adhesion layer of nickel (Ni/Au = 30/70 nm) using a metal shadow mask, which has 2 mm wide and 200 um long channel. All devices were loaded in a gas sensing chamber and annealed at 100 °C for 1 hour to remove residues and adsorbates on the surface. The gas sensing chamber was kept at room temperature at pressure 10 Torr, with a 100 sccm flow of the NH_3 and N_2 mixtures. The resistance change of five devices upon interval gas exposure was simultaneously measured using a Keithley model 2612A SourceMeter and a Keithley 3706 System Switch at a constant bias voltage of 1 V. The initial resistance and RMS noise were calculated from the first 500 data points, approximately 2 min, before the first gas injection. NH_3 for 2 min and pure N_2 for 5 min were periodically introduced to record sensor response and recover, respectively.

Supplementary References

1. Hanlon, D.; Backes, C.; Higgins, T. M.; Hughes, M.; O'Neill, A.; King, P.; McEvoy, N.; Duesberg, G. S.; Mendoza Sanchez, B.; Pettersson, H.; Nicolosi, V.; Coleman, J. N., Production of Molybdenum Trioxide Nanosheets by Liquid Exfoliation and Their Application in High-Performance Supercapacitors. *Chem. Mater.* **2014,** *26* (4), 1751-1763.
2. Backes, C.; Smith, R. J.; McEvoy, N.; Berner, N. C.; McCloskey, D.; Nerl, H. C.; O'Neill, A.; King, P. J.; Higgins, T.; Hanlon, D.; Scheuschner, N.; Maultzsch, J.; Houben, L.; Duesberg, G. S.; Donegan, J. F.; Nicolosi, V.; Coleman, J. N., Edge and Confinement Effects Allow in situ Measurement of Size and Thickness of Liquid-Exfoliated Nanosheets. *Nat. Commun.* **2014,** (*5)*, 4576.
3. Paton, K. R.; Varrla, E.; Backes, C.; Smith, R. J.; Khan, U.; O'Neill, A.; Boland, C.; Lotya, M.; Istrate, O. M.; King, P.; Higgins, T.; Barwich, S.; May, P.; Puczkarski, P.; Ahmed, I.; Moebius, M.; Pettersson, H.; Long, E.; Coelho, J.; O'Brien, S. E.; McGuire, E. K.; Sanchez, B. M.; Duesberg, G. S.; McEvoy, N.; Pennycook, T. J.; Downing, C.; Crossley, A.; Nicolosi, V.; Coleman, J. N., Scalable production of large quantities of defect-free few-layer graphene by shear exfoliation in liquids. *Nat. Mater.* **2014,** *13* (6), 624-630.
4. Harvey, A.; Backes, C.; Gholamvand, Z.; Hanlon, D.; McAteer, D.; Nerl, H. C.; McGuire, E.; Seral-Ascaso, A.; Ramasse, Q. M.; McEvoy, N.; Winters, S.; Berner, N. C.; McCloskey, D.; Donegan, J. F.; Duesberg, G. S.; Nicolosi, V.; Coleman, J. N., Preparation of Gallium Sulfide Nanosheets by Liquid Exfoliation and Their Application As Hydrogen Evolution Catalysts. *Chem. Mater.* **2015,** *27* (9), 3483-3493.
5. Ridings, C.; Warr, G. G.; Andersson, G. G., Composition of the outermost layer and concentration depth profiles of ammonium nitrate ionic liquid surfaces. *Phys. Chem. Chem. Phys.* **2012,** *14* (46), 16088-16095.
6. Nemes-Incze, P.; Osváth, Z.; Kamarás, K.; Biró, L. P., Anomalies in thickness measurements of graphene and few layer graphite crystals by tapping mode atomic force microscopy. *Carbon* **2008,** *46* (11), 1435-1442.
7. Favron, A.; Gaufres, E.; Fossard, F.; Phaneuf-Lheureux, A.-L.; Tang, N. Y. W.; Levesque, P. L.; Loiseau, A.; Leonelli, R.; Francoeur, S.; Martel, R., Photooxidation and quantum confinement effects in exfoliated black phosphorus. *Nat. Mater.* **2015,** *14* (8), 826-832.

8. Rodin, A. S.; Carvalho, A.; Neto, A. H. C., Excitons in anisotropic two-dimensional semiconducting crystals. *Phys. Rev. B.* **2014,** *90* (7), 075429.

9. Qiao, J.; Kong, X.; Hu, Z.-X.; Yang, F.; Ji, W., High-mobility transport anisotropy and linear dichroism in few-layer black phosphorus. *Nat Commun* **2014,** *5*, 5475.

10. Currie, L. A., Nomenclature in evaluation of analytical methods including detection and quantification capabilities (IUPAC recommendations 1995). *Pure Appl. Chem.* **1995,** *67* (10), 1699-1723.

11. Kou, L.; Frauenheim, T.; Chen, C., Phosphorene as a Superior Gas Sensor: Selective Adsorption and Distinct I–V Response. *J. Phys. Chem. Lett.* **2014,** *5* (15), 2675-2681.

12. Leenaerts, O.; Partoens, B.; Peeters, F. M., Adsorption of H_2O, NH_3 , CO, NO_2 , and NO on graphene: A first-principles study. *Phys. Rev. B.* **2008,** *77* (12).

13. Zhao, S.; Xue, J.; Kang, W., Gas adsorption on MoS2 monolayer from first-principles calculations. *Chem. Phys. Lett.* **2014,** *595*, 35-42.

14. Lee, K.; Gatensby, R.; McEvoy, N.; Hallam, T.; Duesberg, G. S., High-Performance Sensors Based on Molybdenum Disulfide Thin Films. *Adv. Mater.* **2013,** *25* (46), 6699-6702.

15. O'Brien, M.; Lee, K.; Morrish, R.; Berner, N. C.; McEvoy, N.; Wolden, C. A.; Duesberg, G. S., Plasma assisted synthesis of WS2 for gas sensing applications. *Chem. Phys. Lett.* **2014,** *615*, 6-10.

16. Schedin, F.; Geim, A. K.; Morozov, S. V.; Hill, E. W.; Blake, P.; Katsnelson, M. I.; Novoselov, K. S., Detection of individual gas molecules adsorbed on graphene. *Nat. Mater.* **2007,** *6* (9), 652-655.

17. Li, J.; Lu, Y. J.; Ye, Q.; Cinke, M.; Han, J.; Meyyappan, M., Carbon nanotube sensors for gas and organic vapor detection. *Nano Lett.* **2003,** *3* (7), 929-933.

18. Appalakondaiah, S.; Vaitheeswaran, G.; Lebègue, S.; Christensen, N. E.; Svane, A., Effect of van der Waals interactions on the structural and elastic properties of black phosphorus. *Phys. Rev. B.* **2012,** *86* (3), 035105.

19. Kôzuki, Y.; Hanayama, Y.; Kimura, M.; Nishitake, T.; Endo, S., Measurement of Ultrasound Velocity in the Single Crystal of Black Phosphorus up to 3.3 GPa Gas Pressure. *J. Phys. Soc. Jpn.* **1991,** *60* (5), 1612-1618.

20. Castellanos-Gomez, A.; Vicarelli, L.; Prada, E.; Island, J. O.; Narasimha-Acharya, K. L.; Blanter, S. I.; Groenendijk, D. J.; Buscema, M.; Steele, G. A.; Alvarez, J. V.; Zandbergen, H. W.; Palacios, J. J.; Zant, H. S. J. v. d., Isolation and characterization of few-layer black phosphorus. *2D Mater.* **2014,** *1* (2), 025001.

21. Wood, J. D.; Wells, S. A.; Jariwala, D.; Chen, K.-S.; Cho, E.; Sangwan, V. K.; Liu, X.; Lauhon, L. J.; Marks, T. J.; Hersam, M. C., Effective Passivation of Exfoliated Black Phosphorus Transistors against Ambient Degradation. *Nano Lett*. **2014**, 14 (12), 6964-6970.

22. Li, L.; Yu, Y.; Ye, G. J.; Ge, Q.; Ou, X.; Wu, H.; Feng, D.; Chen, X. H.; Zhang, Y., Black phosphorus field-effect transistors. *Nat Nano* **2014,** *9* (5), 372-377.

23. Perdew, J. P.; Burke, K.; Ernzerhof, M., Generalized Gradient Approximation Made Simple. *Phys. Rev. Lett* **1996,** *77* (18), 3865-3868.

24. Hohenberg, P.; Kohn, W., Inhomogeneous Electron Gas. *Phys. Rev.* **1964,** *136* (3B), 864-871.

25. Giannozzi, P.; Baroni, S.; Bonini, N.; Calandra, M.; Car, R.; Cavazzoni, C.; Ceresoli, D.; Chiarotti, G. L.; Cococcioni, M.; Dabo, I.; Corso, A. D.; Gironcoli, S. d.; Fabris, S.; Fratesi, G.; Gebauer, R.; Gerstmann, U.; Gougoussis, C.; Kokalj, A.; Lazzeri, M.; Martin-Samos, L.; Marzari, N.; Mauri, F.; Mazzarello, R.; Paolini, S.; Pasquarello, A.; Paulatto, L.; Sbraccia, C.; Scandolo, S.; Sclauzero, G.; Seitsonen, A. P.; Smogunov, A.; Umar, P.; Wentzcovitch, R. M., QUANTUM ESPRESSO: a modular and open-source software project for quantum simulations of materials. *J. Phys.: Condens. Matter* **2009** *21* (39), 395502

26. Grimme, S., Semiempirical GGA-type density functional constructed with a long-range dispersion correction. *J. Comput. Chem.* **2006,** *27* (15), 1787-1799.

27. Toonder, J. M. J. d.; Dommelen, J. A. W. v.; Baaijens, F. P. T., The relation between single crystal elasticity and the effective elastic behaviour of polycrystalline materials: theory, measurement and computation. *Model. Simul. Mater. Sci. Eng*. **1999,** *7* (6), 909.

28. Hill, R., The Elastic Behaviour of a Crystalline Aggregate. *Proc. Phys. Soc. Section A* **1952,** *65* (5), 349.

29. Kim, S.-K.; Wie, J. J.; Mahmood, Q.; Park, H. S., Anomalous nanoinclusion effects of 2D MoS2 and WS2 nanosheets on the mechanical stiffness of polymer nanocomposites. *Nanoscale* **2014,** *6* (13), 7430-7435.

30. Lei, M.; Sarrao, J. L.; Visscher, W. M.; Bell, T. M.; Thompson, J. D.; Migliori, A.; Welp, U. W.; Veal, B. W., Elastic constants of a monocrystal of superconducting YBa2Cu3O7−δ. *Physical Review B* **1993,** *47* (10), 6154-6156.

31. Kimizuka, H.; Kaburaki, H.; Kogure, Y., Mechanism for Negative Poisson Ratios over the α- β Transition of Cristobalite, SiO_2: A Molecular-Dynamics Study. *Phys. Rev. Lett.* **2000,** *84* (24), 5548-5551.

32. Khan, U.; May, P.; O'Neill, A.; Bell, A. P.; Boussac, E.; Martin, A.; Semple, J.; Coleman, J. N., Polymer reinforcement using liquid-exfoliated boron nitride nanosheets. *Nanoscale* **2013,** *5* (2), 581-587.

33. Clyne, T. W., Materials Science II. Cambridge University Composite Materials Lecture Notes. **2014**.

34. Wei, Q.; Peng, X., Superior mechanical flexibility of phosphorene and few-layer black phosphorus. *Appl. Phys. Lett.* **2014,** *104* (25), 251915.

35. Wu, Z. C.; Chen, Z. H.; Du, X.; Logan, J. M.; Sippel, J.; Nikolou, M.; Kamaras, K.; Reynolds, J. R.; Tanner, D. B.; Hebard, A. F.; Rinzler, A. G., Transparent, conductive carbon nanotube films. *Science* **2004,** *305* (5688), 1273-1276.

36. Sheik-Bahae, M.; Said, A. A.; Wei, T.-H.; Hagan, D. J.; Van Stryland, E. W., Sensitive measurement of optical nonlinearities using a single beam. IEEE J. Quantum Electron**, 1990**, *26* (4), 760-769.

37. Wang, K.; Wang, J.; Fan, J.; Lotya, M.; O'Neill, A.; Fox, D.; Feng, Y.; Zhang, X.; Jiang, B.; Zhao, Q.; Zhang, H.; Coleman, J. N.; Zhang, L.; Blau, W. J., Ultrafast Saturable Absorption of Two-Dimensional MoS2 Nanosheets. *ACS Nano* **2013,** *7* (10), 9260-9267.
